# Genetic underpinnings of type-2 diabetes (T2D) with colorectal cancer (CRC): *In-silico* discovery of common molecular signatures, pathogenetic processes and therapeutic candidates

**DOI:** 10.1016/j.jgeb.2026.100667

**Published:** 2026-03-09

**Authors:** Reaz Ahmmed, Umme Samia Antu, Tasfia Noor, Md.Fahim Faysal, Arnob Sarker, Sabkat Mahmud, Alvira Ajadee, Md.Nurul Haque Mollah

**Affiliations:** a Bioinformatics Lab (Dry), Department of Statistics, Faculty of Science, University of Rajshahi, Rajshahi 6205, Bangladesh; b Department of Biochemistry & Molecular Biology, Faculty of Science, University of Rajshahi, Rajshahi 6205, Bangladesh; c Department of Biochemistry & Molecular Biology, Jahangirnagar University, Savar, Bangladesh; d Department of Computer Science and Engineering, Rajshahi University of Engineering & Technology (RUET), Rajshahi 6204, Bangladesh; e Dept. of Biomedical Informatics, Tulane University, 6823 St Charles Ave, New Orleans, LA 70118, United States

**Keywords:** Type-2 diabetes and colorectal cancer, Transcriptomics analysis, Shared key genes (sKGs), Shared drugs and toxicity, Bioinformatics approaches

## Abstract

Type-2 diabetes (T2D) is a risk factor for colorectal cancer (CRC) and the incidence rate of CRC in T2D patients is significantly higher than in control patients. It may be associated with the overlapping dysregulated genetic factors. The management of CRC becomes complicated with T2D compare to without T2D due to the conflict of therapeutic priorities for both diseases.​ However, studies on overlapping dysregulated genetic factors and their therapeutic priorities during their co-incidence/existence, are very limited. This study aimed to identify those overlapping dysregulated genetic factors with their functions, pathways and regulators through which T2D may stimulate the development and progression of CRC, for exploring effective shared drug therapies (polypharmacological agents) against both diseases, since a disease specific drug may conflict with other diseases during their co-existence. In order to explore repurposable common drugs for both T2D and CRC, we identified both diseases causing top-ranked 8 sKGs (CD44, COL18A1, CLDN5, PLS3, PTK2, THBS1, CAV1, and EFEMP1) as the drug targets through transcriptomics analysis. The relationship of sKGs with T2D and CRC were also verified through the literature review, expression analysis with independent datasets, functional enrichment analysis with KEGG-pathways and GO-terms, regulatory network analysis with microRNAs and transcription factors (TFs), DNA methylation and immune infiltration analysis based on independent databases. Finally, sKGs-guided top-ordered four candidate drugs (irinotecan, leucovorincalcium, regorafenib and Fenretinide) were recommended as the shared treatments for both CRC and T2D during their co-existence. The recommended drug therapy might be effective to reduce drug burden from the patients. Therefore, the findings demand experimental and clinical validations for taking a proper treatment plan against CRC with T2D as comorbidity.

## 1 Introduction

**Fig. 1:**
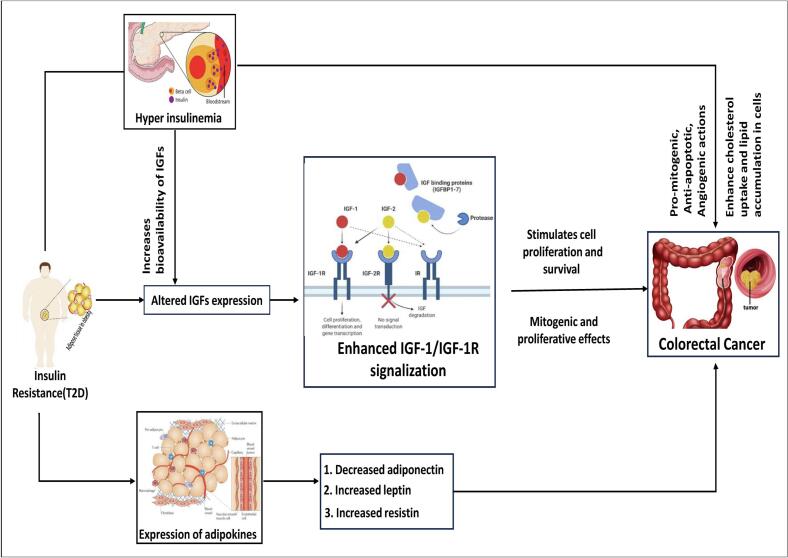
A diagram depicting the relationship between T2D and CRC. This figure represents how T2D may stimulate CRC.

Colorectal cancer (CRC) is a malignant tumor that develops from the inner lining of the colon or rectum within the gastrointestinal tract.^1^ It represents the second most prevalent malignancy among women and the third most common among men, accounting for the second highest proportion (9.0%) of cancer-associated mortality.^2^, ^3^ Annually, around one to two million individuals are newly diagnosed with CRC.^4^ The symptoms of CRC are not immediately apparent, which delays diagnosis and therapy.^1^ The CRC is common in those 65 to 74 years old, and it is more common in women.^5^ The CRC is proceeded by obesity and metabolic syndrome; approximately 80% of type-2 diabetes (T2D) patients are obese.^6^, ^7^ In recent decades, it has emerged as a significant global health burden and epidemic, largely attributed to unhealthy lifestyle factors. In 2013, it was estimated that approximately 382 million individuals worldwide were affected by diabetes.^8^ The incidence of T2D is growing gradually, and by 2035, it is expected that over 590 million.^9^ Generally, most of the elderly patients suffer from T2D. It is a metabolic disorder characterized by dysregulation of gene expression, as well as disturbances in glucose and lipid metabolism.^10^, ^11^ It generally arises as a consequence of insulin resistance (IR).^12^, ^13^ IR impairs the body’s ability to utilize glucose for energy production, resulting in persistently elevated blood glucose levels.^14^ It may also act as a linking factor by elevating circulating levels of insulin and insulin-like growth factor (IGF), both of which can facilitate tumor growth.^15^ Both normal colon epithelium and carcinoma cells proliferate by the exposing of extremely high levels of insulin and bioavailable IGF-I, which ultimately raised the risk of CRC.^3^, ^16^ The link between T2D and CRC is illustrated schematically in Fig. 1.

Literature review also indicated that T2D is linked to the initiation and progression of CRC^17^, ^18^, ^19^, ^20^ and this association is established due to their overlapping dysregulated genetic factors.^20^, ^21^ It should be mentioned here that Doctors may prescribes disease-specific multiple drugs to the patients due to the unavailability of alternative common/shared drugs (polypharmacological drugs).^22^ In that case, a drug for CRC may conflict with the T2D and vice-versa^23^, ^24^ and/or drug-drug interactions (DDI) during polypharmacy may create toxicity to the patients.^25^, ^26^ Therefore, physicians should prescribe shared drugs (polypharmacological drugs) against both conditions, thereby minimizing toxicity and reducing the overall drug burden for patients. However, investigation on overlapping dysregulated genetic factors and their inhibitors/activators as therapeutic priorities during their co-incidence/existence, are very limited. This study aimed to disclose those overlapping dysregulated genetic factors with their functions, pathways and regulators by which type 2 diabetes (T2D) may contribute to colorectal cancer (CRC) onset and advancement, for exploring effective common/shared drug molecules (polypharmacological agents) against both diseases.

There are two *in silico* approaches: de novo design and drug repurposing for identifying potential therapeutic molecules against these diseases. The de novo strategy typically requires greater financial investment, longer development time, and more intensive labor than the drug repurposing approach. The latter approach investigates existing medications from various diseases to identify their potential application in treating other conditions.^27^, ^28^ To identify drugs that can be repurposed for both T2D and CRC, it is essential to first determine the shared key genes (sKGs) implicated in both diseases, which may serve as targets for common therapeutic agents. Nevertheless, identifying the top-ranked shared key genes (sKGs) associated with both diseases from a vast pool of candidates is challenging using in vitro experimental approaches alone, as such methods are labor-intensive, time-consuming, and costly. Bioinformatics-based transcriptomic analysis is widely used to identify disease-associated key genes (KGs) as the targets of shared drugs.^29^, ^30^, ^31^, ^32^ Therefore, the primary objectives of this *in silico* computational bioinformatics study are to (i) identify overlapping dysregulated shared key genes (sKGs) between T2D and CRC as potential targets for common therapeutic agents through transcriptomic analysis, and (ii) determine sKG-guided repurposable candidate drugs that may serve as effective polypharmacological agents for the concurrent management of both diseases.

## 2 Materials and methods

This study analyzed gene expression datasets for T2D and CRC patients with control patients to explore both diseases related shared key-genes (sKGs). These sKGs were then used to explore unique/common candidate drugs for both diseases, as described below.

### 2.1 Data sources and their descriptions

Two microarray gene expression datasets for each of T2D and CRC were downloaded from the Gene Expression Omnibus (GEO) repository of the National Center for Biotechnology Information (NCBI). To validate the differential expression patterns of sKGs using independent datasets, an RNA-Seq expression profile dataset was obtained from The Cancer Genome Atlas (TCGA) database. The detail descriptions about these datasets were given in Table 1. To explore sKG-guided repurposable common drugs for both T2D and CRC, a total of 307 drugs associated with T2D and/or CRC were compiled by integrating drug lists from DrugBank, published literature, and the DrugMatrix database via the Enrichr plugin.^40^ (**Table S1**).

**Table 1:** The detail descriptions of transcriptomics (microarray and RNA-Seq) datasets.

**Accession IDs for the datasets from NCBI**	**Platforms**	**Data types**	**Cases**	**Control**
GSE29221^33^	GPL6947	Microarray	12(T2D)	12
GSE29226^34^	GPL6947	Microarray	12(T2D)	12
GSE20966^35^	GPL1352	Microarray	10(T2D)	10
GSE64998^36^	GPL11532	Microarray	07(T2D)	08
GSE18105^37^	GPL570	Microarray	94(CRC)	17
GSE22598^38^	GPL570	Microarray	21(CRC)	17
TCGA database^39^		RNA-Seq	481 (CRC)	41

### 2.2 Identification of shared key genes (sKGs)

T2D- and CRC-causing sKGs were detected through transcriptomics analysis. The detail procedures were discussed in the following four sub-2.2.1, 2.2.2, 2.2.3, 2.2.4**.**

#### 2.2.1 Differentially expressed genes (DEGs) identification

DEGs were detection from the microarray-derived gene expression datasets by comparing samples from the disease and control groups by using R-package of statistical LIMMA algorithm, since it performs well even with small sample size. It computes *P*-values to assess the significance of DEGs between two conditions, based on the moderated *t*-statistic.^41^ We also used DESeq2^42^ to detect DEGs from RNA-Seq profile dataset downloaded from TCGA database by an R/Bioconductor package named ‘TCGAbiolinks’. Then aLog_2_FCV (average of log_2_ fold-change values) and adj. *P*-value are combinedly employed to declare *k*th gene as an upregulated or downregulated DEG as detailed below:

$${DEG}_{k}=\left\{\begin{array}{c} {DEG}_{k}\left(Up\right),\;if\;adj.P.value<0.05anda{Log}_{2}\left({FCV}_{k}\right)>+1\\ {DEG}_{k}\left(Down\right),if\;adj.P.value<0.05anda{Log}_{2}\left({FCV}_{k}\right)<-1 \end{array}\right)$$

where $a{Log}_{2}\left({FCV}_{k}\right)$ is computed as

(1) $$a{Log}_{2}\left({FCV}_{k}\right)=\left\{\begin{array}{c} \frac{1}{m_{1}}\sum_{i}^{m_{1}}\log_{2}{(z}_{\mathrm{ki}}^{D})-\frac{1}{m_{2}}\sum_{j}^{m_{2}}\log_{2}{(z}_{\mathrm{kj}}^{C}),ifm_{1}\neq m_{2}\\ \frac{1}{m}\sum_{i}^{m}\log_{2}\left(\frac{z_{\mathrm{ki}}^{D}}{z_{\mathrm{kj}}^{C}}\right),ifm_{1}=m_{2}=m \end{array}\right)$$

Here $z_{\mathrm{ki}}^{D}$ and $z_{\mathrm{kj}}^{C}$ represents as the expression value of *k*th gene for the *i*th case (T2D andCRC) and *j*th control samples, respectively. Thus, the overlapping upregulated-(UP) and downregulated-(DR) DEGs for both conditions were computed.

#### 2.2.2 Comprehensive identification of shared DEGs (sDEGs) between T2D and CRC

We computed sDEGs between T2D and CRC as follows

$$sDEGs={sDEGs}^{UR}\cup {sDEGs}^{DR}$$

where,

$${sDEGs}^{UR}={DEGs}_{T2D}^{UR}\cap {DEGs}_{CRC}^{UR}$$

and

$${sDEGs}^{DR}={DEGs}_{T2D}^{DR}\cap {DEGs}_{CRC}^{DR}$$

Here ${DEGs}_{T2D}^{UR}and{DEGs}_{CRC}^{UR}$ denote the UR-DEGs for T2D and CRC, respectively, while ${DEGs}_{T2D}^{DR}and{DEGs}_{CRC}^{DR}$ represent the DR-DEGs for T2D and CRC, respectively. ‘∪’ and ‘∩’ symbols, respectively correspond to the union and intersection set operation.

#### 2.2.3 Local genetic inference of the association between T2D and CRC through sDEGs

The local genetic association between T2D and CRC was measured by using the simple correlation coefficient (r) based on aLog_2_FCVs of sDEGs, which is defined as follows

(2) $$r_{\mathrm{yz}}=\frac{\sum \left(y_{k}-\overset{¯}{y}\right)\left(z_{k}-\overset{¯}{z}\right)}{\sqrt{\sum ({y_{k}-\overset{¯}{y})}^{2}{\left(z-z\right)}^{2}}}$$

since sDEGs produce similar patterns of aLog_2_FCVs for both T2D and CRC, where, $y_{k}={aLog}_{2}{FCV(T2D)}_{k}$ and $z_{k}={aLog}_{2}{FCV(CRC)}_{k}$ are the aLog_2_FC values of the *k*^th^ gene for the two diseases T2D and CRC; $\overset{¯}{y}$ and $\overset{¯}{z}$ are the means of $y_{k}\prime s$ and $z_{k}\prime s$, respectively; k = 1, 2,…,*m.* Here m is the total number of sDEGs.^10^ .

#### 2.2.4 Identification of sKGs from sDEGs

Proteins execute their cellular functions through interactions with other proteins, and investigating protein–protein interaction (PPI) networks yields critical understanding of their biological significance^43^. In the present analysis, the STRING database was applied to build a PPI network of the sDEGs for the purpose of uncovering sKGs..^44^ Cytoscape software was employed to improve the visualization and interpretability of the PPI network^45^. The CytoHubba plugin in Cytoscape was applied to identify sKGs within the PPI network^45^, ^46^ Nodes in the PPI network indicate proteins, and edges describe how these proteins interact with each other. Top-ranked sKGs were determined according to their interaction profiles using six topological parameters: Stress, Closeness, Degree, MNC, EPC, and Radiality. Key modules/clusters in the protein–protein interaction (PPI) network were detected using the MCODE plugin in Cytoscape.^47^ The MCODE clustering algorithm was executed using a degree cutoff value 2, node score threshold of 0.2, k-core value of 2, and maximum depth of 100 to identify densely connected regions within the network.

### 2.3 *In-Silico* validation of T2D and CRC sKGs

To validate the association of sKGs with CRC and T2D, we performed comparative box plot analyses using transcriptomic data from NCBI, TCGA, and GTEx. This was supplemented by disease enrichment analysis to further investigate the functional link between these sKGs and the respective pathologies.

#### 2.3.1 Validation of sKGs by Box plot analysis

Differential expression patterns of sKGs in CRC and T2D were validated through **box plot analysis** using independent data from NCBI, TCGA, and GTEx, ensuring the robustness of our identified biomarkers. To verify the differential expression of sKGs (upregulated/downregulated) by the additional expression datasets, we considered UALCAN web-tool to construct Box plots of expression data of each sKGs with CRC and control groups, since this web-tool incorporates expression data from both TCGA (The Cancer Genome Atlas) and GTEx databases.^48^

We employed two independent datasets, GSE64998^36^ and GSE20966,^35^ utilizing NCBI-sourced data to substantiate the expression variance of sKGs observed between T2D cohorts and healthy controls.

#### 2.3.2 Regulatory network analysis of sKGs

The regulatory landscape governing sKGs was mapped by integrating TF and miRNA interactions from the RegNetwork repository.^49^ These interactions were analyzed through NetworkAnalyst^50^ and integrated into Cytoscape software^51^ to identify pivotal regulatory nodes and their downstream effects.

#### 2.3.3 Functional enrichment analysis of sKGs: GO terms and KEGG pathways

The biological significance of sKGs, including GO terms (BP, CC, MF) and pathways was assessed through enrichment studies based on GO keywords and KEGG pathways.^52^ A 2 × 2 contingency table (Table 2) was created to identify KEGG pathways and significantly GO terms associated with the sKGs set.

**Table 2:** sKGs-set enrichment analysis based on a 2 × 2 contingency table.

Status of Annotation	sKGs (proposed)	sKGs not involved	Total-Marginal
Annotated genes	*k_i_*	*A_i_ − k_i_*	*A_i_*
Not annotated	*n − k_i_*	*N − A_i_ – n + k_i_*	*N − A_i_*
Total marginal	*n*	*N − n*	*N* (Total-grand)

where *A_i_*: total number of genes with annotations in *i*th group (*i* = 1, 2,…,*r*); *N*: total number of genes with annotations in $A={⋃}_{i=1}^{r}A_{i}=A_{i}⋃A_{i}^{c}$ such that $N\leq \sum_{i=1}^{r}M_{i}.$ Here *n:* total count of sKGs, *k_i_*: number of sKGs belonging to *A_i_*. The DAVID database^53^ was employed to calculate p-values using Fisher’s exact test with a hypergeometric distribution,^54^ thereby uncovering a suite of overrepresented GO terms and KEGG pathways that underpin the biological roles of the sKGs.

#### 2.3.4 Survival analysis of CRC patients based on DNA methylation data

UALCAN^55^ and MethSurv^56^ database was used to investigate the DNA methylation of sKGs in CRC. This web-tool utilizes TCGA methylation data to investigate the methylation status. Methylation status was measured in terms of β-values, varying between 0 and 1. The β-values are found using the formula Me / (Me + Un + 100). Fully methylated and completely un-methylated intensities are denoted by the Me and Un, respectively.

#### 2.3.5 Immune infiltration analysis in CRC

A comprehensive tool for calculate the number of immune cell types that infiltrate tumors from TCGA data is the TIMER 2.0.^57^ The immunological infiltration levels of neutrophils, CD4 + T cells, B cells, CD8 + T cells, macrophages, and dendritic cells with sKGs in CRC were examined using TIMER's online tools.

### 2.4 The sKGs-guided drug repurposing

To identify repurposable common drug molecules for both T2D and CRC guided by shared key genes (sKGs), we conducted homology modeling, molecular docking, ADME/T, DFT, and MD simulations, as detailed in subsections **2.4.1 to 2.4.4**.

#### 2.4.1 The prediction of homology model

It was applied to predict the 3D conformations of specific proteins absent from the Protein Data Bank (PDB).^58^ SWISS-MODEL^59^ accepts a protein sequence (FASTA format) or its UniProt ID as input for 3D structure prediction. QMEAN^60^ and QMEANDisco^61^ analyses were performed to validate the accuracy of the predicted protein structure. ProCheck^62^ was used to generate the Ramachandran plot of the receptor protein to validate its 3D structure for molecular docking.

#### 2.4.2 Molecular docking for exploring sKGs-guided drugs

In order to investigate sKGs-guided drug molecules, we gathered 307 potential compounds from online databases and previous publications related to T2D and CRC (**Table S1)**. The 3D structure of each sKGs-mediated receptor protein were obtained from PDB^58^ or SWISS-MODEL.^59^ AutoDock Vina^63^ were used to pre-process the proteins by adding charges and lowering energy, respectively. Using AutoDock tools 1.5.6, the protein was prepped for molecular docking by eliminating ligand heteroatoms and water molecules and adding polar hydrogens.^64^ After downloading the 3D structures of all 307 drug molecules from the PubChem database,^65^ in preparation for molecular docking, AutoDock Tools version 1.5.6 was applied to assign the ligand’s rotatable/non-rotatable bonds and construct its torsion tree. The 3D structures of the pharmacological agents/ligands/drugs and receptor (proteins) are necessary for the molecular docking investigation. Avogadro software was used to perform energy minimization of the drug molecules. It assists in forecasting the stable structure of the molecule and how it engages with target sites.^66^ We employed MMFF94 as the force field. MMFF94 facilitates the generation of multiple molecular conformations by rotating bonds and modifying bond angles and lengths.^67^ Energy minimization was conducted through application of the steepest descent method. Drug-target protein binding affinities were then calculated using AutoDock Vina.^63^ The exhaustiveness parameter was defined as 8 during the docking process. For visualization and analysis, docking outputs were processed using BIOVIA Discovery Studio 2021. Receptor proteins and drug agents were prioritized according to their overall binding affinities to identify the top candidate drugs. Proteins were ranked based on their cumulative binding to all drugs, while drugs were ranked according to their total binding to all proteins. The top-ranked drug-agents were chosen as potential therapeutic candidates.

#### 2.4.3 ADME/T- analysis

The pharmacokinetic properties HIA ≥ 30%, LogBB ≥ 0.3, LC50 > 0 and LD50 > 0, absence of carcinogenic) of the docking score-based top-ranked drug compounds were examined by ADME/T analysis. The ADME/T analysis were carried out for each drug compounds using their optimized molecular structures with SMILES format. The online databases SwissADME,^68^ amdetSAR^69^ and pkCSM^70^ were used to analyses the ADMET features. Then the Lipinski rules were also investigated by using the SCFBio web-tool^71^ to assess the compliance of drug likeness properties (i.e., number of hydrogen bond acceptor < 10 & donor < 5, molecular weight < 500 Da, LogP < 5 value). The drug molecules that did not comply most of the pharmacokinetic properties and Lipinski rules were removed from the list of top-ranked drug molecules.

#### 2.4.4 DFT- analysis

Density functional theory (DFT), is a quantum mechanical modeling method, was used to investigate the electrical characteristics and relationship between ligands and receptors. The basic geometry of four best- hit compounds were retrieved from PubChem and minimized its energy by Avogadro. With the aid of the Gaussian 09 plugin in GaussView 05, the electrical and structural properties of the top-ordered four compounds were calculated using the B3LYP technique with a 6–311G basis set.^72^ Electrophilicity index, electron affinity, and the energies of the HOMO and LUMO are the calculated parameters used in this study. The significance of ligand-interaction in the binding pocket of the receptor protein can be understood mostly from these features.

#### 2.4.5 Molecular dynamic (MD) simulations

MD simulations were performed through the YASARA dynamic software^73^ and the AMBER-14 force field^74^ to investigate the dynamic properties of the optimal protein–ligand complexes. The most favorable protein–ligand complexes were taken for MD simulation. A TIP3P^75^ water model was used within the simulation cell to refine and stabilize the hydrogen bonding network of the protein–ligand complexes prior to simulation. This solvent density of 0.997 g L − 1 was maintained using periodic boundary conditions. A time-step interval of 2.50 fs was used for each simulation, which was conducted under physiological circumstances (pH-7.4, 298 K, 0.9% NaCl)^76^ using a multiple-time-step algorithm.^77^ MD simulations lasting 100 ns were conducted at constant pressure using the Berendsen thermostat.^78^ The YASARA^79^ macro's default script and the SciDAVis web tool were used for the first analysis, and the trajectories were recorded for further analysis every 250 ps. A number of graphs, including RMSD, RMSF, MM-PBSA, PCA, radius of gyration (Rg), SASA, and DCCM were examined to verify the complexes' stability and flexibility. The binding free energy were computed for each snapshot using the MM-PBSA (MM-Poisson–Boltzmann surface area) formula^80^. MM-PBSA binding energies were determined in YASARA using its built-in macros and AMBER 14 force field.

### 2.5 Schematic Representation of the Multi-Stage research design

**Fig. 2:**
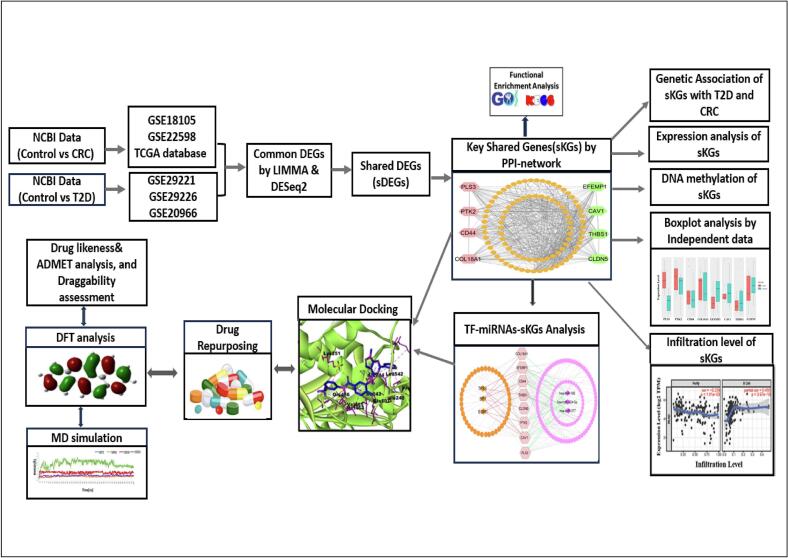
Systematic Workflow of this study.

The workflow of revealing repurposable common drugs for T2D and CRC through transcriptomics analysis was displayed in Fig. 2.

## 3 Results

### 3.1 Identification of T2D- and CRC-causing sKGs

T2D- and CRC-causing sKGs were detected through transcriptomics analysis. The detail analyses procedures were discussed in the following four sub-3.1.1, 3.1.2, 3.1.3, 3.1.4**.**

#### 3.1.1 Identification of DEGs

At first 897, 835 and 809 number of DEGs between T2D and control samples were detected from three gene-expression profiling datasets with NCBI accession IDs GSE29221, GSE29226 and GSE20966, respectively by combining the *P*-values of statistical LIMMA approach and the average of log2-fold change values (aLog_2_FCV) with the cut-off at *P*.values < 0.05 and |aLog2FC|>1. Then 1075 common DEGs between T2D and control samples were detected from those three DEGs-sets, where 200 DEGs were upregulated with aLog2FC > 1 and 875 downregulated DEGs with aLog2FC < -1 (**Table S2**). In the case of the dataset of CRC with NCBI accession ID GSE22598 was balanced between CRC and control samples. From this dataset, 29,926 DEGs were identified by the LIMMA approach. However, the datasets GSE18105 and the large cohort TCGA RNA-Seq dataset (Table 1) were unbalanced between CRC and control samples. To identify DEGs from the unbalanced dataset, we created some balanced datasets from the unbalanced dataset, since DEGs-set from the unbalanced dataset significantly differs from the DEGs-sets of balanced datasets. To create 5 balanced datasets between CRC and control samples from the unbalanced dataset with NCBI accession ID GSE18105, we randomly partitioned the CRC samples in 5 groups of sizes 18–19 samples without replacement. Similarly, we created 12 balanced datasets from the unbalanced TCGA RNA-Seq profile data set. Then we computed DEGs-set between CRC and control samples from each of 18 balanced datasets and found 4855, 4552, 4295, 4478, 4235, 9340, 8004, 8100, 8531, 7558, 9182, 8561, 8325, 9154, 8559, 7851, 8007, and 8327 numbers of DEGs in 18 DEGs-sets, respectively by combining the *P*-values of statistical LIMMA and DESeq2 approaches and aLog_2_FCV with the cut-off at *P*.values < 0.05 and |aLog2FC|>1. Finally, 2355 common DEGs between CRC and control samples were detected from those 18 balanced datasets, where 1,323 DEGs were upregulated and 1,032 downregulated DEGs (**Table S3**).

#### 3.1.2 Identification of sDEGs between T2D and CRC

Let ${|DEGs}_{T2D}^{UR}\left|and{|DEGs}_{CRC}^{UR}\right|$ indicates the number of UR- DEGs for both T2D and CRC, respectively. In contrast, $|{DEGs}_{T2D}^{DR}\left|and{|DEGs}_{CRC}^{DR}\right|$ represents the number of DR- DEGs for T2D and CRC, respectively. Then from section 3.1^,^ ${|DEGs}_{T2D}^{UR}|=200$, $|{DEGs}_{T2D}^{DR}|=875$, ${|DEGs}_{CRC}^{UR}|=1323$ and $|{DEGs}_{CRC}^{DR}|=1032$. Then the number shared upregulated DEGs between T2D and CRC was$|{sDEGs}^{UR}\left|=\right|{DEGs}_{T2D}^{UR}\cap {DEGs}_{CRC}^{UR}|$ = 52, and the number shared downregulated DEGs between T2D and CRC is |${sDEGs}^{DR}|=\left|{DEGs}_{T2D}^{DR}\cap {DEGs}_{CRC}^{DR}\right|=92$(**Table S4).** Thus, sDEGs between T2D and CRC were computed as $\left|sDEGs\right|=\left|{sDEGs}^{UR}\cup {sDEGs}^{DR}\right|=104$ (**Table S5).**

#### 3.1.3 Local genetic inference of the association between T2D and CRC through sDEGs

We used equation (2) to calculate the local correlation coefficient between T2D and CRC based on the aLog2FC values of sDEGs in order to determine the relationship between the both diseases. T2D and CRC are locally related with one another through the expression of sDEGs, according to the correlation coefficient, which was reported to be 0.81.

#### 3.1.4 Identification of shared key genes (sKGs)

**Fig. 3:**
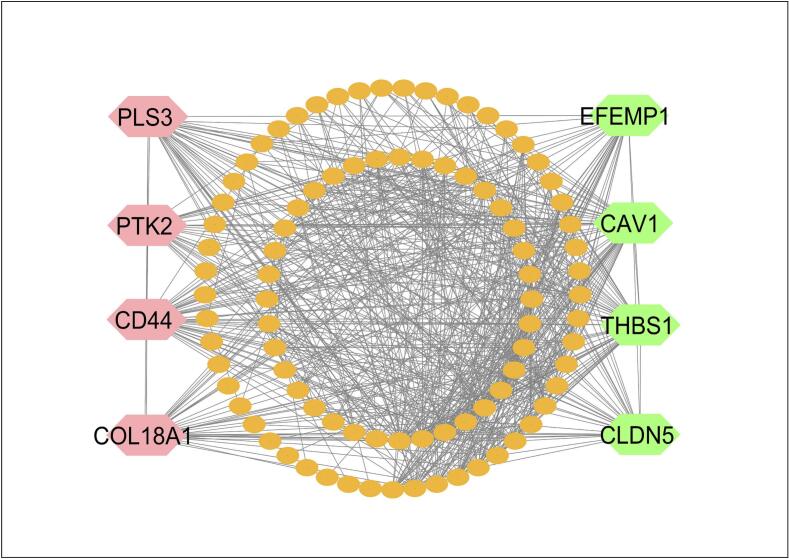
A PPI network of sDEGs to identify the sKGs, whereas the chocolate pink colour nodes indicated the upregulated sKGs and turquoise blue colour indicated the downregulated sKGs. (For interpretation of the references to colour in this figure legend, the reader is referred to the web version of this article.)

The PPI network of sDEGs, which consists of 78 nodes and 156 edges, was constructed using the STRING database **(**Fig. 3**)**. We selected the top ranked eight sKGs (CD44, COL18A1, CLDN5, PTK2, THBS1, CAV1, EFEMP1, and PLS3) based on six topological parameters (Stress, Degree, Closeness, EPC, MNC, and Radiality) in the PPI network (**Table S6**).

### 3.2 In*-Silico* validations on the association of sKGs with T2D and CRC by using independent datasets and databases

To assess the relationship of sKGs with T2D and CRC using independent datasets and databases, we carried out expression analysis, functional enrichment, regulatory network analysis with TFs and microRNAs, along with immune infiltration and DNA-methylation studies, as presented in subsections **3.2.1–3.2.5**.

#### 3.2.1 Expression analysis of sKGs with T2D and CRC based on independent datasets

Based on the independent gene expression profiles from the TCGA and GTEx datasets, which included 286 CRC and 41 control samples, we used Box-plot analysis to examine the differences in sKG expression patterns between CRC and control samples. Our proposed four sKGs (THBS1, CAV1, EFEMP1, and CLDN5) are downregulated and the other four sKGs (CD44, COL18A1, PLS3, and PTK2) are upregulated (**Fig. S1A)**. Additionally, using Box-plot analysis based on separate gene expression profiles from the NCBI-database with accession ID GSE64998,^36^ we examined the differences in sKG expression patterns between T2D (case) and control samples. In our analysis, we looked at between disease and control samples**,** the four sKGs (THBS1, CAV1, EFEMP1, and CLDN5) are downregulated in T2D, while the other four (CD44, COL18A1, PLS3, and PTK2) are upregulated **(Fig. S1B)**.

#### 3.2.2 Regulatory network analysis of sKGs

**Fig. 4:**
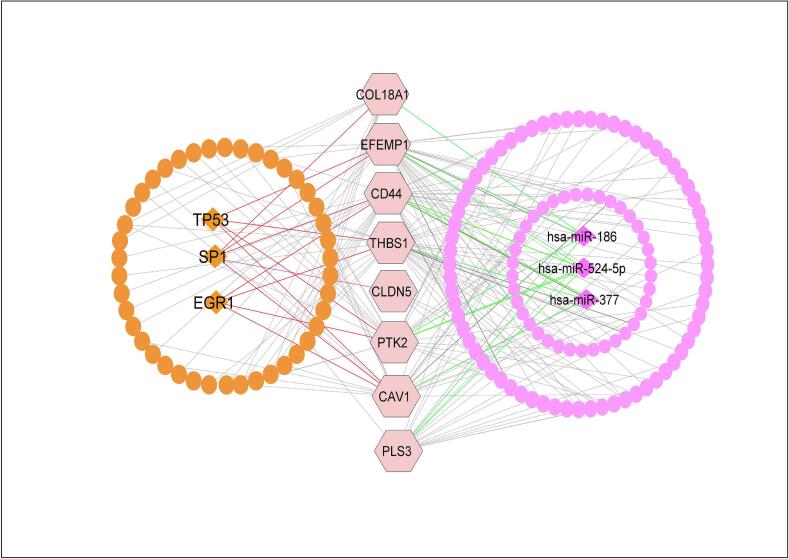
Regulators of sKGs identification by network analysis. The sKGs with TFs-miRNA interaction network analysis results based on the JASPAR and TarBase databases, respectively, where sKGs were labeled in chocolate pink color with hexagon shape. The top-ranked TFs and miRNAs regulators of sKGs were displayed by orange and pink color diamond-shaped, respectively. (For interpretation of the references to colour in this figure legend, the reader is referred to the web version of this article.)

The top-ranked three significant TFs (diamond shape-orange color) EGR1, SP1 and TP53 were identified as the main transcriptional regulatory factors for sKGs by the network analysis of sKGs with TFs. Similar to this, three important miRNAs (diamond shape-pink color), designated as hsa-miR377, hsa-miR-539 and has-miR135b, were found by network analysis of sKGs with miRNAs **(**Fig. 4**).**

#### 3.2.3 Functional enrichment analysis of sKGs: GO terms and KEGG pathways

To explore the shared pathogenic mechanisms between T2D and CRC, we conducted GO and KEGG pathway enrichment analyses for eight sKGs. The top-ranked 5 each type of GO-terms and 6 KEGG-pathways are given in Table 3. The sKGs linked to the pathogenic mechanisms of T2D in CRC were found to significantly enhance KEGG-pathways and GO-terms, along with corresponding sDEGs.

**Table 3:** The topmost five significantly enriched GO terms and Bioplanet pathways associated with sKGs, as identified using the Enrichr web tool.

GO-terms	**ID**	**Enriched GO-Terms**	**Number of Enriched sDEGs**	***P.v*alue**	**Enriched sKGs**
Biological process(BPs)	GO:0008284	“Positive regulation of cell proliferation”	11	0.00307245	PTK2, THBS1
	GO:0001525	“Angiogenesis”	7	0.00197691	COL18A1, CAV1, PTK2
	GO:0030335	“Positive regulation of cell migration”	8	0.00187244	CAV1, CLDN5, THBS1, PTK2
	GO:0030154	“Cell differentiation”	11	0.01013828	CAV1, PLS3, PTK2
	GO:0051897	“Positive regulation of protein kinase B signaling”	7	0.00136269	THBS1, PTK2
Molecular Function (MFs)	GO:0016504	“Peptidase activator activity”	4	0.0000692	CAV1
	GO:0030297	“Transmembrane receptor protein tyrosine kinase activator activity”	4	0.0089288	CLDN5, CD44
	GO:0005518	“Collagen binding”	5	0.00129345	CD44
	GO:0005178	“Integrin binding”	6	0.00525727	THBS1, PTK2,
	GO:0051015	“Actin filament binding”	7	0.001466	PLS3
Cellular Components (CC)	GO:0062023	“Collagen-containing extracellular matrix”	16	9.95E-10	COL18A1, THBS1, EFEMP1
	GO:0031012	“Extracellular matrix”	9	0.000252	EFEMP1, THBS1
	GO:0070062	“Extracellular exosome”	28	0.000758	THBS1, EFEMP1, CD44
	GO:0005938	“Cell cortex”	6	0.00586012	CAV1, PTK2, CLDN5
	GO:0005925	“Focal adhesion”	9	0.00654495	CAV1, FLNC, CD44, PTK2
KEGG Pathways/ BioPlanet Pathways	hsa04380	“Osteoclast differentiation”	22	4.78E-12	CSF1R, JUN, TYROBP, IL1B
	hsa04010	“MAPK/ERK pathway”	9	1.71E-04	CD44, CSF1R, IL1B
	hsa04060	“Cytokine-cytokine receptor interaction”	20	1.38E-04	CSF1R, CXCL8, IL1B, CCR5
	hsa05205	“Proteoglycans in cancer”	6	0.0139123	CAV1, THBS1, CD44, PTK2
	hsa04151	“PI3K/AKT signaling pathway”	13	1.94E-05	COL18A1, THBS1, IL1B, CD44, TLR2
	hsa04350	“TGFβ-signaling pathway”	8	0.00506812	EFEMP1, THBS1

#### 3.2.4 Survival analysis of CRC patients based on DNA methylation data

An epigenetic mechanism called DNA methylation controls gene expression by either preventing transcription factors from binding to DNA or by activating proteins associated in gene repression.^55^ DNA hypermethylation, mainly at the CpG islands in a gene's promoter region, facilitates significant silence of tumor suppressor genes. Conversely, DNA hypomethylation results in the upregulation of oncogenes.^81^ As a result, we used methsurv database to analyze the DNA methylation status at CpG sites for the sKGs (CD44, COL18A1, CLDN5, PLS3, PTK2, THBS1, CAV1, and EFEMP1). Six sKGs (CD44, PTK2, CAV1, EFEMP1, PLS3 and CLDN5) were found to have significant CpG sites (*p*-value ≤ 0.05) **(Table S7)**. The six sKGs in CRC were also examined for promoter methylation status using UALCAN. According to β-values, which range from 0 (totally unmethylated) to 1 (extremely methylated), three sKGs (CD44, PTK2 and PLS3) were found to be hypomethylated based on the Box whisker plot. This is compelling evidence that three sKGs were increased in colorectal cancer.

#### 3.2.5 Exploring different immune infiltration levels in CRC by the association of sKGs

The complex environment known as the tumor microenvironment (TM) is made up of both tumor cells and various stromal components, such as immune cells.^82^ We are analyzing the relationships between the expression levels of the sKGs and the infiltration levels of 6 immune cells (B cell, neutrophil, CD4 + T cell, CD8 + T cell, dendritic cell, and macrophage) in order to predict the infiltration of immune cells in colorectal cancer (CRC) using the TIMER algorithm (**Fig. S2**). The results show that sKGs expression has a positive and strong correlation with CD4 + T cell infiltration level (0.08 ≤ Rho ≤ 0.45) as well as Macrophage (0.587 ≥ Rho ≥ 0.097) and weakly and negative correlated with CD8 + T cell infiltration level (−0.059 ≥ Rho ≥ 0.28), B cell (0.036 ≥ Rho ≥ -0.076), neutrophil (0.514 ≥ Rho ≥ -0.068), and dendritic cell (0.643 ≥ Rho ≥ -0.022). This result might be help find potential immunotherapy for CRC.

### 3.3 Molecular docking for exploring sKGs-guided drugs

To explore potential sKGs-guided repurposable drugs for T2D and CRC, we performed molecular docking along with ADME/T and DFT studies, as described in subsections **3.3.1–3.3.3.**

#### 3.3.1 The prediction of homology model

By applying SWISS-MODEL, the three-dimensional structures of CLDN5, EFEMP1, and PTK2 were obtained, with their GMQE scores calculated as 0.70, 0.77, and 0.72. Typically, a GMQE score above 0.70 is regarded as a reliable indicator of model quality.^83^ Moreover, the crystal structures of CLDN5, EFEMP1, and PTK2 showed sequence identities of 81%, 99%, and 99% with their respective template proteins. The QMEANDisCo scores of CLDN5, EFEMP1, and PTK2, obtained using SWISS-MODEL, were 0.47 ± 0.05, 0.43 ± 0.05, and 0.45 ± 0.07, respectively. ProCheck evaluated the structural quality of the protein by generating a Ramachandran plot, which analyzes phi and psi dihedral angles. As shown in **Fig. S3**, all residues occupied the favored regions (89.7%, 87.78%, 86.93%), and no residues were found in the disallowed regions. Therefore, the model was validated as reliable and utilized for further studies on ligand–receptor interactions.

#### 3.3.2 Molecular docking for exploring sKGs-guided drugs

Our suggested eight sKGs and their regulatory three TFs has taken into consider as receptors in order to investigate potential ligands (drug molecules) for the treatment of T2D and CRC. To determine the 3D structure of these receptors, we collected their structures from two different sources. Eight receptors CD44, COL18A1, THBS1, CAV1, EGR1, SP1 TP53 and PLS3 were searched for their structures in the PDB using the following PDB codes: 4PZ4, 3HSH, 2ERF, 7SC0, 4R2A, 1SP1, 8DC8, and 1WJO. On the other hand, the remaining three receptor CLDN5, EFEMP1 and PTK2 were searched for their structures in the SWISS-MODEL database. Using the molecular docking analysis, binding affinity scores or BAS between the candidate drug compounds and the suggested receptors were computed. Based on the average BAS, drug compounds were arranged to choose the best therapeutic candidates. Similarly, receptor proteins were arranged according to the average BAS (**Table S8**). According to the sorted receptors, the top 30 ordered drug compounds were visualized in **Fig. S4**. From this figure, we considered top-ordered seven drug molecules (Irinotecan, Leucovorincalcium, Regorafenib, Fenretinide, Ouabain, doxorubicin and gentamicins) that satisfy the cut-off at average BAS > −7.0 Kcal/mol, for further *in-silico* validation against both T2D and CRC. To verify the performance of the proposed drug molecules against the top-ranked T2D-causing key genes/proteins as well as the top-ranked CRC-causing key genes/proteins, computationally, we performed molecular docking analysis and found their significant BAS scores **(**Figs. S5-S6 and Tables S11-S12**)**. Thus, *in-silico* studies suggested that the proposed drugs might be effective against both diseases**.** We also computed BAS with some equal expressed genes or, unregulated genes that are not associated with T2D and CRC and found smaller BAS compare to the sKGs (**Table S13)**. In order to compare BAS of the identified top-ranked 7 drugs with BAS of the control ligands of T2D and CRC against the proposed receptors, we considered three reference ligands (Glibenclamide, Metformin, Gliclazide) for T2D and three ligands (Irinotecan Hydrochloride, Capecitabine, Fruquintinib) for CRC. We observed that their BAS scores (**Table S9** and **Table S10)** are almost similar to the proposed ligand-target complexes.

#### 3.3.3 Re-docking of the co-crystallized ligand and calculation of RMSD

**Fig. 5:**
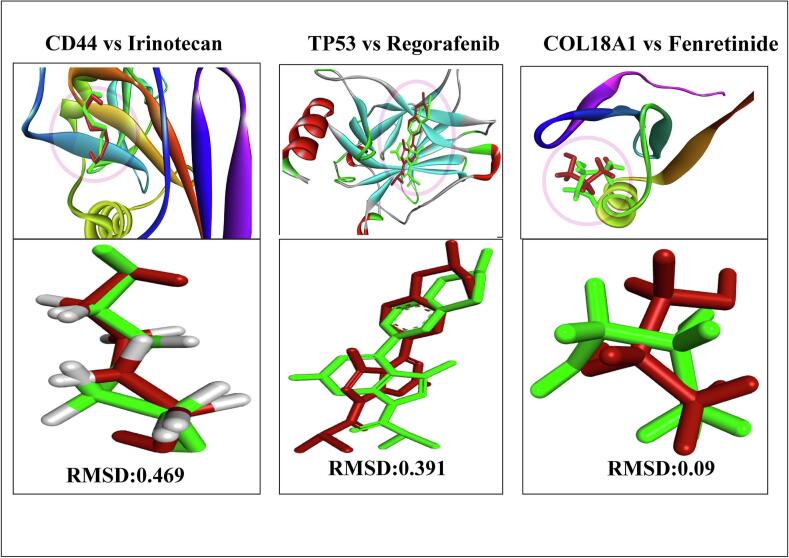
To validate the docking protocol, the co-crystallized ligands from CD44, TP53, and COL18A1 (PDB IDs: 4PZ4, 8DC8, and 3HSH) were re-docked. A close resemblance was observed between the docked ligand (red) and its crystal structure counterpart (green), with RMSD values of 0.469 Å, 0.391 Å, and 0.09 Å. (For interpretation of the references to colour in this figure legend, the reader is referred to the web version of this article.)

Validation of docking is generally performed by re-docking the ligand originally present in the PDB complex into the active site and calculating the RMSD with respect to its crystallographic conformation. This evaluation determines how closely the docked poses match the experimentally observed ligand conformation. The co-crystal ligand is redocked into its binding pocket, and the deviation from its experimentally determined pose is quantified through RMSD analysis. As shown in Fig. 5, the RMSD of the co-crystallized ligand was measured to evaluate the two poses, confirming that all molecules fell within the acceptable range, with RMSD values under 2.0 Å. Re-docking of the co-crystal ligands yielded RMSD values of 0.469 Å for Irinotecan, 0.391 Å for Regorafenib, and 0.09 Å for Etoposide.

#### 3.3.4 ADME/T- analysis

**Fig. 6:**
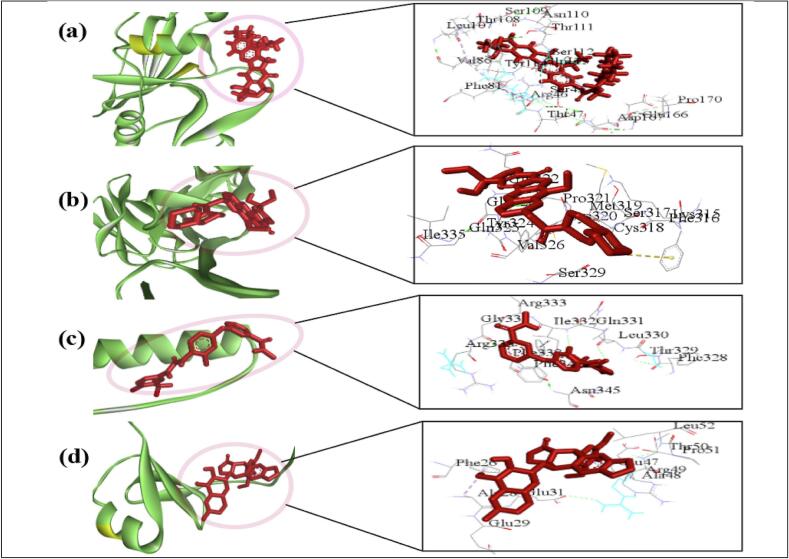
Some of the important docking results with the ligand- protein interactions. The four highest-ranked drug–target complexes, showing their 3D- structures and key interacting residues. Complexes: (a) indicated CD44-Irinotecan, (b) EFEMP1-Leucovorincalcium, (c) TP53-Regorafenib, and (d) COL18A1-Fenretenide.

At first drug likeness properties of the top-ranked seven drug molecules (Irinotecan, Leucovorin calcium, Regorafenib, Fenretinide, Ouabain, doxorubicin and gentamicins) were investigated using Lipinski rule. We found that the molecule leucovorin calcium has no violation, and irinotecan, regorafenib and fenretinide violate only one rules. The other three molecules (Ouabain, doxorubicin and gentamicins) violate more than one drug likeness properties **(**Table 4A**)**. The pharmacokinetics properties of those seven drug molecules were investigated using ADME/T analysis. The compounds are expected to be an appropriate oral medication candidate due to their adequate absorption in the GIT. A drug compound is considered to have high absorption in the human GIT if its Human Intestinal Absorption (HIA) score is above 30% (HIA ≥ 30%).^84^ We observed that four drugs (Irinotecan, Leucovorin calcium, Regorafenib, Fenretinide) have a high HIA score (≥ 85%), indicating good absorption in the human body. On the other hand, the rest three drugs Ouabain, doxorubicin and gentamicins produce HIA score ≤ 30% **(**Table 4B**)**. But the blood–brain barrier (BBB) can be easily and potentially crossed by compounds with a LogBB ≥ 0.3, but compounds with a LogBB < −1 are thought to be poorly disseminated via the BBB. The evaluation of seven compounds have limited BBB crossing capabilities. Following the seven drugs Irinotecan, Leucovorin calcium, Regorafenib, Fenretinide, Ouabain, doxorubicin and gentamicins LD_50_ values are s 2.81, 2.64, 2.11, 2.31,2.64, 2.40, 2.59 respectively. Drug molecules are less toxic when their LC50 value is higher; conversely, a smaller value denotes increased toxicity. The LC_50_ value for seven drugs (Irinotecan, Leucovorin calcium, Regorafenib, Fenretinide, Ouabain, doxorubicin and gentamicins) are 0.78, 6.79, 0.53, 1.02, 1.89, 0.41 and 0.24 respectively. Thus, we observed that top-ranked 4 drugs (Irinotecan, Leucovorin calcium, Regorafenib and Fenretinide) comply most of the pharmacokinetic properties and Lipinski rules. Consequently, these four drugs were selected for further analysis. The 3D visualization of the four drug molecules and their complexes is provided in Fig. 6 and **Table S14**. Hence, the four selected drugs are likely to inhibit the genes involved in the development of both T2D and CRC accordingly.

**Table 4:** Results of (A) drug-likeness and (B) ADME/T analyses.

**A.** Drug likeness profile of candidate drug molecules														
Name of compounds		Molecular weights			Log P		H-bond Acceptor(HBA		H-bond donor(HBD		Polar Surface area (Å^2^		Violation	
Irinotecan		586.689			4.0911		9		1		249.756		1	
Leucovorincalcium		473.44			−3.7813		8		5		227.833		0	
Regorafenib		482.82			5.6888		4		3		189.277		1	
Fenretinide		391.555			6.862		2		2		174.782		1	
Ouabain		584.659			−1.515		12		8		238.089		3	
Doxorubicin		543.525			0.0013		12		6		222.081		3	
Gentamicins		477.603			−3.3275		12		8		194.977		2	
**B.** ADME/T properties of the top-ranked seven drugs														
Name of Compounds	Absorption			Distribution				Metabolism		Excretion	Toxicity			
	Caco2 Permeability	HIA (%)	P-gpI	BBB(LogBB)		CNS (LogPS)		CYP3A4 Inhibitor		Total clearance (TC)	AMES	LC_50_(log mM)		LD_50_(mole/kg)
				(Permeability)										
Irinotecan	0.648	99.87	Yes	−1.303		−3.232		Yes		0.939	No	0.78		2.81
Leucovorincalcium	−0.91	86.61	No	−1.849		−4.695		No		0.595	No	6.79		2.64
Regorafenib	0.706	88.74	Yes	−1.676		−2.064		Yes		−0.042	No	0.53		2.11
Fenretinide	1.248	89.18	Yes	0.182		−1.463		Yes		1.102	No	1.02		2.31
Ouabain	0.332	20.87	No	−1.285		−3.881		No		0.726	No	1.89		2.641
Doxorubicin	0.457	28.37	No	−1.379		−4.307		No		0.987	No	0.41		2.408
Gentamicins	0.979	19.16	No	−0.851		−4.093		No		0.708	No	0.24		2.559

#### 3.3.5 DFT- analysis

Four selected compounds based on their drug-likeness and ADMET analyses, were assessed further through DFT analysis. The frontier molecular orbital (FMO) characteristics of the four compounds were analyzed to evaluate the role of charge-transfer interactions within the protein’s binding site. FMOs are used to define different kinds of reactions and predict which regions of molecular structures will become more reactive. These FMO characteristics, such as the HOMO and LUMO orbitals, are incredibly valuable for characterization of material science since they reveal the reactivity of the chemical molecule. The HOMO and LUMO analyses indicated that the molecule is extremely susceptible to charge transfer. A molecule with a high HOMO value has a strong electron donor, whereas one with a low HOMO value has a weak electron acceptor. Before docked the HOMO and LUMO orbital distributions are given (**Fig. S7** and **Table S15).** After docked and after simulation the HOMO and LUMO orbital distributions are given (Fig. S8 & Table S16**)** and **(Fig. S9** & **Table S17)** respectively. A lower distance between the HOMO and LUMO energies also has an important impact on how molecules exchange charges with one another and compounds bioactivity. As a result, the hit molecules huge energy gap prevents the electron moving from the HOMO to the LUMO, which lowers the inhibitor's affinity for CRC with T2D. Before docking the energy gap of irinotecan, Leucovorin calcium, Regorafenib, Fenretinide are 0.12781, 0.00952, 0.17968 and 0.11665 eV respectively, while the HOMO and LUMO energies are −0.20987, −0.08206, −0.06731, −0.05779, −0.23652, −0.05684, −0.19065 and −0.07400 eV, respectively **(Table S12)**. But after docking and simulation these values are slightly changed **(Table S14 and Fig. S8) and (Table S15 and Fig. S9)**. The chemical potential (μ) indicates negative values for all compounds, indicating excellent stability and the formation of a strong receptor complex.

#### 3.3.6 MD simulations

**Fig. 7:**
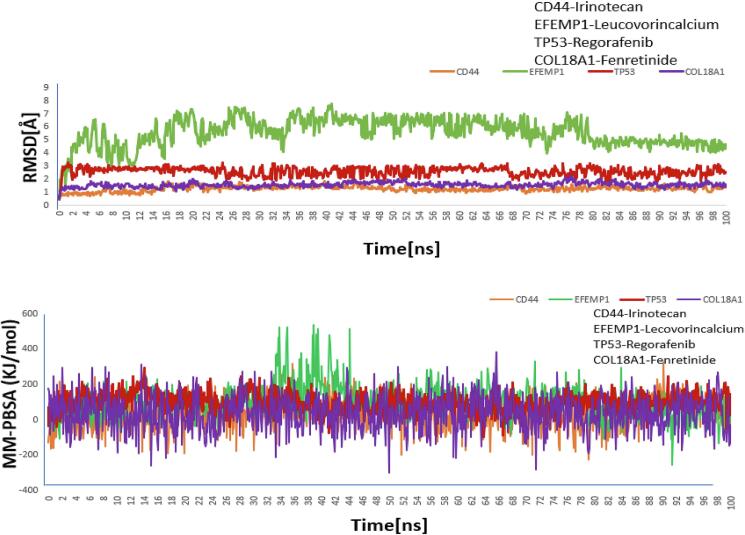
(A) The time-dependent RMSD profiles of the protein backbone atoms (C, Cα, and N) for each docked complex. (B) Using by the molecular mechanics Poisson–Boltzmann surface area (MM-PBSA) analysis, the binding free energy (in kJ mol − 1) of each snapshot was determined. Higher positive values indicate stronger binding and show changes in binding stability for each complex over the course of the simulations. The complexes are orange for CD44, green for EFEMP1, cherry red for TP53 and purple for COL18A1. (For interpretation of the references to colour in this figure legend, the reader is referred to the web version of this article.)

The top four recommended candidate drugs were Irinotecan, Leucovorincalcium, Regorafenib and Fenretinide. Consequently, 100 ns MD-based MM-PBSA simulations were used to confirm the stability of these top four drugs. All four systems were substantially stable between the original drug-target and variants of moving complexes, according to Fig. 7. The root mean square deviation (RMSD) for the proposed receptors (CD44, EFEMP1, TP53 and COL18A1) was displayed in Fig. 7A. For the CD44, EFEMP1, TP53 and COL18A1 complexes, the average RMSD were 1.246 Å, 5.535 Å, 2.564 Å and 1.533 Å respectively. For the four drug agents listed above, we calculated the MM-PBSA binding energy. The binding energy for the top four suggested receptors (CD44, EFEMP1, TP53 and COL18A1) is displayed in Fig. 7B. The binding energies of the CD44, EFEMP1, TP53 and COL18A1 complexes were, on average, 27.825 kJ/mol, 94.951 kJ/mol, 93.964 kJ/mol, and 33.829 kJ/mol, respectively. The Root Mean Square Fluctuation (RMSF) for the proposed receptors (CD44, EFEMP1, TP53 and COL18A1) was displayed in **Fig. S10**. The proposed protein CD44, EFEMP1, TP53 and COL18A1 complexes, the average RMSF were 1.653 Å, 1.155 Å, 3.67 Å and 2.695 Å respectively. The Solvent-Accessible Surface Area (SASA) for the proposed receptors (CD44, EFEMP1, TP53 and COL18A1) was displayed in **Fig. S11**. The proposed protein CD44, EFEMP1, TP53 and COL18A1 complexes, the average SASA were 8373.986, 10494.873, 3563.355 and 4658.427 respectively. The Radius of Gyration (Rg) for the proposed receptors (CD44, EFEMP1, TP53 and COL18A1) was displayed in **Fig. S12**. The proposed protein CD44, EFEMP1, TP53 and COL18A1 complexes, the average Rg were 15.73 nm, 12.77 nm, 12.94 nm and 16.51 nm respectively. The Dynamic Cross-Correlation Matrix (DCCM) is displayed using a color scale, where blue indicates complete anti-correlation (−1) and yellow represents complete correlation (+1). It was possible to see that the associated movements represented by each complex differed significantly after looking at the DCCM diagrams of the four systems in **Fig. S13**. PCA, a statistical technique, is employed to identify and extract the most prominent dynamic motions in simulations. These computations were essential for evaluating the stability of each complex, with lower PCA value distributions reflecting greater stability (**Fig. S14)**. The above analysis clearly indicates that all the complexes exhibited structural stability.

## 4 Discussion

**Fig. 8:**
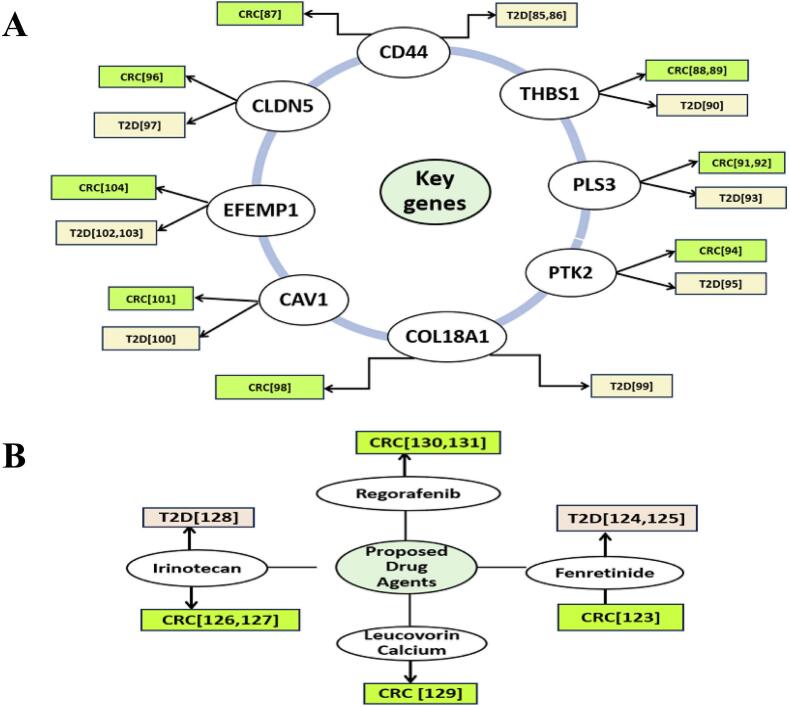
Literature-based validation of the proposed sKGs and corresponding drug agents for T2D and CRC, including **(A)** validation of the proposed drug targets and **(B)** validation of the recommended drug agents.

Evidence from population-based studies suggests that T2D represents a prevalent risk factor for CRC.^85^, ^86^ To investigate their relationship from the genetic viewpoints, we identified 104 sDEGs that can distinguish both T2D and CRC samples from controls, where 52 sDEGs are upregulated and 92 sDEGs are downregulated. The local correlation coefficient between both T2D and CRC was determined from sDEG aLog2FC values using equation (2), yielding *r*_xy_ = 0.81, indicating a genetic link between these diseases. Therefore, in this study, we attempted to explore sDEGs-guided common drugs for both (T2D- and CRC) to reduce the toxicity of DDI due to polypharmacy. To find the most representative of sDEGs, we identified top-ordered eight sDEGs (CD44, THBS1, PLS3, PTK2, CLDN5, COL18A1, CAV1, and EFEMP1) as the sKGs by constructing PPI network of sDEGs **(**Fig. 3**)**. Then the relationship of sKGs between both T2D and CRC were verified by the literature review, expression level analysis based on independent datasets, functional enrichment analysis with KEGG-pathways and GO-terms, TFs and microRNAs regulatory network analysis, immune infiltration and DNA methylation were carried out based on independent datasets, as outlined below. Through enrichment analysis of the sKGs-set, we examined KEGG pathways, Go terms (BPs, CCs, and MFs) linked to the onset of T2D and CRC (Table 2). The CRC and T2D that were enriched through BPs, CCs, MFs and KEGG pathways i.e., positive regulation of cell proliferation, cell differentiation, extracellular exosome, peptidase activator activity, MAPK/ERK pathway, PI3K/AKT signaling pathway etc. Some previous studies indicated that those process, function and pathways are highly linked with the development of both T2D and CRC. Among them, CRC is caused by an imbalance in BP (e.g. positive regulation of cell proliferation) which are induced by particular mutations. When proliferation exceeds apoptosis in the colon lumen, benign protrusions known as polyps.^87^ The MF (e.g. peptidase activator activity) are important tools in developing clinical strategies for cancer treatment and monitoring. In CRC, the adenoma carcinoma sequence of the large intestine represents the progressive transformation of normal epithelium into dysplastic epithelium, ultimately culminating in carcinoma. This progression occurs through the stepwise build-up of genetic mutations that modify the expression of different glycoproteins, including specific peptidases.^88^ Among them, CCs (e.g. collagen-containing extracellular matrix) are significantly associated with development and progression of CRC. Numerous proteins contribute to the extracellular matrix (ECM), the most important of which are collagen, proteoglycan, and laminin. Collagen represents a key structural element of the ECM, and when its normal structures are broken, it can promote tumor growth. These ECM changes and the creation of the TM eventually contribute to invasion, tumor development, and metastasis^89^The relationship of sKGs between T2D and CRC is corroborated by prior studies, including CD44,^90^, ^91^, ^92^ THBS1,^93^, ^94^, ^95^ PLS3,^96^, ^97^, ^98^ PTK2,^99^, ^100^ CLDN5,^101^, ^102^ COL18A1,^103^, ^104^ CAV1,^105^, ^106^ and EFEMP1^107^, ^108^, ^109^ as displayed in Fig. 8A. Among the sKGs, the CD44 gene exists in inflammatory cells located in obese adipose tissue. In both humans and animals, CD44 most certainly contributes to the development of IR and inflammation in adipose tissue. Compared to other tissues, the expression level of CD44 was up-regulated in adipose tissue in T2D.^90^, ^91^ In KEGG pathway analysis, CD44 activates a number of signaling pathways, including the anti-apoptotic signaling mediated via mitogen-activated protein kinase (MAPK), phosphoinositide 3-kinase (PI3K)/AKT. The activation of these pathways is associated with tumor proliferation, migration, epithelial-mesenchymal transition (EMT), resistance to chemotherapeutic agents, and evasion of apoptosis.^110^ On the other hand, pancreatic islets in non-obese diabetic mice models have higher levels of CD44 and hyaluronan (HA) deposition, which makes pancreatic β cells more vulnerable to apoptosis cell death by autoimmune attack and, at least in part, reduces glucose-stimulated insulin release by blocking biosynthesis of insulin.^111^ In addition, CD44 is implicated in the onset of insulin resistance across multiple organs such as the pancreas, skeletal muscle and adipose tissue by the ways that are showed in the **Fig. S15**. CD44 gene is regulated through 2 transcriptional factors (i.e., EGR1 and SP1) and 2 post-transcriptional factors (hsa-miR-524-5p and hsa-miR-377) of sKGs. EGR1 expression is upregulated in CRC tumors in comparison to normal cells.^112^ In human CRC cells, EGR1 has been shown to stimulate tumor-cell proliferation and prevent apoptosis.^113^ EGR1 functions as a transcription factor that is capable of binding to target sequences and control the expression of numerous genes, such as CD44. The development of a medical therapy for insulin resistance and T2D may benefit greatly by targeting EGR1.^114^ On the other hand, many post-translational changes that are typically dysregulated in malignancies, such as acetylation, glycosylation, proteolytic processing, and phosphorylation, influence SP1 function. Elevated SP1 expression has been observed in a number of gastrointestinal malignancies, such as pancreatic, gastric, and CRC.^115^ Resistin, another adipokines linked to obesity and insulin resistance (Fig. 1**)**, has been demonstrated to be regulated by SP1.^116^ The microRNA hsa-miR-524-5p has been associated with colorectal cancer, primarily through its impact on gene expression that can influence cancer pathways.^117^ Conversely, hsa-miR-377 has also been recognized in other contexts for its potential relevance in cancer pathways, especially in regulating tumor growth and gene expression in colorectal cancer stroma.^118^ Research has shown that circulating microRNAs like hsa-miR-377 may impact glucose metabolism and play a role in the inflammatory pathways linked to diabetes.^119^ However, CD44 acts as a distinct insulin resistance biomarker and may serve as a candidate treatment target for T2D and CRC^90^, ^91^, ^92^.

Another key-gene THBS1 plays a significant role in how tumors grow and spread. When THBS1 expression is highly, tumors grow more slowly, show less angiogenesis, and metastasis happens less frequently. It was found that in those suffering from CRC, lower expression of THBS1 was negatively correlated with neoplastic recurrence and overall survival. According to the article showed that THBS1 is involved in metabolic dysregulation and adipose inflammation in T2D and obesity.^95^ THBS1 can modulate TGFβ-signaling pathway that plays a significant role into CRC. It also modulates VEGF signaling, PI3K/AKT Pathway that affecting tumor behavior and the microenvironment of CRC.^120^ On the other hand, accumulating evidence suggests that THBS1 is associated with an increased risk of developing type 2 diabetes (T2D).^121^ Besides it controls the activation of TGF-β1(Transforming growth factor-β1).^121^ TGF-β1 regulates various signaling pathways, i.e., By altering PI3K/Akt signaling, it induces pancreatic β-cell apoptosis, which leads to insulin resistance (IR). It may also contribute in Smad3 signal transduction, resulting in lower insulin sensitivity and ultimately metabolic capacity**.** THBS1 expression was higher in gastrocnemius muscle specimens from T2D patients with Peripheral artery disease (PAD) than in non-diabetic controls, which is clinically significant.^121^, ^122^ The sKG, THBS1 is regulated by 2 transcriptional factors (EGR1 and TP53) and 1 post-transcriptional regulators (has-miR-377). TP53 function has been linked to the development and growth of numerous metabolic disease, particularly diabetes and obesity.^123^ TP53 is linked to a higher risk of cancer in those with T2D.^124^ TP53 gene alterations are present in around half of all CRC cases; these mutations are more common in rectal and colon tumors.^125^, ^126^ Because of these characteristics, the THBS1 is a desirable target for CRC and T2D.^93^, ^94^, ^95^ Similarly, COL18A1, CLDN5, PTK2, CAV1, EFEMP1 and PLS3 genes are associated with T2D and CRC (Fig. 8A**)** as well as regulated by 3 transcriptional factors (EGR1, TP53 and SP1), 3 post-transcriptional factors (hsa-mir-377 and has-mir-539 and hsa-mir-135b) and also KEGG pathways, CCs, MFs, and BPs (Fig. 4**.** and Table 3**)**.

In our investigation, we found that four upregulated (CD44, COL18A1, PLS3, and PTK2) and four downregulated (THBS1, CAV1, EFEMP1, and CLDN5) KGs were *p-*value of < 0.001 at various CpG locations (**Table S8)**. Over the past decade, tumor immunotherapy has rapidly advanced as a central strategy in cancer treatment. To enhance understanding of the TM, research has increasingly focused on the infiltration of immune cells into tumor tissues.^127^ We examined the association between sKG expression levels and multiple immune-infiltrating cell types including dendritic cells, CD4^+^ T cells, B cells, neutrophils, CD8^+^ T cells, and macrophages in CRC and identified a significant relationship with CRC progression and development (**Fig. S2**). Thus, the relationship of sKGs between T2D and CRC get support by the literature review, independent databases and datasets. Molecular docking analysis was performed to explore sKG-guided therapeutic compounds with dual targeting potential for T2D and CRC. The four highest-ranked drugs (Irinotecan, Leucovorin calcium, Regorafenib, and Fenretinide) exhibited strong binding capabilities toward the sKGs-associated target proteins. These compounds were further confirmed for relevance to T2D and CRC based on literature evidence, shown in Fig. 8B. The drug compounds were computationally validated by an ADME/T, drug-likeness evaluation, DFT analysis, and Molecular Dynamic (MD) simulations. The suggested drugs exhibited good ADME/T properties, including high HIA levels ranging from 86.61% to 100%, adequate water solubility, and no carcinogenic effects. Ultimately, the stability of the highest-ranked four drugs (Irinotecan, leucovorin calcium, Regorafenib, and Fenretinide) was analyzed using 100 ns MD-based MM-PBSA simulations on the four leading proposed key-receptors (CD44, EFEMP1, TP53, and COL18A1), revealing their stable performance in alignment with physical principles. Fenretinide and Irinotecan^131^, ^132^ among the top-ranked four identified candidate drugs, were supported as common candidates for both T2D and CRC by separate studies on these diseases. Importantly, all proposed drugs are FDA approved for clinical use in the medication of other diseases. Following by the FDA, Irinotecan are used to treat patients with CRC and pancreatic cancer; Leucovorin calcium used to treat anemia and some types of cancer; Regorafenib used to treat CRC, digestive tract tumors, and liver cancer; Fenretinide used to treat prostate cancer, breast cancer whose accession ID is DB00762, DB00650, DB08896 and DB05076 respectively provided by the Drug Bank (DB). Fenretinide, a synthetic derivative of all-*trans*-retinoic acid, has shown promise as an anticancer agent, offering over other cancer treatments including CRC, as highlighted by extensive in vitro and in vivo studies, as well as chemoprevention clinical trials.^128^ Fenretinide alleviates obesity and IR in preclinical mouse models and is undergoing early clinical evaluation in obese individuals. It has the potential to be a new and safe medication for treating T2D, as well as for preventing and managing obesity and dyslipidemia.^129^, ^130^, ^133^ Irinotecan, a semisynthetic and water-soluble derivative of camptothecin, serves as a vital part of first- and second-line therapies for metastatic CRC.^131^, ^132^ Combination of irinotecan with metformin (widely used T2D drug) may be an effective therapeutic approach for CRC patients who are also suffering from T2D.^132^ According to the reference article, leucovorincalcium is used as an adjuvant chemotherapy treatment of CRC.^134^ Clinically, regorafenib, a potent multikinase inhibitor is approved for use in patients with metastatic colorectal cancer and gastrointestinal stromal tumors.^135^, ^136^ It should be noted here that the proposed T2D- and CR-causing sKGs required experimental validation in wet-lab and the proposed repurposable drugs as the common treatment for both diseases required clinical validation.

## 5 Conclusion

This study identified eight high ranked shared key genes (sKGs) (CD44, COL18A1, CLDN5, PLS3, PTK2, THBS1, CAV1, and EFEMP1) associated with both T2D and CRC through transcriptomic profile analysis, with the aim of exploring repurposable common drugs as effective therapeutic options for the concurrent management of both diseases. These sKGs effectively discriminate both T2D and CRC samples from their respective control groups. The differential expression profiles of the sKGs were additionally supported by independent-datasets obtained from the NCBI, TCGA, and GTEx databases. The sKGs regulatory network analysis identified three miRNAs (hsa-miR377, hsa-miR-539 and has-miR135b) and three TF proteins (EGR1, SP1 and TP53) that are linked to both diseases. Enrichment analysis of the sKGs identified numerous critical BPs, CCs, MFs, and signaling pathways implicated in the pathogenesis of both T2D and CRC. DNA-methylation profiling uncovered pivotal hypomethylated CpG sites that may play a role in promoting CRC development. Evaluation of sKG infiltration levels indicated a statistically significant association with various tumor-infiltrating immune cells in CRC. Finally, sKGs-guided top-ordered four drugs (Irinotecan, Leucovorin calcium, Regorafenib and Fenretinide) were proposed as common candidate drugs for both diseases following comprehensive molecular-docking, DFT, ADME/T, and MD-simulation analyses. Collectively, these findings suggest that this bioinformatics study may facilitate the development of effective therapeutic interventions for CRC coexisting with T2D. Aged patients often suffer from drug burden due to their multiple diseases, However, there has been little research on reducing such drug burden. This unique research protocol on reducing drug burden to the patients during the co-existence of T2D and CRC may become promising for other diseases also.

## 6 Availability of data

For this study, we retrieved datasets from the NCBI-GEO database, which are available free of charge at the link provided.

https://www.ncbi.nlm.nih.gov/geo/query/acc.cgi?acc=GSE18105.

https://www.ncbi.nlm.nih.gov/geo/query/acc.cgi?acc=GSE22598.

https://www.ncbi.nlm.nih.gov/geo/query/acc.cgi?acc=GSE29221.

https://www.ncbi.nlm.nih.gov/geo/query/acc.cgi?acc=GSE29226.

https://www.ncbi.nlm.nih.gov/geo/query/acc.cgi?acc=GSE20966.

https://www.ncbi.nlm.nih.gov/geo/query/acc.cgi?acc=GSE64998.

## CRediT authorship contribution statement

**Reaz Ahmmed:** Writing – review & editing, Writing – original draft, Visualization, Validation, Supervision, Software, Resources, Methodology, Investigation, Formal analysis, Data curation, Conceptualization. **Umme Samia Antu:** Writing – original draft, Visualization, Formal analysis. **Tasfia Noor:** Software, Methodology. **Md.Fahim Faysal:** Software, Methodology. **Arnob Sarker:** Visualization, Validation. **Sabkat Mahmud:** Visualization. **Alvira Ajadee:** Visualization. **Md.Nurul Haque Mollah:** Writing – review & editing, Supervision, Project administration, Methodology, Conceptualization.

## Declaration of Competing Interest

The authors declare that they have no known competing financial interests or personal relationships that could have appeared to influence the work reported in this paper.
