# Supplementary material for: Genetic underpinnings of type-2 diabetes (T2D) with colorectal cancer (CRC): In-silico discovery of common molecular signatures, pathogenetic processes and therapeutic candidates
Source: J Genet Eng Biotechnol. 2026 Mar 9;24(1):100667. doi: 10.1016/j.jgeb.2026.100667 (PMC12993328; doi:10.1016/j.jgeb.2026.100667)
Supplement: Supplementary Data 1 [file mmc1.docx]

**Supplementary File**

**Genetic underpinnings of type-2 diabetes (T2D) with colorectal cancer (CRC): *In-silico* discovery of common molecular signatures, pathogenetic processes and therapeutic candidates**

| **Supporting Items/Captions** | **Pages** |
| --- | --- |
| **Supplementary Figures** |  |
| **Figure S1.** (**A**) Expression patterns of sKGs with Boxplots by GTEx and TCGA database (**B**) Boxplots with Independent T2D Data | 03-04 |
| **Figure S2.** The scatter plot showed the relationship between the expression of sKGs and immune infiltrating levels of CD8+ T, CD4+ T cell, B cell, neutrophil, dendritic cell and macrophage in CRC. | 05 |
| **Figure S3.** The conformational angles (phi and psi) for each residue of beta-tubulin are shown in the Ramachandran plot. Red areas represent the preferred phi-psi angle conformations. The white region indicates an unfavorable phi-psi angle combination. | 6 |
| **Figure S4.** AutoDock Vina was employed to carry out the molecular docking analysis. Red indicates strong binding affinities between the target proteins and drug agents, whereas green represents weaker interactions. The image displays a score matrix, with the X-axis showing the top 30 drug agents (selected from a total of 307), and the Y-axis indicates the suggested receptors in an ordered sequence. | 6 |
| **Figure S5.** Top-ranked T2D-causing key genes (KGs) by protein-protein interaction of DEGs, where turquoise blue color indicated the KGs. | 7 |
| **Figure S6.** Top-ranked CRC-causing key genes (KGs) by protein-protein interaction of DEGs, where turquoise blue color indicated the KGs. | 7 |
| **Figure S7.** The visualized HOMO–LUMO frontier orbital surface patterns of (A) Irinotecan, (B) Leucovorin calcium, (C) Regorafenib, and (D) Fenretinide | 8 |
| **Figure S8.** After docking the pattern of HOMO and LUMO frontier molecular orbital surfaces of (A) Irinotecan, (B) Leucovorin calcium, (C) Regorafenib (D) Fenretinide | 9 |
| **Figure S9.** After MD- simulation the pattern of HOMO and LUMO frontier molecular orbital surfaces of (A) Irinotecan, (B) Leucovorin calcium, (C) Regorafenib (D) Fenretinide | 9 |
| **Figure S10.** Root mean square fluctuation (RMSF) plot of CD44-Irinotecan, EFEMP1-Leucovorincalcium, TP53-Regorafenib and COL18A1-Fenretinide. | 10 |
| **Figure S11.** Representation of the SASA study for the selected complex structure of CD44-Irinotecan, EFEMP1-Leucovorincalcium, TP53-Regorafenib and COL18A1-Fenretinide. | 10 |
| **Figure S12.** Representation of the Rg plot showing the changes observed in the conformational behavior of the all protein–ligand complex of CD44-Irinotecan, EFEMP1-Leucovorincalcium, TP53-Regorafenib and COL18A1-Fenretinide. | 11 |
| **Figure S13.** Ca-residue cross-correlation profiles for the **A.** Irinotecan complex, **B.** Leucovorincalcium complex, **C.** Regorafenib complex and **D.** Fenretinide complex. | 11 |
| **Figure S14.** Graphical representation of the PCA analysis of the top-ranked complexes of **A.** Irinotecan complex, **B.** Leucovorincalcium complex, **C.** Regorafenib complex and **D.** Fenretinide complex. | 12 |
| **Figure S15.** Role of CD44 in the signaling pathway of CRC & T2D | 12 |
| **Supplementary Table** |  |
| **Table S1.** Collection of T2D and CRC related candidate drugs from published articles and different online web-tools. | 13-17 |
| **Table S2.** List of upregulated and downregulated cDEGs between T2D and control samples based four microarray gene expression datasets (GSE2921, GSE29231 and GSE20966) | 17-21 |
| **Table S3.** List of upregulated and downregulated cDEGs between CRC and control samples based four microarray gene expression datasets (GSE18105, GSE22598 and TCGA database) | 21-26 |
| **Table S4.** List of upregulated and downregulated shared DEGs (sDEGs) between CRC and T2D | 26 |
| **Table S5.** Identification of shared DEGs (sDEGs) between T2D and CRC | 26 |
| **Table S6.** List of shared key genes (sKGs) from PPI network based on different topological measures | 26 |
| **Table S7.** The significant prognostic value of CpG in sKGs | 25-26 |
| **Table S8.** Docking scores (binding affinities, kcal/mol) between the proposed receptors and top ordered 30 candidate drugs (out of 307) | 26 |
| **Table S9.** Docking scores (binding affinities, kcal/mol) between the proposed receptors and T2D-control ligand | 26 |
| **Table S10.** Docking scores (binding affinities, kcal/mol) between the proposed receptors and CRC-control ligand | 28 |
| **Table S11.** Molecular docking scores (binding affinities, kcal/mol) with T2D causing genes | 28 |
| **Table S12.** Molecular docking scores (binding affinities, kcal/mol) with CRC causing genes | 28 |
| **Table S13.** Molecular docking scores (binding affinities, kcal/mol) with equal expressed genes or, unregulated genes that are not associated with T2D or CRC | 27 |
| **Table S14.** Some important docking results with the protein-ligand complexes. | 28 |
| **Table S15.** Before docking the physio-chemical descriptors, Frontier molecular orbitals and their reactivity descriptor analysis of top-ranked 4 compounds | 33 |
| **Table S16.** After docking the physio-chemical descriptors, Frontier molecular orbitals and their reactivity descriptor analysis of top-ranked 4 compounds | 31 |
| **Table S17.** After Molecular Dynamic (MD) simulations the physio-chemical descriptors, Frontier molecular orbitals and their reactivity descriptor analysis of top-ranked 4 compounds | 31 |

**Supplementary Figure**


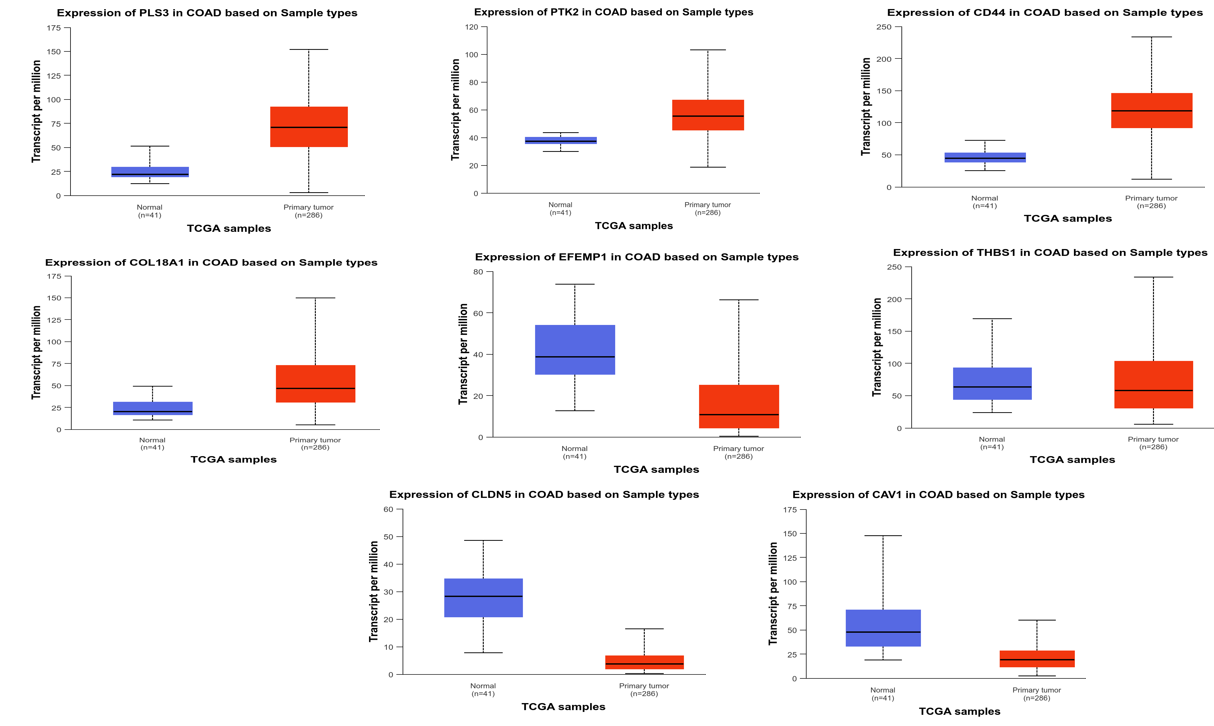


**A**


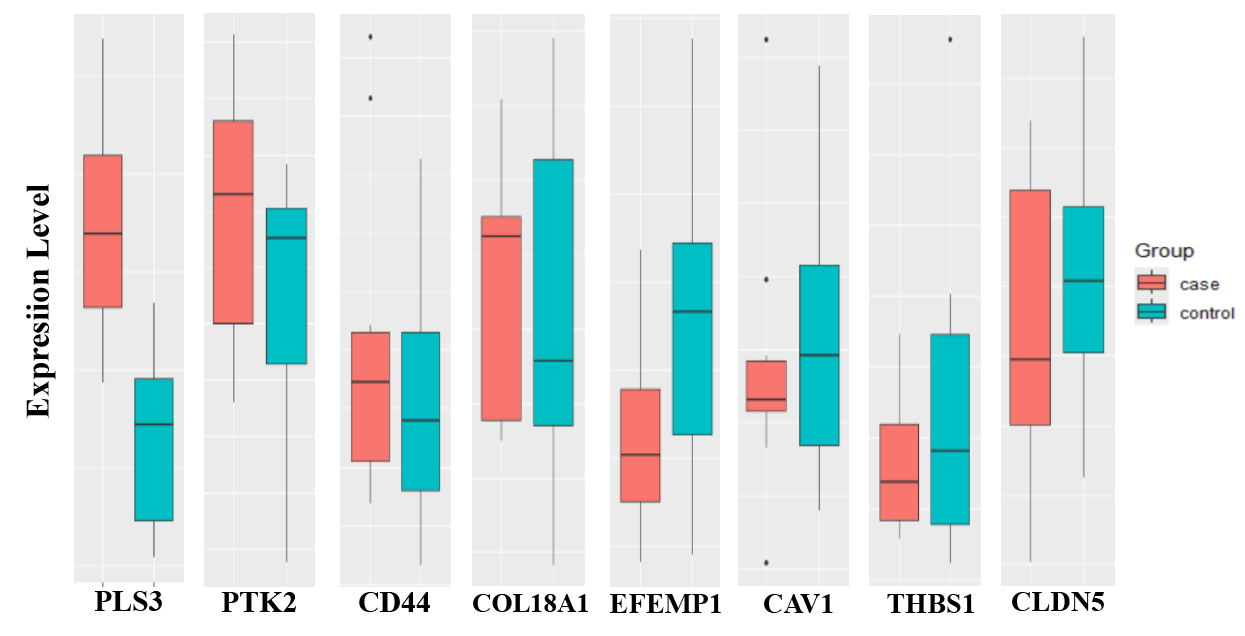


**B**

**Figure S1.** (A) Expression patterns of sKGs with Boxplots by GTEx and TCGA database (B) Boxplots with Independent T2D Data


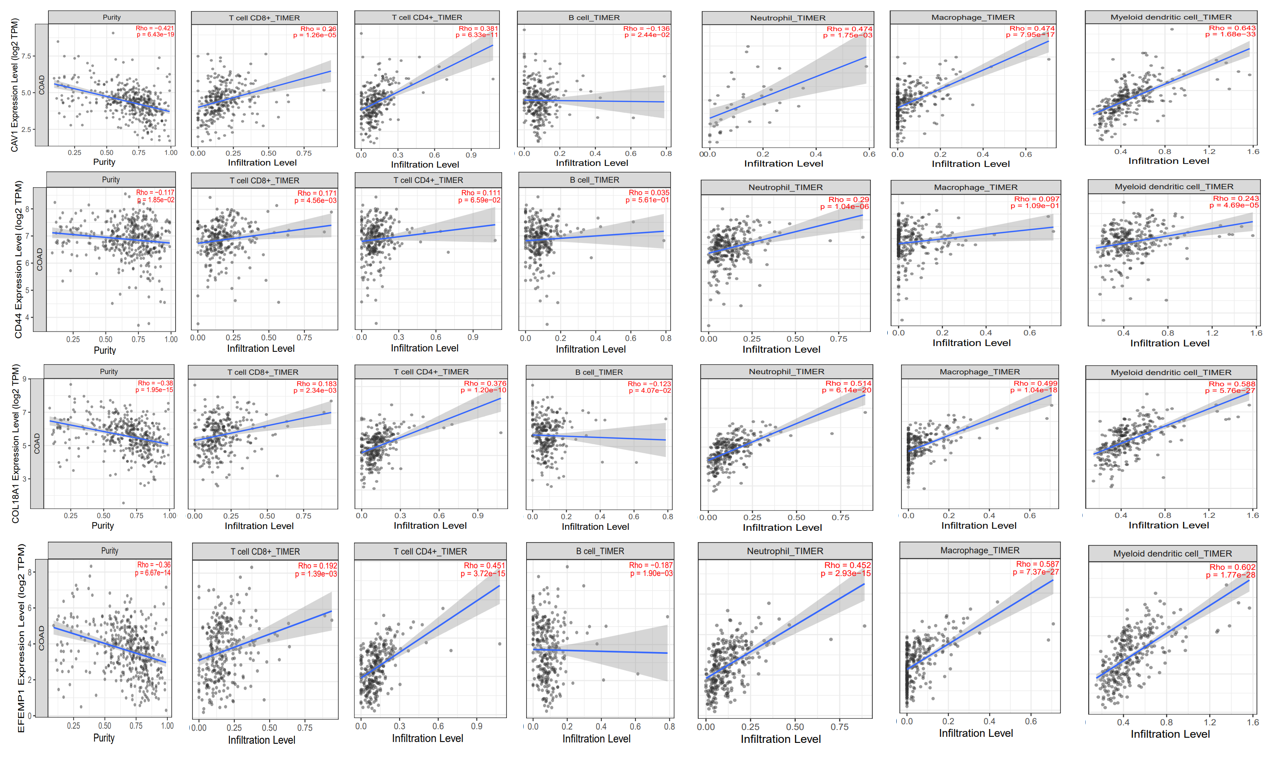

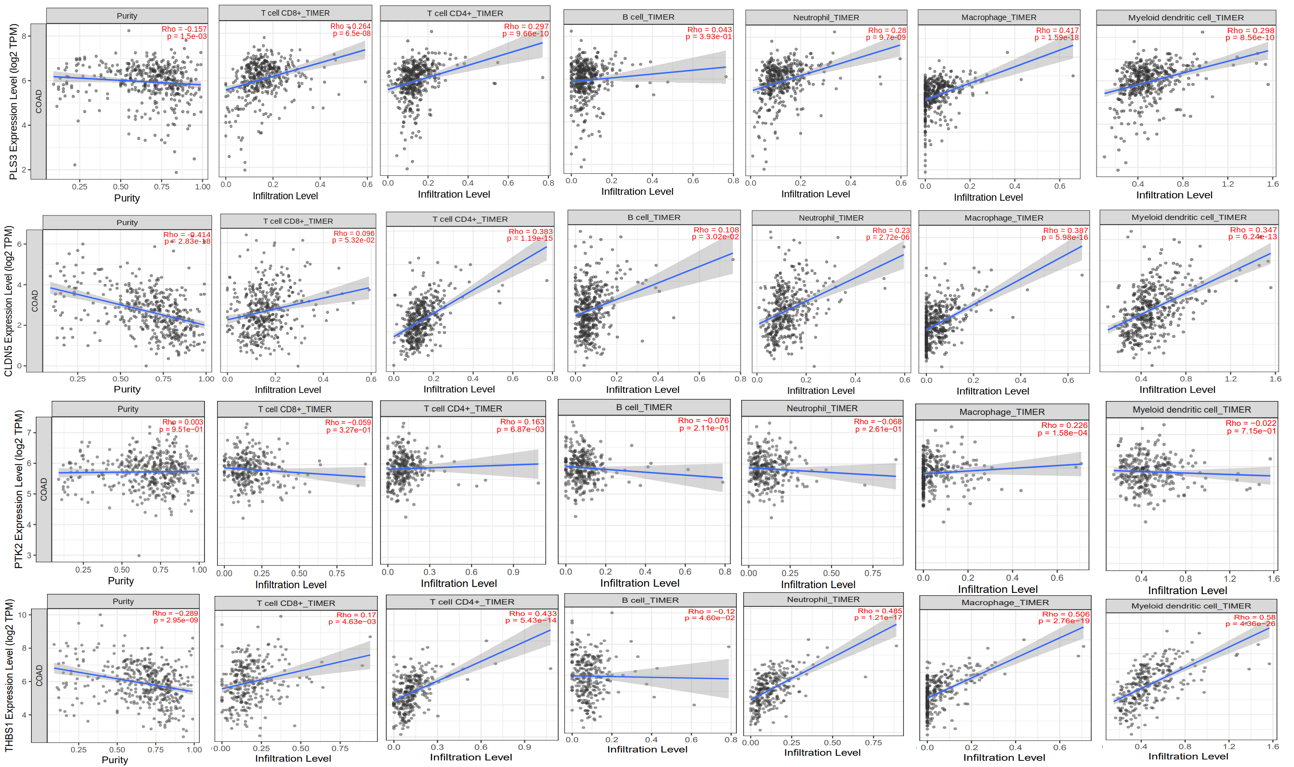


**Figure S2.** The scatter plot showed the relationship between the expression of sKGs and immune infiltrating levels of CD8+ T, CD4+ T cell, B cell, neutrophil, dendritic cell and macrophage in CRC.


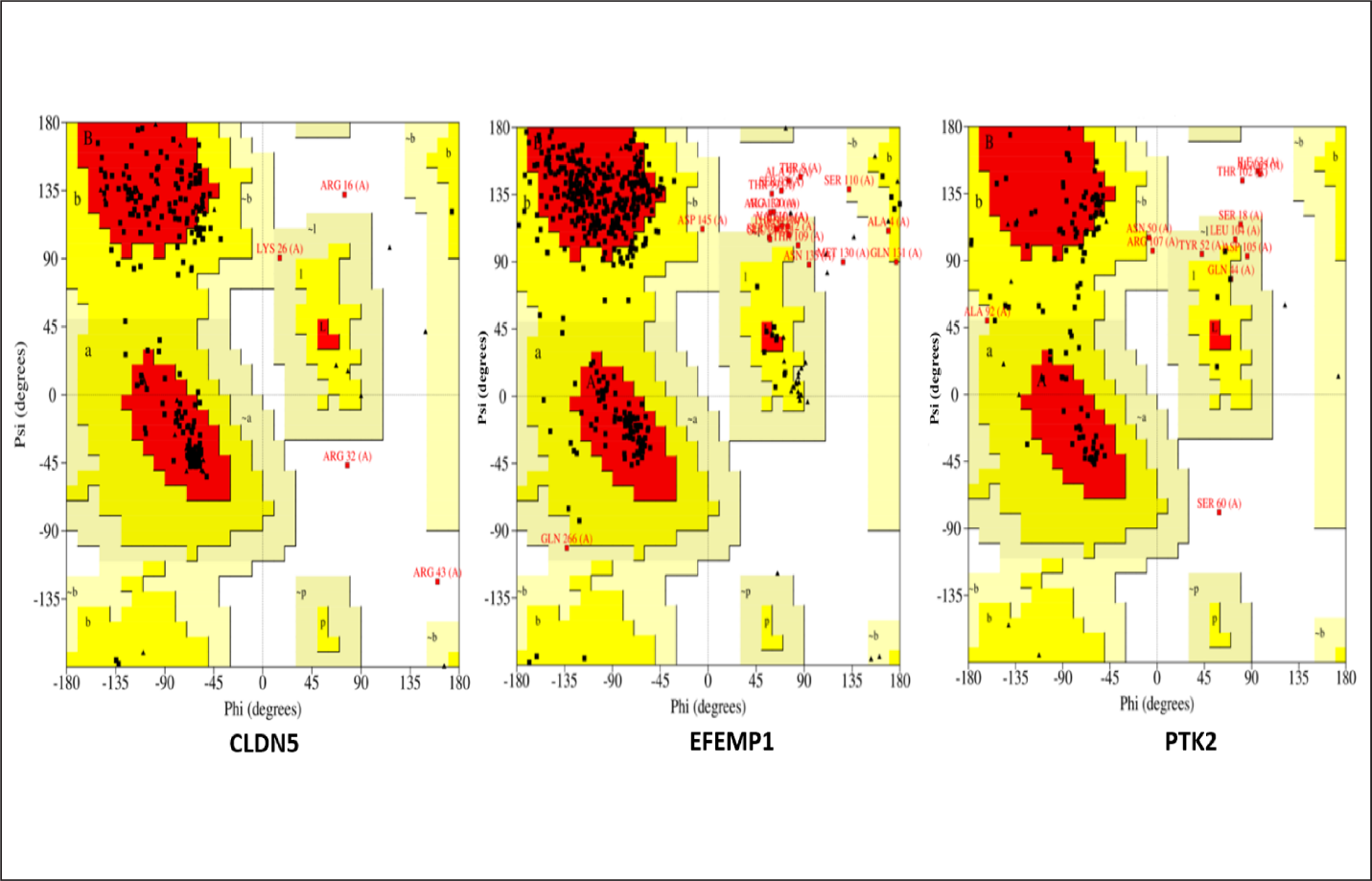


**Figure S3.** The conformational angles (phi and psi) for each residue of beta-tubulin are shown in the Ramachandran plot. Red areas represent the preferred phi-psi angle conformations. The white region indicates an unfavorable phi-psi angle combination.


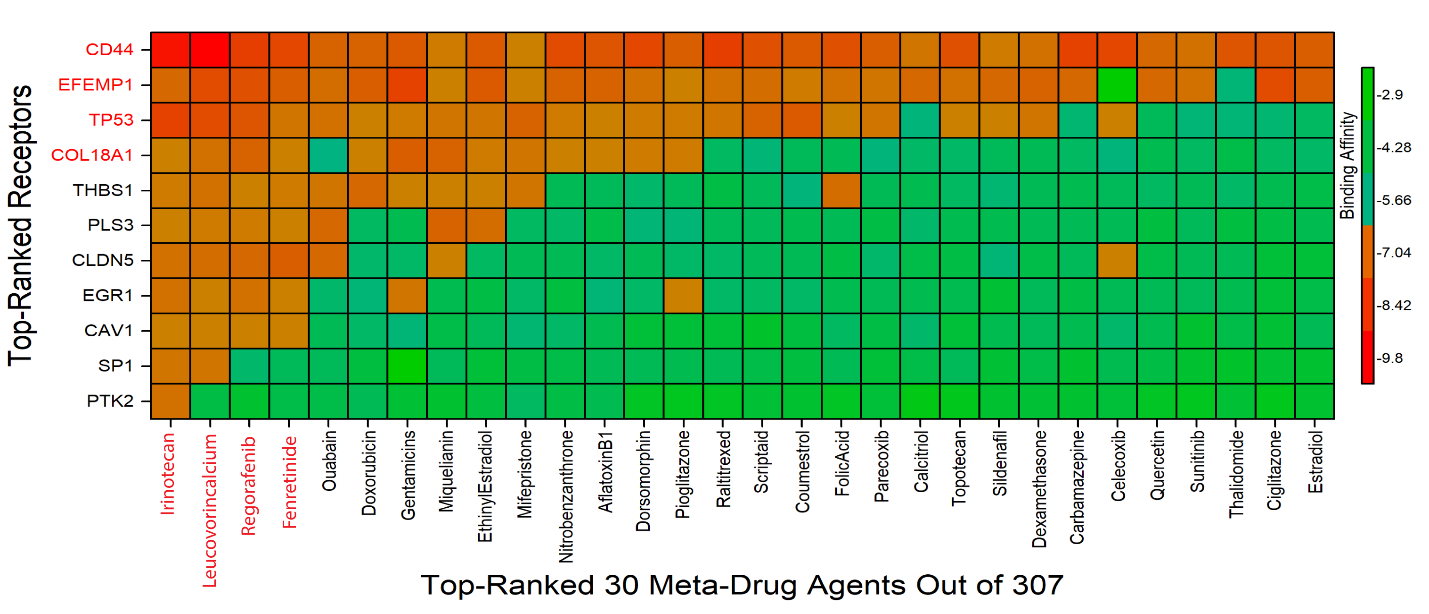


**Figure S4.** AutoDock Vina was employed to carry out the molecular docking analysis. Red indicates strong binding affinities between the target proteins and drug agents, whereas green represents weaker interactions. The image displays a score matrix, with the X-axis showing the top 30 drug agents (selected from a total of 307), and the Y-axis indicates the suggested receptors in an ordered sequence.


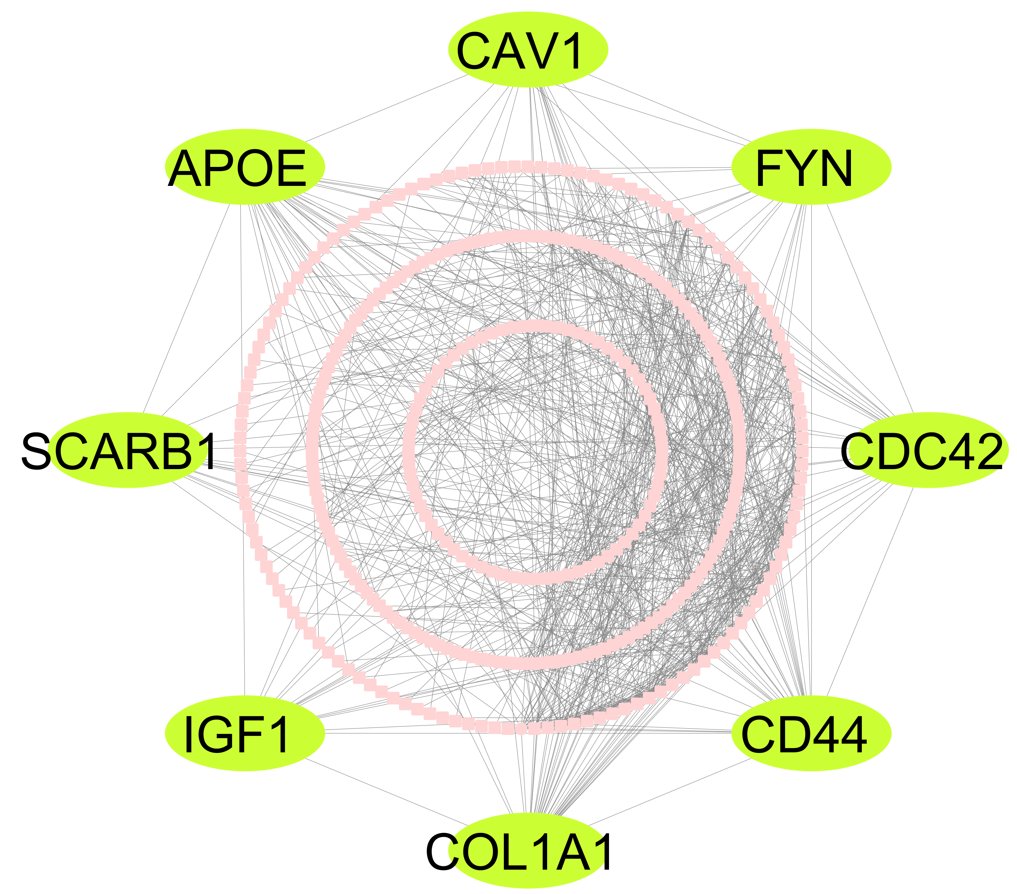


**Figure S5.** Top-ranked T2D-causing key genes (KGs) by protein-protein interaction of DEGs, where turquoise blue color indicated the KGs.


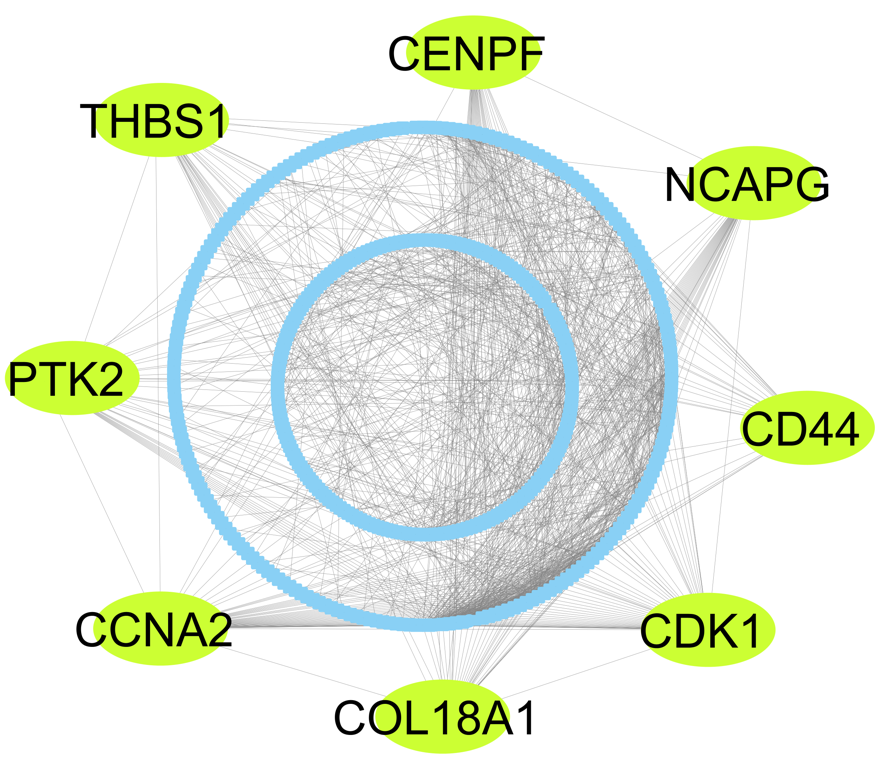


**Figure S6.** Top-ranked CRC-causing key genes (KGs) by protein-protein interaction of DEGs, where turquoise blue color indicated the KGs.


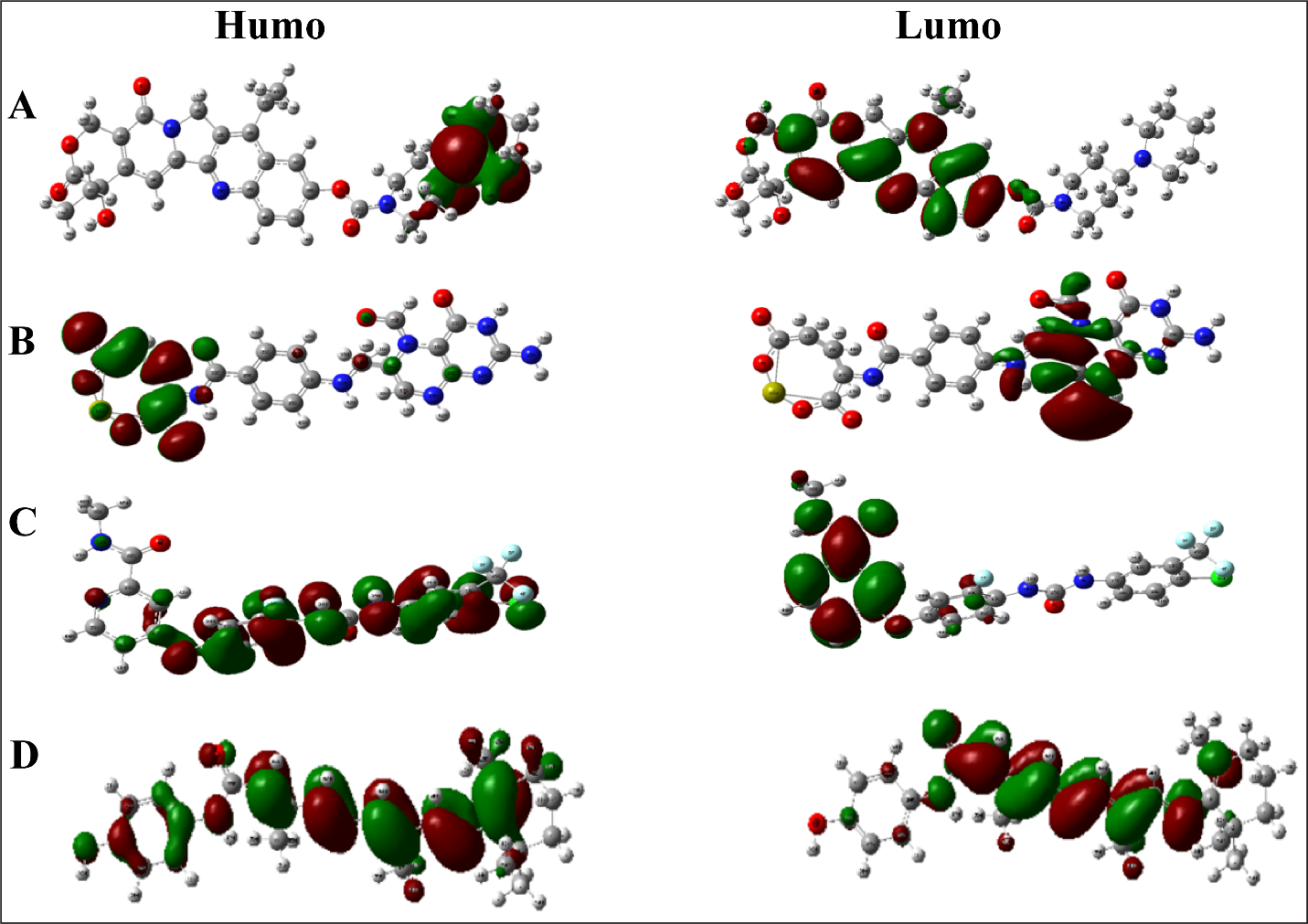


**Figure S7.** The visualized HOMO–LUMO frontier orbital surface patterns of (A) Irinotecan, (B) Leucovorin calcium, (C) Regorafenib, and (D) Fenretinide


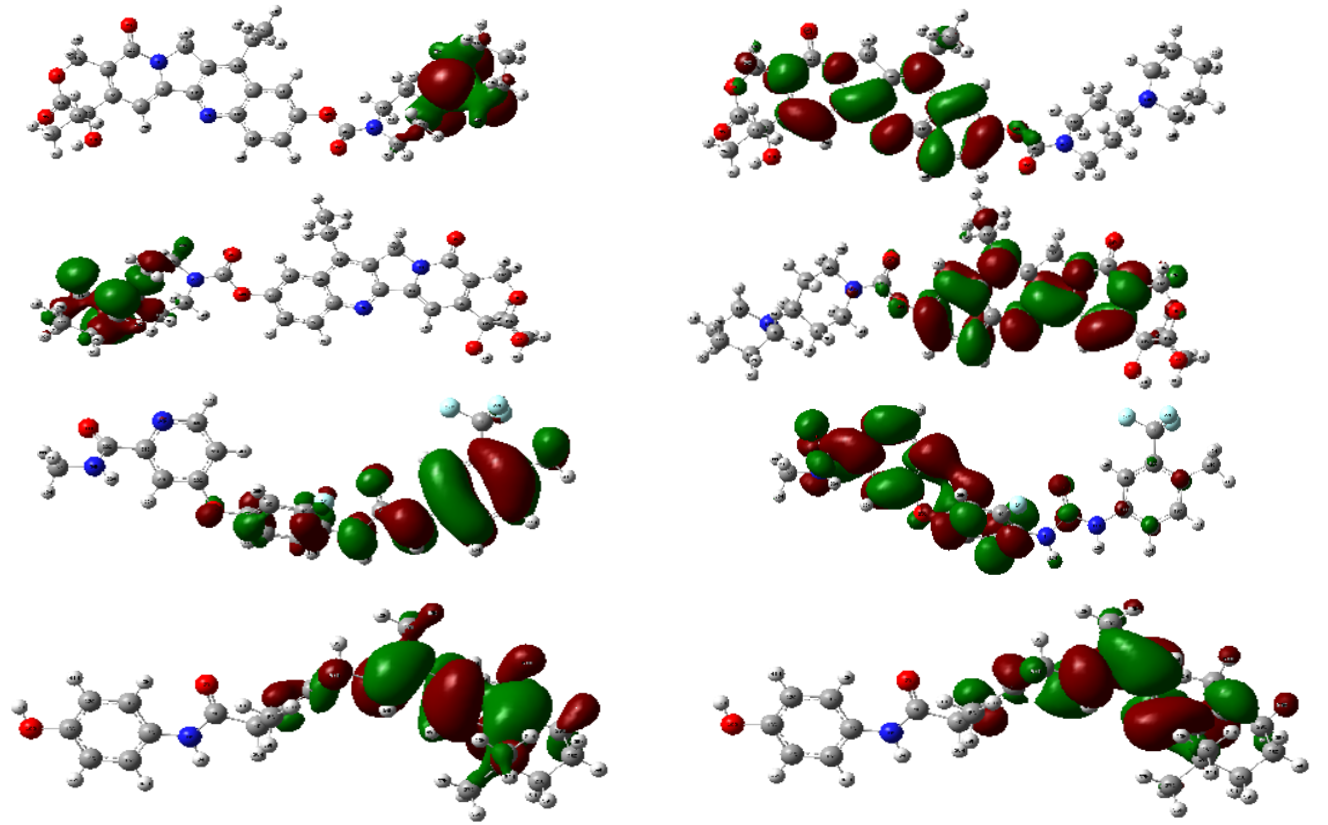


**Figure S8.** After docking the pattern of HOMO and LUMO frontier molecular orbital surfaces of (A) Irinotecan, (B) Leucovorin calcium, (C) Regorafenib (D) Fenretinide


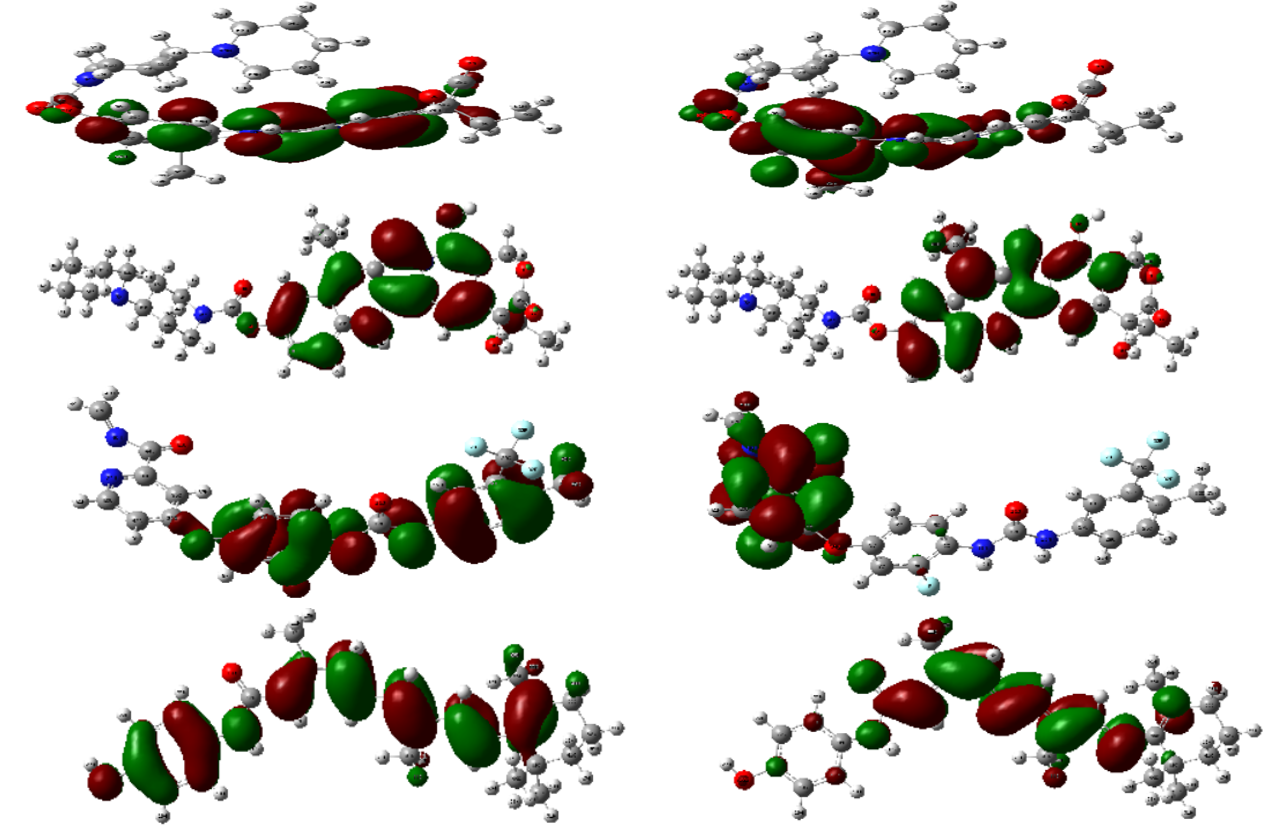


**Figure S9.** After MD- simulation the pattern of HOMO and LUMO frontier molecular orbital surfaces of (A) Irinotecan, (B) Leucovorin calcium, (C) Regorafenib (D) Fenretinide


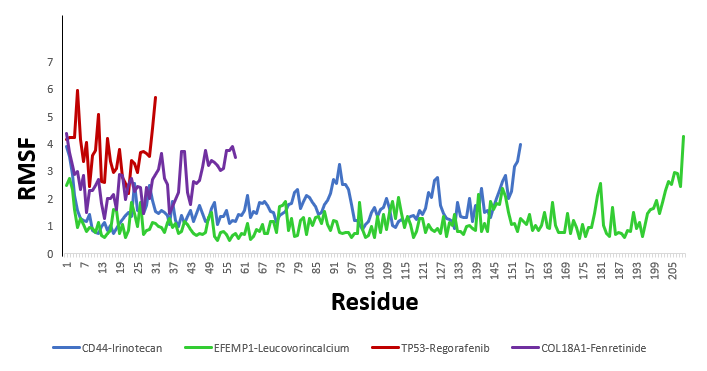


**Figure S10.** Root mean square fluctuation (RMSF) plot of CD44 - Irinotecan, EFEMP1-Leucovorincalcium, TP53-Regorafenib and COL18A1-Fenretinide.


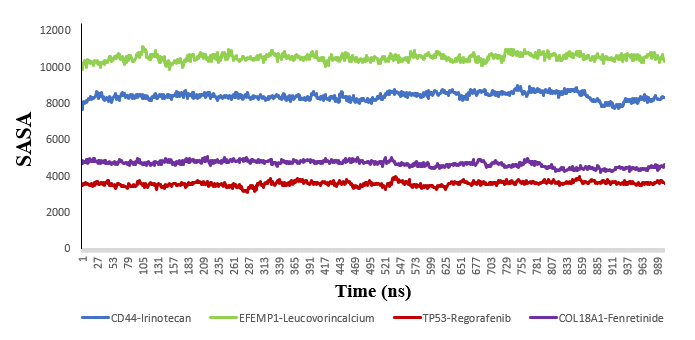


**Figure S11.** Representation of the SASA study for the selected complex structure of CD44-Irinotecan, EFEMP1-Leucovorincalcium, TP53-Regorafenib and COL18A1-Fenretinide.


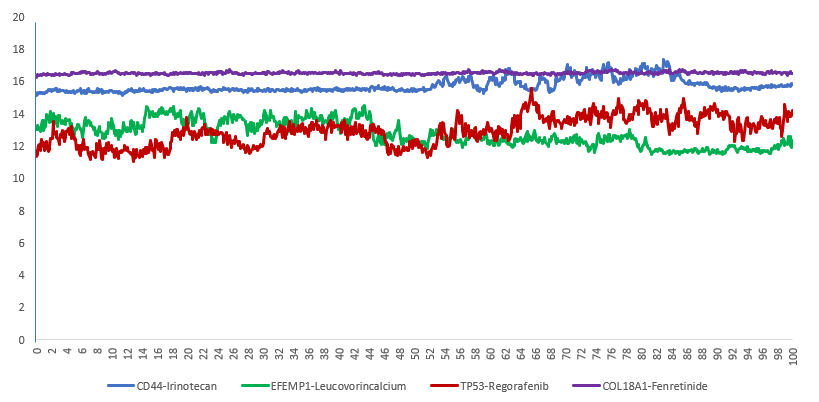


**Figure S12.**  Representation of the Rg plot showing the changes observed in the conformational behavior of the all protein–ligand complex of CD44-Irinotecan, EFEMP1-Leucovorincalcium, TP53-Regorafenib and COL18A1-Fenretinide.


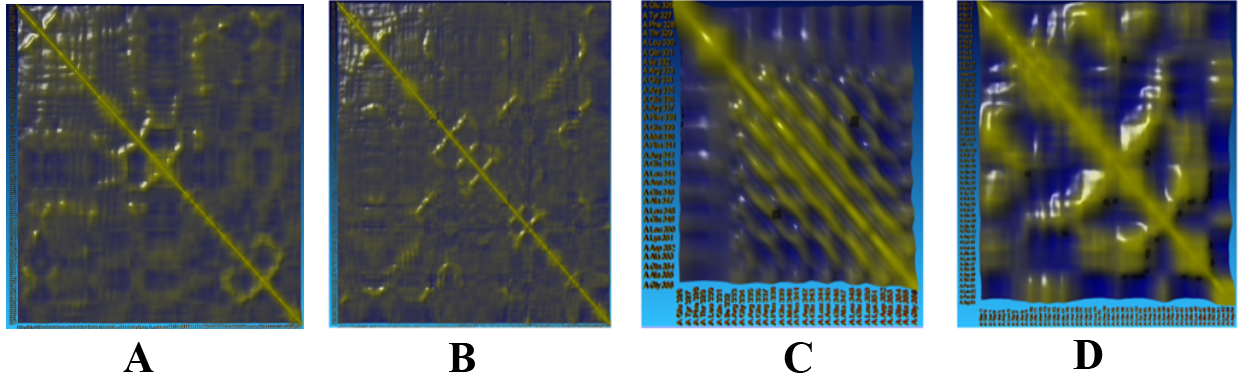


**Figure S13.** Ca-residue cross-correlation profiles for the **A.** CD44-Irinotecan complex, **B.** EFEMP1- Leucovorincalcium complex, **C.** TP53-Regorafenib complex and **D.** COL18A1-Fenretinide complex.


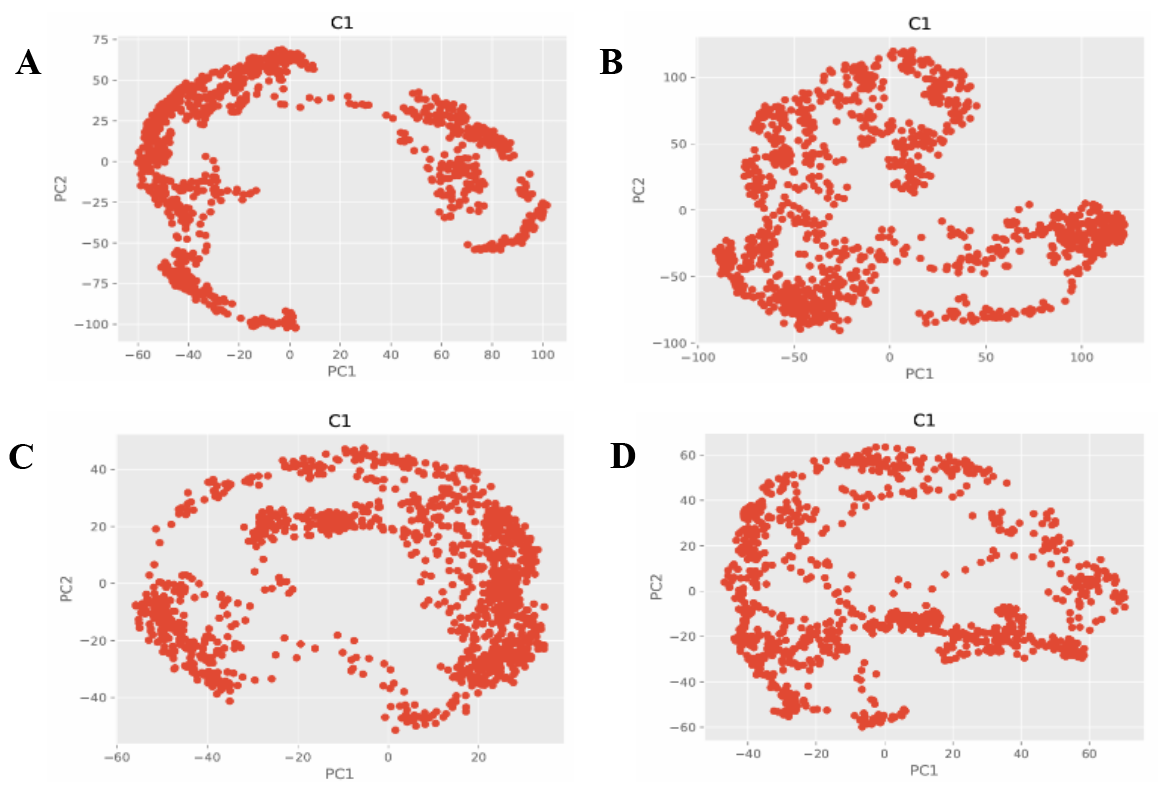


**Figure S14.** Graphical representation of the PCA analysis of the top-ranked complexes of **A.** CD44 - Irinotecan **B**. EFEMP1- Leucovorincalcium, **C.** TP53-Regorafenib and **D.** COL18A1-Fenretinide.


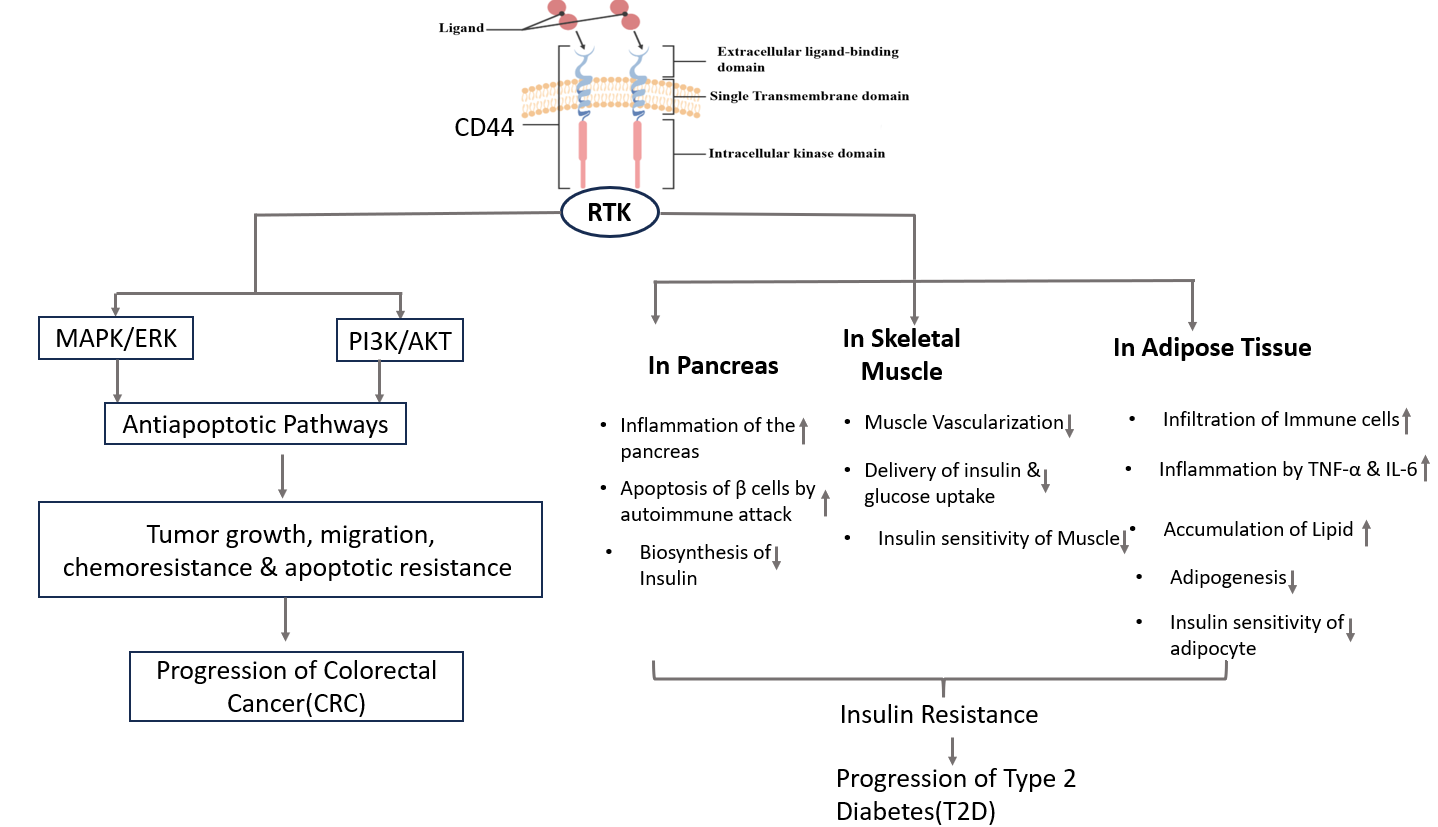


**Figure S15.** Role of CD44 in the signaling pathway of CRC & T2D

**Supplementary Tables**

**Table S1.** Collection of T2D and CRC related candidate drugs from published articles and different online web-tools.

| Disease type | Paper title with reference | Drug list |
| --- | --- | --- |
| **T2D** | [1,2] “ype 2 diabetes increases and metformin reduces total, colorectal, liver and pancreatic cancer incidences in Taiwanese: A representative population prospective cohort study of 800,000 individuals”  “Survival advantage observed with the use of metformin in patients with type II diabetes and colorectal cancer” | metformin |
| **T2D** | [3] Increased cancer-related mortality for patients with type 2 diabetes who use sulfonylureas or insulin | sulfonylureas |
| **T2D** | [4] New Drugs for Type 2 Diabetes Mellitus | Sulfonylureas, meglitinides, Metformin, Thiazolidinediones, Insulin |
| **T2D** | [5] Type II diabetes mellitus: a review on recent drug based therapeutics | Biguanides |
| **T2D** | [6] Pharmacology and therapeutic implications of current drugs for type 2 diabetes mellitus | biguanides, sulfonylureas and thiazolidinediones |
| **T2D** | [7] Oral agents for the treatment of type 2 diabetes mellitus: Pharmacology, toxicity, and treatment | Sulfonylureas, biguanides, α-glucosidase inhibitors, and thiazolidinediones |
| **T2D** | [8] Type 2 Diabetes and Oral Antihyperglycemic Drugs | Sulfonylureas, biguanides, α-glucosidase, meglitinides, DPP-4 inhibitors and thiazolidinediones |
| **CRC** | [9] Aspirin use for the primary prevention of cardiovascular disease and colorectal cancer | Aspirin |
|  | [10] Precision treatment in colorectal cancer: Now and the future | Panitumumab, cetuximab, Irinotecan, Oxaliplatin, Raltitrexed, Lonsurf, Bevacizumab, Ziv-aflibercept, Ramucirumab, Regorafenib |
| **CRC** | [11] Colorectal cancer: A comprehensive review based on the novel drug delivery systems approach and its management | 5- fluorouracil, cucumin, Doxorubicin, Paclitaxel, |
| **CRC** | [12] Molecular Targeted Drugs and Treatment of Colorectal Cancer: Recent Progress and Future Perspectives | bevacizumab, aflibercept, regorafenib, cetuximab, and panitumumab |
| **CRC** | [13] Colorectal cancer drug target prediction using ontology-based inference and network analysis | Bevacizumab, Capecitabine, Cetuximab, Fluorouracil, Irinotecan hydrochloride, Leucovorin calcium, Oxaliplatin, Panitumumab, Regorafenib, Aflibercept |
| **CRC** | [14] Value in colorectal cancer treatment: Where it is lacking, and why | Bevacizumab, Ziv-aflibercep |
| **CRC** | [15] Dostarlimab: The miracle drug for the treatment of colorectal cancer | **Dostarlimab** |
| **CRC** | [16] Small-molecule drugs of colorectal cancer: Current status and future directions | oxaliplatin, irinotecan, capecitabine and regorafenib |
| **CRC** | [17] New drugs for colorectal cancer - Mechanisms of action | 5-fluorouracil, Raltitrexed, Capecitabine, Irinotecan, Oxaliplatin |
| **CRC** | [18] Drug repurposing in oncology: Compounds, pathways, phenotypes and computational approaches for colorectal cancer | Zidovudine, Thalidomide, Sildenafil, Mifepristone, Methotrexate, Mecamylamine, Galantamine, Exenatide, Dexmecamylamine, Amantadine |
| **CRC** | [19] Oral drugs in the treatment of metastatic colorectal cancer | Bevacizumab, Cetuximab, Panitumumab, Aflibercept, Oxaliplatin, Irinotecan |
| **CRC** | [20] Promethazine inhibits proliferation and promotes apoptosis in colorectal cancer cells by suppressing the PI3K/AKT pathway | Promethazine |
| **CRC** | [21] Prevention and intervention trials for colorectal cancer | Erlotinib, Celecoxib, Aspirin |
| **CRC** | [22] Cyclo-oxygenase 2 inhibition in colorectal cancer therapy | Celecoxib, Rofecoxib, Etoricoxib, Parecoxib, Valdecoxib |
| **sKGs guided drug (T2D and CRC)** | [23] Enrichr-KG: bridging enrichment analysis across multiple libraries | \| Cytarabine, Fulvestrant ,Simvastatin, bisphenol F, Soman, fenamidone, Tamoxifen, Tretinoin, Phenylmercuric Acetate, Diethylnitrosamine, Diuron, Bleomycin, Temozolomide, Resveratrol, dorsomorphin, Fluorouracil, Coumestrol, Estradiol, Doxorubicin, Cisplatin, Streptozocin, Vorinostat, Folic Acid, Asbestos, Paraquat, Dexamethasone, Indomethacin, Genistein, Endosulfan, Ivermectin, Methotrexate, Calcitriol, Topotecan, Acetaminophen, Valproic Acid, Aflatoxin B1, bisphenol A, Benzo(a)pyrene, Sunitinib, Gentamicins, abrine, jinfukang, Cyclosporine, Fenretinide, propionaldehyde, pirinixic acid , Quercetin, methylselenic acid, Bucladesine, pyrachlostrobin, , Cycloheximide, Temozolomide, scriptaid, Pioglitazone, Decitabine, Carbamazepine, Aripiprazole, tyrphostin 25, Colchicine, ciglitazone \| \| --- \| |
| **sKGs guided drug (T2D and CRC)** | [24] δ-Aminolevulinic acid-induced fluorescence unmasks biological intratumoral heterogeneity within histologically homogeneous areas of malignant gliomas | Rifampin, Ifosfamide, Thioguanine, Ifosfamide, Doxorubicin, Hydroxyurea, Microcystin, Noscapine, Tacrine, Ouabain, Tiapride, Tenidap, Cisplatin, Doxepin, Ketamine |
| **CRC(DrugBank)** | [25] DrugBank: a knowledgebase for drugs, drug actions and drug targets | Yttrium Y-90, Aflibercept, Ga 68 PSMA-11, Cetuximab, Capecitabine, Oxymetholone, Vinorelbine, Erlotinib, Talimogene laherparepvec, Leuprolide, Progesterone, Mifamurtide, Vandetanib, Beta carotene, Azacitidine, Histrelin, Elotuzumab, Dabrafenib, Denileukin diftitox, Mogamulizumab, Thyrotropin alfa, Ixabepilone, Relugolix, Sacituzumab govitecan, Brexucabtagene autoleucel, Imiquimod, Clofarabine, Phenazopyridine, Cobimetinib, Nilutamide, Ibrutinib, Polatuzumab vedotin, Pentostatin, Pepsin, Mobocertinib, Plicamycin, Apalutamide, Tirbanibulin, Colchicine, rsPSMA Vaccine, Samarium (153Sm) lexidronam, Testolactone, Abemaciclib, Sargramostim, Ecamsule, Bendamustine, Avelumab, Thalidomide, Durvalumab, Ivosidenib, Estrone sulfate, Ruxolitinib, Selenious acid, Degarelix, Padeliporfin, Raloxifene, Niraparib, Dacomitinib, Lenograstim, Infigratinib, Dactinomycin |
| **T2D (DrugBank)** | [25] DrugBank: a knowledgebase for drugs, drug actions and drug targets | Bexagliflozin, Pentamidine, Albiglutide, Glipizide, Daridorexant,Insulin glargine,Insulin degludec,Glymidine,Triethylenetetramine,Acarbose, Pramlintide, Lanreotide, Neuropeptide Y, Diacerein, Tilarginine, Phentermine, Donislecel, Canagliflozin, Dexfenfluramine, Fenofibric acid, Acetylcarnitine, Oxytocin, Rosuvastatin, Ioxaglic acid, D-alpha-Tocopherol acetate, Inositol, Clozapine, Strontium ranelate, Metreleptin, Histrelin, Leuprolide, Nylidrin, Bezafibrate, Telmisartan, Gamolenic acid, Trandolapril, Omega-3-carboxylic acids, Lixisenatide, Exenatide, Sotalol, Sotagliflozin, Tolazamide, Chromium picolinate, Repaglinide, Saxagliptin, Ertugliflozin, Niacin, Insulin glulisine,Tirzepatide,Glimepiride, Miglitol, Acetohexamide, Tolbutamide, Linagliptin, Phenformin, Vildagliptin, Demeclocycline, Becaplermin, Tacrolimus, Alogliptin,Chlorpropamide, Benfotiamine,Mannitol busulfan, Troglitazone, Biguanide, Insulin aspart, Gliquidone,Botulinum toxin type B, Poliovirus type 3 antigen, Polyquaternium-10, Beremagene geperpavec, Apadamtase alfa, |

**Table S2.** List of upregulated and downregulated common DEGs between T2D and control samples based on microarray gene expression datasets GSE29221, GSE29226 and GSE20966

| **Downregulated cDEGs** | **Upregulated cDEGs** |
| --- | --- |
| ABLIM1, SORBS3, ABLIM1, DNASE2, PCYOX1, S100A6, AEBP1, THBS1, TNC, CCDC3, PDE5A, FTH1P3, PAMR1, GPNMB, THBS2, ANGPTL2, MGP, CTSK, SPHK2, COL1A1, NBL1, TNMD, DNALI1, STK39, ABCG1, FMOD, GBE1, STX2, FAM177A1, CACNB3, MGP, ACOT7, NBL1, ITGB5, SAC3D1, CTSC, JAM3, CTSC, GAS1, FBLN1, S100A4, CDC42, OLFML2B, AHNAK2, LTBP3, CD44, GALNT16, TUSC1, CHPF, SFRP2, IFNAR2, CPXM2, AGBL5, TRIP4, CD151, CD44, CTSC, RECK, LPAR1, S100A13, ZFPM1, BGN, FBLN1, SLC7A1, MXRA7, RFX1, RPPH1, S100A4, ZNF503, SLC22A17, MSRA, MAOA, WISP2, PELI2,CRIM1, ZDHHC1, DDR2, RNASEL, CXCL14, PORCN, CRTAP, SNAPC2, PTMS, DKK3, OSBPL5, ABI3BP, GPNMB, ACTG2, NDNF, HTRA1, ITPK1, CXCR5, TMEM98, SPARC, SEPSECS, ISLR2, TIMP2, LHFP, CFAP20, RERG, NAALAD2, CHST7, RDH11, SHPK, TMEM18, SLC12A9, TAGLN2, ITGA11, OLFML1, SPOCK2, DCLK1, FRMD6, CRIP1, CNIH1, PLAT, ZNF500, MBOAT1, ADAL, CHST14, OR11L1, F8A1, LHPP, PALM, CD151, SALL2, APOC1, C14orf180, IFT57, PLA2G16, AXL, RPRML, LTBR, SLC37A3, CFAP69, SYT11, IGFBP3, TOX2, DKK3, RAB34, ST6GALNAC6, CDC42EP5, ROBO3, CCDC89, ZNF219, IGFBP4, SLFN5, MARK1, MOB3B, USP3, GLIPR2, SNORD13, ATN1, TRIM47, GRK5, MAGEE1, C1QTNF5, DPYSL2, GBP2, ZNF415, TP53I13, PLS3, S100A10, PKD2, SLIT3, MSX1, COLEC12, TAGLN2, DOK4, DGKQ, MEIS3P1, SH3BP4, SMIM14, CALD1, ITGB5, VHL, ZNF182, WDR90, ITGB5, GTF2E2, SCRN1, LRRCC1, MYLK, TMEM54, RBMS3, FAM188B, IGFLR1, CFH, ECM2, SH3PXD2B, ANTXR1, TCEAL9, CYBRD1, CD34, C3orf70, COL18A1, CFH, VASN, FAM13A, ZNF467, MAN1C1, MYOF, BASP1, CCDC102A, FBLN2, METTL21B, LXN, ODF2, DOCK1, KCNJ8, PCOLCE2, CALD1, CYP2U1, TRADD, COL14A1, PCSK5, CRTAP, CORO2A, ZSWIM6, SLC44A1, TYSND1, COL1A2, NLGN2, CD34, C20orf27, SLC2A10, IGFBP3, TSPAN15, ASAP2, LMNA, CFH, MON1B, COL1A2, GAS7, RAI2, CD99, KIF3B, IGFBP6, CEP135, CAMK1D, CLEC3B, TMEM238, FXYD5 , RTN1, TCFL5, CAMK2N1, RASSF7, CD81, PMEPA1, FURIN, AP3S1, SVEP1, GPM6B, RARRES1, CYBRD1, ALDH1A2, PLOD3, SCUBE2, ECM2, KDELR3, CYGB, S100A10, BEX3, NACAD, MOK, SLC25A43, RAB31, PLS3, PPFIBP2, B3GALT6, LRFN3, AKAP10, PC, TMEM184B, PGAM1, IL33, COL3A1, CARD10, LTBP4, OSBPL5, SOWAHC, C8orf82, SLC43A2, SF3A2, FAM107B, PDIA5, BGLAP, MSN, COL6A2, HAUS4 , IRF2BPL, VEGFB, TBC1D9B, WDR19, GIMAP7, RAB7B, MYOF, CTSZ, MAN2B1, FTH1, PMEPA1, C17orf62, RNASEL, FASN, FAM110D, CD302, LAMA5, VAT1, NECTIN3, MAPK7, C4orf3, LMF2 KIF20B, CGNL1, BCKDHB, TOM1L2, LOC401052, NDRG1, ENO2, EDN1, AGRN, PROS1, FAAP20, GRAMD3, LINC01089, PLCXD1, BRAF, PABPC1, PRELID1, FER, COL16A1, DUT, WDR54, RAB13, AQP1, PLEKHA4, SGK223, NICN1, CHN1, SPTBN1, HSD17B11, TCIRG1, PXDN, FUT4, PNPLA2, RASSF5, FAIM, TMED3, GZMH, ARHGAP15, DYNC2H1, SULF1, PTPRA, ODF2L, TTC8 SERPINI1, GPIHBP1, SREBF1, COL12A1, SERPING1, MPZL2 , CCT6B, CSPG4, GNG10, CIDEA, ADGRA2, ALG14, MFAP4, MXRA7, ANXA2, ARSD, PDGFRB, MAL, CREB5, TRIM4, NDST1, SREBF1, LGALS8, PMM1, AKR1C2, FAM122A, MYADM, ADD3, TGFBR2 , ENPP2, AHNAK, TMEM259, SRPX2, BTG1, RNASE4, CLEC2D, ATP11B, CDC42SE1 ,ACTN1, TRIM38, CPQ, MRI1,PHOSPHO2, TMEM8B, FAXDC2, PGRMC1, TYMP, ALDH2, FYN, MAPK10, TRANK1, ANKRD13D, CDO1, PGLS, ANXA1, CARHSP1, PALM, NTN4, ALG9, ATP7B, CCND2, CLDN23, RAB32, FSTL1, LETMD1, CNN3, KLF8, FGFRL1, NDN, GNG11, GSN, FAM122B, TXNDC16, NTRK2, PARP11, LYL1 CD2, ANTXR2, C2CD2, RAP1GDS1, COL6A2, CD9, MOSPD2, MRI1, SLIT3, CCND2, HOXB2, CDC14B, ADAM15, VIM, CCL19, FAM129B, HPS3, GPM6B, CRIP2, TRPC1, CYB5A, COL5A2, CAMK1D, FAM213A, CBX6, IGFBP7, SNCG, LDOC1, ACLY, SLC25A16, FAM13A, CLEC11A, RAB5C ADCY3, IDUA DPT, CTNNBIP1, CLN5, ATP2B4, STX2 , RBMS3, MIS18BP1, ANXA2, EFEMP1, COL5A1, IGF1, CNRIP1, GLI3, CTIF, TGFBR3, SCARA5, MAP3K11, EBF1, CD24, ATP2B4, FBLN2, CKS1B, CHSY3, KLHL5, HES4, SDCBP, RFTN1, STRN4, CDK6 , RNF26, SCD, TAOK2, PRR5, PTCH1, MAST3, IGFBP2, PFKL, ENPP2, ALDOC, MALL, PON3, DGAT1, ING3, TXNDC5, C19orf25, FBLN1, LYRM4, VIPR1, RASA3, ENTPD1, CBR3, PLOD2, ACCS, PCDH19, DYNC2LI1, XXYLT1, TMEM243, ITGB4, ADAP1, KANK2, CADM3, NR2C2AP, LPCAT1, GRIPAP1, THRSP, LRFN4, PLSCR4, RHOJ, DLC1, ADM5, TPM4, HSPB11, SARM1, PHYKPL, SOX18, FGL2, OLFML3, FBXW7, CKS1B, RAB5C, TP53I3, BNIP3L, VIM, MTMR11, CIDEC, MCUB, VEGFB, PLVAP, TSKU , WNT11, NSMF, PQLC3, DCN, PHLDB1, LUM COL4A2, CCL5, SOGA1, AQP1, PECR, SNTB2, EPB41L2, TWIST1, CETP, ACTN4 ALG8 , EPB41L4A-AS2, DPP7, FXYD5, RASAL2, AK4, ZDHHC12, HOOK2, TGFBR2, SUSD2, VWA1, PC, DHDDS, MYH10, CLIC1, AACS CMIP, LFNG, PPM1M, AK4, KANK3, CBX1, CAMK1D, ENPP1, MAP1B, KMT5B, VEGFC, MAP4K1, LAPTM5, C5, NEK11, RENBP, SIPA1, PITPNC1, TSPAN4, AGPAT2, PACS1, TMEM14A, NADSYN1, CACNA1H, MGLL, OAF, PLPP1, ARID3A, AGPAT2, HK2, UST, ADA, CLIC6, KLHL5, ANG, NUDT18, EML3 , TRIP6 CPQ,PLXNA3, PLAC9, SH3BGRL, DLG4, TYSND1, MECOM, GNG12 , CD151, ZNF580, PTGR1, EFNA1, CD3D, APOE, SESTD1, CAMK1, ZNF540, HIC1, ARF3, LIPA, MYL9, CELF2, CLEC1A, MCOLN3, STAT5A, FAM114A1, CASP2, FSCN1 , ACP6 , ANXA2P1, FGFRL1, WFS1, ACKR1, AGPAT2, ENG, LIPE, STK10, ACLY, DAAM1, LEP, TNFSF10, RILPL2, KCTD12, TAX1BP3, TSHZ2, ITIH5, BTD, STK32B, ZNF385D, FAM84B, TIMP3, PTGR1, GPC3, SAP30BP, FGD3, PRPH, MXRA5, CMTM3, CAV2, UNC93B1, STAT6, REXO1, OS9, SEC23B, TNFAIP8L1, ANXA2, CAV1, KCTD11, MDK, ID3, RBPMS2, SYTL4, IFT52, SH3BGRL2, PPP1R1B, TSPAN4, TANC1, NUDT5, SLC50A1, SHISA5, AGBL5, CXCR4, RASAL3, CABLES1, GCA, LIMA1, BEX3, TCF3, ECSCR, G6PD, PGBD1, CADPS2, ARHGEF40, GUCY1B3, TMEM50A, CDKN2C, HACD3, TMX3, GABRE, PDDC1, MMP28, RNF135, PRCP , FGD5 , COX4I2, NCOA7, PIGO, ELL2, GPER1, EDNRA, ACOT7, ANG, LHX6, IFI16, GPAM, ATOH8, AIFM2, SRI, CLDN5, MGST1, MGLL, CAV2, GYG2, FLNB, ADAM15, PCOLCE, FYN, N4BP2, TOP1MT, ZFP36L2, TUBB6, ZSCAN16, MLXIPL, SAT1, CORO1B, IDH1, SLC25A1, APLNR, ITPR1, HOXB7, AKAP12, ALDH18A1, SCARB1, PEMT, CYP26B1, DCUN1D3, LBR, CTF1, APMAP, ADIRF, GAS2L1, CORO1C, VWA1, ZNF682, ORMDL3, PPP1R18, HNRNPA3, TNFRSF14, XPR1, ZNF763, RUNX3, GIMAP8, SDCBP, DMWD, CD247, MAPK11, SNORA25, B3GALT4, ELOVL1, ITGB1BP1, TRAM2, JAM2, RRBP1, TMEM37, WASF2, FADS1, TNFRSF21, STC1, TTPAL, SNURF, DOCK10, INF2, EMP1, FAM184A, GJC2 | ENC1, KCNN2 , HBEGF, FRMPD1, AMOT, ZHX2, ARAP2, RRP12, LOC100288798, PDLIM5, HADHB, CXorf57, SLC25A25, IER5, NR4A3, SMYD2, VEGFA, PANK1, ATXN7L2, SLC25A25, PUDP, CACNA1B, NDUFB1, POPDC2, SLPI, FGGY, TBC1D3F, COL7A1, MYBPH, SLC25A34, DDIT4L, FHOD3 GOT1, HUS1B, CASQ2, LRP4, HSPA1A, ATP5G1, ADCY2, RYR3, GADD45G, PANK1, JPH2, USP54, KIF1B, CKMT2, ALPK2, HIST1H1C, PPARGC1A, MYH3, SMARCD3, TRIM69, IP6K3, CIART, LRP12, HCFC1R1, PROX1, IGSF11, SCN3B, SHISA2, CLTCL1, RORC, MYH7B, CLTCL1, THAP4, PCNT, LGALSL, USMG5, CFAP61, SLC16A10, SNAR-A1, TUBA8 HES1, CA2, FAM46C, HCFC1R1, ABCC12, PRKAB2, N4BP2L2, DNAJB5, RPS23, MN1, GOT2, BZW2, RBM20, ALG1L, MAP6D1, TPM3P9, CPT1B, PTK2, MN1, DENND2C, CIPC, SLC25A4, PPP1R3C, CA2, SLC38A1, SLC25A30, SLC29A2, PLCD4, CA3, SLC25A4, RORC, LOC105377348, LDB3, TGFB3, PTP4A3, SLC20A2, TPI1P2, KCNT1, MYH1, MED14, CLIC5, SIX4, ADPRHL1, GSTM1, GFRA4, RNF123, TUBA4A, MYL3, ATP1B1, FAM78B, TRIM54, XPO4, GMPR, MYOM1, RAPSN, FKBP3, CA3, XK, KCNB1, RXRG, KLKB1, CCDC28B, UGP2, COLGALT2, PTP4A3, FLNC, AGL, RAPSN, SRL, CIPC, CYB5R1, PTP4A3, IDH2, TPM3, PLA2G4C, FKBP3, IL17D, EYA4, YBX3, HIST1H2BD, HIST1H2BD, CAND2, ATP2A2, DNAJC12, NEXN, FITM1, DMD, DTNA, RAMP1, AGL, BVES, GAMT, DTNA, TNNC1, CCDC28B, CAP2, HRC, DCAF5, PRR16, LINC00116, ACTN2, C19orf18, TMOD4, MYL2, LGI1, MYOT, MURC, ACTA1, PPP2R3A |

**Table S3.** List of upregulated and downregulated common DEGs between CRC and control samples based on microarray gene expression datasets (GSE18105, GSE22598)

| **Downregulated cDEGs** | **Upregulated cDEGs** |
| --- | --- |
| CPM,SPIB,MMP28,ADH1B,ST6GALNAC6,CA7,CNNM2,GCNT2,SST,SMPD1,ABCA8,BEST4,SCARA5,SCNN1B,BEST2,GUCA2B,INSL5,ACKR1,FAM107A,TMEM100,IL6R,SCNN1G,ZZEF1,CLU,PYY,SFRP1,SLC25A34,SLC4A4,LDHD,KLF4,FBLIM1,GUCA2A,C2orf88,CLEC3B,ALPI,GLTP,MT1M,FAM214B,PADI2,CHST5,CDHR5,OGN,GNA11,CHRDL1,CCL23,SLC22A18AS,TMEM72,CA12,CCL14,IGH,SLC9A1,PPARD,CXCL12,RDH5,PCBP1AS1,C11orf86,ZG16,NEDD4L,CACFD1,CDKN2BAS1,TP53INP2,C10orf54,GBA2,ENTPD5,APOBR,SCIN,PACSIN2,,UNC5C,PLCD1,CLDN8,GCG,KAZALD1,CPNE5,PTGDS,DISP2,LOC647115,TNXA,DHDDS,CLDN23,VSIG2,BEX1,PER1,CA1,IGSF9,SLC30A10,PLEC,ACADS,HIGD1A,TSPAN3,APBA1,SIRT6,PHYKPL,B3GALT5,SLC25A23,B3GALT4,GIPC1,NAP1L2,PTPRH,FAM189A1,TMEM61,FAM83E,PDZD3,KLF2,ARHGDIA,USP2,WSCD1,HRASLS2,C2orf40,CIDEC,PDE9A,TMPRSS2,EHBP1L1,ZNF575,CHGA,AAK1,FZD5,GSN,CIDEB,TPSAB1,B3GALT5,AS1,TPSB2,CA4,ACOT11,GDPD3,ARHGEF18,ATG4D,NAA60,TRANK1,JAM2,SPINK5,ZBTB7C,PDE7B,SEMA6D,RHOF,GREM2,RNF125,C1orf210,SCGN,TNIP1,CCDC107,MAMDC2,CLCA4,PHGR1,MBNL1,AS1,C7,B3GNT7,LETM1,BTNL3,SPDEF,AK1,SMIM5,FAM63A,MXD1,ABCG2,CLDN5,PLPP1,FBXO32,PINK1,KLB,NMRAL1P1,TTLL6,TPSAB1,STK40,CILP,CFLAR,EPHA10,MIER3,SLC44A4,ARHGAP44,MYO1C,CAPN5,RHBDL2,RILP,CD177,MALL,FAM160A2,EPS8L1,FOSL2,SMIM6,TECPR1,EMP1,RAB4B,ATP2A3,C6orf136,SMPD3,SLCO2A1,PDCD4,DPT,JUND,P2RY2,CCL19,SCAMP2,TEX11,FBXW5,MEOX1,HRCT1,HECTD3,ATP13A4,ITPKA,RIMS3,STMN2,LINC00675,SPPL2A,OLFM1,KCTD10,TLCD2,UPP1,GPER1,PPP1R12B,ZBTB16,KIAA0513,PDLIM2,CPT2,UNC5B,ATP5S,CACNB2,DCLK1,MUC4,CLTB,DGKA,B4GALT1,PPP1R16B,DOCK5,SDCBP2,SGK1,DYRK2,SNRK,ACAA1,BTNL8,LRP10,CHP1,CDKN1A,SCN7A,E2F2,HOXD1,SULT1A2,CPNE8,CD79A,LRRFIP2,AHNAK,CENPT,FOSB,PTPRCAP,B3GNT6,DENND2A,DPF3,PQLC1,CYTH1,PRELP,MYO7B,GMPPB,CLCN2,ADTRP,DHRS11,VPS37B,MAL,PC,CLIC5,TMEM171,SMAD3,FDCSP,LOC102724156,CR2,MFAP5,MFSD4A,PLEKHG6,PLXNA2,MMP15,GPAT3,CEACAM7,MED16,PTK2B,VIP, GIMAP5,SYNPO,SEMA6A,CDA,THSD4,P3H2,CCDC85C,ITM2C,FLVCR2,FAM132A,NKX2-3,MYO15B,KLF6,LOC101928405,TMCC3,PCSK7,ABCC3,MYH11,ACADVL,UGDH,HSD11B2,MYOM1,PPP2R3A,PTPN21,OAF,PRKCB,PITPNM3,ABCC13,ETHE1,XDH,IGHM,CD1D,LPAR1,HNF4A,CCDC68,RGMA,SH3BGRL3,UGP2,CHCHD10,TJP3,ATP6V0D1,KCNMA1,SECTM1,GAREM1,LMOD1,PLP1,KIF13B,B4GALNT3,TCF21,SOCS6,SULT1A1,FCRL3,ACVRL1,CBFA2T3,INF2,SCG2,ABI3BP,PLCXD1,CP,RAPGEFL1,ZFP36,SLC51B,MS4A12,ACACB,RNF10,KRT24,ENTPD3,EPAS1,STX12,MINK1,PEX26,SPSB1,MT1X,MAFF,HAGLR,MAP2K3,FAM189A2,LINC01279,RERGL,FABP4,CCR7,METTL7A,AQP8,SLC22A5,SHROOM3,CA2,ELMSAN1,ANKRD9,DHRS9,NDRG2,IL1R2,SGK2,SFN,FFAR4,MGLL,TRIM15,LGALSL,OSBPL7,EFHD2,ENDOD1,PLCE1,ZNF341,FAM126B,NAPSB,MT1F,TCL1A,ITM2A,CR1,RHOC,EPB41L3,KLK15,LOC285097,PRPH,C1QTNF7,STYK1,CSRNP1,CAPN9,CNN1,CDHR2,CDKN2B,OASL,PIM1,CNNM4,TMEM54,SLC9A2,PRIMA1,VILL,FAM46A,SEMA4B,EPB41L4A,MMRN1,CASQ2,MVP,DPP10AS1,LOC284578,SGMS2,CCL28,MICAL2,NBL1,SIDT1,LRRFIP1,METRNL,MOGAT3,MISP,BCAR3,AMN,NR5A2,KIAA2022,INPP5A,TMEM220,TIAM1,TP53I3,PKIB,FLNB,LTF,ST3GAL4,ACO2,DUSP1,CYP4F12,BAG1,BMP2,DES,HMOX1,MT1H,CALM1,ISG20,AHCYL2,SYNPO2,TLN2,SYTL4,SSPN,HHLA2,FHL1,LITAF,MT2A,NAAA,MYO1A,MIR22HG,SERINC2,TAX1BP3,KLF9,THBS1,BDKRB1,ANK2,CES3,P2RY1,GAP43,PLEKHA6,S100A16,EMP3,FA2H,CD37,MT1G,NXPE1,GLDN,FUCA1,PRSS12,CHRNA3,CHP2,SLC25A20,TNS1,VIPR1,RELL1,SCAMP4,FUT3,MT1E,AGPAT2,PTP4A1,BCL10,SPON1,LRMP,TNFSF13,CWH43,AVPI1,MLXIP,MUC2,LGALS9,FUT6,CCNYL1,CEACAM1,HCLS1,SLC35D1,CDKL1,LTK,ELOVL6,TUBB2A,CGN,MIB2,KIAA1211,TAGLN,GALNT5,TRPM6,UGT1A3,TRPM4,SSU72,NBL1,RETSAT,MOGAT2,NDEL1,GHR,SLC17A5,PAG1,SAMD9,EHD4,CORO2A,SLC28A2,CD36,MAMDC4,MBOAT1,KIF1C,SQRDL,OR7E14P,ALS2CL,MSRA,TSPAN1,IL10RB,ANGPTL1,SCGB2A1,AKR1B10,PLA2G10,LPP,FXYD6,LDB3,FAM43A,MIER1,FZD8,SEMA4G,ADIPOQ,PPP2CB,NEDD9,QSOX1,F2RL1,MS4A1,SORBS2,PRR5L,TTC22,TOX,MGAT4A,CSRP1,SRPX,GIMAP1,LINC00341,MZB1,MRGPRF,MOB3B,ITIH5,CDC42EP5,CYSTM1,UGT1A1,TMEM164,MFSD2A,MFAP4,MYZAP,LYPD8,ATP8B1,PARM1,RIOK3,BCHE,RBM24,MYLK,SERPINB8,INPP1,CCDC69,PRDX6,PEG3,LIFR,RSAD2,PTGS1,CCNG2,VLDLR,DUSP5,STARD10,TSPAN7,PPID,TNFSF13,HSD17B2,RARRES2,RTN1,RPS6KA1,LGALS2,PARVA,RAB27A,SLC22A18,C2orf72,LIPH,TUSC3,GIMAP8,DNER,HOXB13,LGALS3,PPP3CB,HBG1,INO80C,MPC1,GDA,SLC25A25AS1,CFD,THRB,MEIS1,LAMA1,MUC12,GSTA1,ITLN1,TPH1,LONRF2,NR3C2,GPM6A,SLIT2,CXCL13,C1orf226,TP73AS1,KIF5C,APBB1IP,GRAMD3,ABHD6,CES2,LOC101928152,TMBIM1,TFCP2L1,SUPT20H,C16orf62,FLNC,LOC100505501,KIF16B,AKR7A3,HBA1,TST,PGM5AS1,ARHGAP9,STBD1,TCF7L2,ECHDC2,ETFDH,ADAMTS1,LOC100288911,DSC2,CAMK2N1,MEP1B,RBPMS2,GPD1L,MAB21L2,FAM107B,PTGIS,RCAN1,CASP7,SHE,STK17B,STAP2,PGM5,C16orf54,CLCA1,MYOCD,FBLN1,ASPA,PIGZ,NR1H4,FBLN2,CNR1,CD59,SLC16A9,EIF4E3,C1orf115,CKB,BCAS1,GPA33,ANPEP,ATG4A,NANS,PLEKHO1,PDK4,MUC5B,ANO5,TTC38,SETBP1,SMPDL3A,SULT1A3,SLC22A23,FAM101A,RSPO3,HPSE,GPX3,ACSS2,GGT6,DNASE1L3,SOWAHC,FCGBP,CCL15,ARL14,LMO3,PHLPP2,MCL1,SMIM14,GALM,COL14A1,UQCRC1,CAPN13,C14orf132,VDR,ADCY9,CTSE,CDH19,AOC1,RCSD1,FAM118B,PLCL2,SEPW1,CAPN8,F11R,MAOA,ATP1A2,WFDC2,ETS1,COX7A1,GBA3,IGH,ACTG2,SLC17A4,ANTXR2,TMEM37,SYNM,CCDC80,PLCG2,TMEM35A,ACAA2,UBA7,ARRB1,CNTN4,SELENBP1,PROM2,MT1HL1,HAND2AS1,MASP1,FAM114A1,AOC3,EP300AS1,SIPA1L2,C4orf19,PLLP,HSPB8,CDIP1,FAM129A,SLC51A,SLC36A1,FRMD3,GOLM1,EPS8L3,SYNE1,TRBC1,S100A14,CCL5,SDPR,ATP2B1,TCEAL3,SGPP2,PRKAR2B,PLAC8,FAM46C,C3orf52,CYP2C18,LAMA3,RHOU,LOC728392,FTH1,TUBAL3,PPP1R14A,PRSS3,EPB41L4B,TMEM246,SLC6A8,HBEGF,HEPACAM2,HSD3B2,COL6A2,ADH1C,FMO5,STS,HLX,TNFSF10,SIAE,MUC1,RNF150,LRRC19,FLRT2,SELL,MYL9,LIMA1,FGFBP1,DPP6,FNBP1,CECR1,BIRC3,BHLHE41,IL2RG,MUC13,HBB,NAP1L3,SLC13A2,CPQ,RUNDC3B,PAPSS2,SLC26A3,FXYD3,POU2AF1,PIP5K1B,CRYAB,TNFRSF11A,AIFM3,TCEAL2,PDE4D,PAX5,SYNC,PGM1,TM4SF1,KLK1,SI,EMCN,TCEA3,PDZRN4,CLMN,HPGDS,LRRC66,IL18,CDC42SE2,EDN3,NR4A2,FOXA3,ARRDC4,CYCS,SFRP2,GNE,GNAI1,CMBL,IGLC1,GCNT3,RBFOX3,CD48,FRMD4B,HGD,ABHD3,RCAN2,CLDN11,C1orf21,PTGDR,PIK3CG,JAM3,RNASE1,RGS13,SCNN1A,AKR1C1,FAM127A,MAOB,SLC46A3,WLS,BRINP3,NOV,PIGR,UBE2QL1,KRT20,IGKC,ZNF655,IGH,NR4A3,HPGD,C1QA,PLA2G2A,PCK1,ATF3,ISX,RARRES1,PTGER4,NXPE4,PLN,CREB3L1,PDLIM3,AGTR1,SLC41A2,EPHX2,FKBP1B,IGLL5,HK2,MEIS3P1,SLC44A1,SLC2A13,FOS,NCAM1,ROR1,HYAL1,RFTN1,LINC00483,SLC1CKMT1B,EVI2B,FGFR2,C10orf99,KCTD9,HSPA2,PCSK5,CASP5,INSM1,CMAHP,TCEAL7,PCOLCE2,EFEMP1,MGP,HDGFRP3,MLPH,CAV1,EFNA2,ZFPM2,CRIP1,NOSTRIN,HMGCS2,GPRC5A,LOC105375172,LOC102725051,WDR78,CHGB,PDE3A,LINC01133,KAT2B,HIST1H3F,HDAC9,NCKAP1L,FAM134B,MAP1B,IGKV1OR2108,EFHC2,GLYR1,LOC101930400,EGLN3,SULT1B1,OSBPL1A,ACTA2,CYBRD1,CHFR,HSPA1B,ZSCAN18,IGK,BCL2,SPARCL1,AGR2,ADM,BASP1,TMEM30B,SPINK4,NR3C1,VWA5A,CNTN3,LGALS4,C3,REG4,ADORA2B,EDIL3,FABP1,CD52,CFH,GBP3,SLC26A2,HLADPB1,C15orf48,FGL2,IGLC1,C8orf4,NDN,PRKAA2,CTSS,PID1,CATSPERB,PRKACB,FOXF2,CLIC6,C3orf70,EPHA4,SIK1,ADRA2A,FOXP2,SEPP1,TNFRSF17,KCTD12,NBEA,TFF1,MEP1A,IGLL3P,KLRB1,ST6GALNAC1,AXDND1,IGLC1,RASSF6,IGLV144,PCDH20,BCL2L15,NPY1R,ZC3H12C,AGR3,MSRB3,FAM3D,UGT2A3,HOXD13,BEX4,JCHAIN,LINC00261,SSTR1,IQGAP2,HLADQA1,P2RY14,ST6GAL2,SYTL5,TAC1,DPP10,ABCB1,HLADQA1,MATN2,ABCB1,DNAJC12,LINC01207,MUC17,GREM1 | CLDN1,AJUBA,PPAT,XPOT,ATP11A,NFE2L3,DIEXF,SLC6A6,RPL36A,WDR75,ZC3HAV1L,CKAP2,CBX3,SLC39A10,SOX4,FOXQ1,ZNF121,TMEM123,NIT2,NIFK,PPM1H,TGDS,MRE11A,SNRPD2,DKC1,AASDHPPT,WDR12,ZFAS1,NUDCD1,CSE1L,IRF2BP2,PWP1,CPSF3,PLEKHA8,DPY30,FAM60A,CEMIP,ASUN,BUB3,CD46,MYC,INHBA,PRMT3,HEATR1,HS2ST1,BRIX1,NUFIP1,CBFB,PPIL1,LRP11,DARS,CCNB1IP1,BZW2,IFT80,ORC3,SSB,FAM220A,TRIM59,SOX9,PHF14,RNF219,E2F6,NFXL1,EIF3E,ADNP,RIF1,MACC1,PTPN4,MTR,RPS15A,DUS4L,ASCL2,CFAP97,UBE3D,URI1,DNAJC2,GTPBP4,ADO,ZNF146,ZBTB44,TGFBI,NAP1L1,KIAA1033,SMYD2,PMAIP1,RFC3,ACTL6A,IPO5,HACD3,CCT2,DCAF13,XPO1,NOL8,RPGRIP1L,CEP57,SDAD1,RPS20,THUMPD2,RBM12B,THNSL1,AZIN1,PMS1,TATDN1,CCDC59,PNPT1,WDR3,GTPBP10,BOD1,CEP68,C1orf109,POT1,MSI2,TGS1,METTL5,DIS3,C12orf29,MINA,ZNHIT6,OSBPL3,MTERF3,RPF2,TXLNG,ZC3H8,NUP37,ALG11,SRFBP1,NPM1,TXNRD3,IPO7,GART,SLC22A3,DDX21,LMBR1,NOLC1,RPAP3,EIF2S2,GTF2F2,RECQL,MRPS23,SNORD29,WDR43,PDCD2,PRIM1,LOC107985971,NUP35,TSEN15,PFDN4,ZNF281,PAICS,SHPRH,LZTFL1,LTV1,CCT6A,EIF2A,TRMT5,DDX31,NOC3L,VSNL1,TARBP1,NUF2,POLR1B,RPS7,GTF3C3,GNL3,GNPDA1,UTP14A,CLNS1A,ABCE1,RAD54B,POLR1C,EIF3M,TMA16,DCUN1D5,DPH6,PEX3,FLJ45482,CMSS1,NUPL2,NEBL,NAA25,HSPE1,GEMIN6,CCSAP,POLR1D,HILPDA,TBC1D4,IRAK1BP1,CKS2,RCBTB1,LRPPRC,NKRF,FIGNL1,SLC39A6,UTP18,C8orf59,ARL6IP6,ZNF280C,CKS1B,ARMC10,CDCA7,SNORD87TRMT11,ANLN,TPP2,IMMP2L,GPSM2,CDK1,C11orf1,EEF1E1,TTC26,VPS35,ZNF627,SNORD1A,TIGD2,TMEM216,MET,ATR,UBR5-AS1,TSEN2,FBXO5,LIMS1,RBM39,UBA2,BTF3L4,U2SURP,TOMM20,GEMIN5,CSPP1,MRPL30,YEATS2,GPALPP1,EIF1AX,TTC27,NUP155,NOM1,TAF1B,RFC4,USP31,LYPLA1,SRSF7, GTF2I,RPE,TOP2A,AP3M2,PUS7L,CXXC5,PTK2,GPATCH2,SPATA13,POLR2K,SNTB1,UBE2W,HAUS3,WDR36,HAUS6,NMD3,ACP1,PLAU,SSBP1,BICD1,EXOSC3,GLO1,SNORD81,PUS7,TRIM24,KIF3A,PAIP1,MEST,QTRT2,NEMP1,SNORD61,ZNF7,PCMTD2,KIAA1549,MRPL17,ECT2,NUDT21,ORC5,MRPS17,IFITM1,ZNF250,G3BP1,NUP160,RPL37A,TBC1D31,TCEA1,EPHX4,SCML1,TAF1D,FAN1,CBLL1,PCID2,DBR1,YEATS4,TCFL5,ZNF12,KRR1,SPDL1,PIBF1,WDR5B,BORA,ZNF277,ZNF239,ASPM,NARS2,DDX18,SET,MTFR1,TWISTNB,POC5,EXOSC8,CPOX,ANAPC10,MRPL42,AIDA,ZNF107,MRPS31,C11orf58,FBXL4,RAD50,ZNF567,ATAD2,OSGEPL1,CDADC1,ZNF451,NUP43,TAF1D,AMZ2,NAA16,TIA1,ERGIC2,CDC5L,ZNF485,SCAMP1,FAM89A,GMPS,CD44,EPRS,UBE2T,CBX4,CLUAP1,CENPN,DDX10,LIPA,GLCE,DIMT1,ENOPH1,SNORD50A,USPL1,PRICKLE4,INTS7,DBF4,PSPH,RCN2,KNOP1,AXIN2,DUSP14,ZBTB26,VKORC1L1,DHX9,TMEM267,RRP15,ACVR2B,MAD2L1,LDLRAD3,GPR180,GTF2H5,RAD51AP1,C12orf66,TAF1A,USP6NL,NOL11,FOPNL,PROSER1,THOC2,MNAT1, GCSH,MSH6,SCARNA13,CRNDE,FMNL2,PTPN12,RPP40,LOC101927204,RB1CC1,CENPJ,CENPF,LSM8,BCLAF1,GTF3C4,UBN2,SMG8,OGFOD1,TEX10,SMAD5,RAN,RSL24D1,UTP4,C5orf34,FANCF,ZNF623,EIF2S3,MPHOSPH9,AHCY,MSH2,IFITM1,ZFAND1,ZFR,RHOT1,CSTF1,TMEM260,TMEM209,HOMER1,DSCC1,INTS8,CHCHD7,TFB2M,ZNF22,ERCC6L,GXYLT1,GTF3A,EFCAB11,DPH5,PNO1,MRS2,NUP58,LYAR,FUT1,PAXIP1,CREBZF,UFM1,YAP1,CDH3,KIF18A,MPLKIP,MCM3APAS1,UFL1,SCD,BID,PTBP3,LRRC58,RIPK2,SLC12A2,PPT1,COPS8,SLC7A5,ZNF420,MZT1,SRSF10,GPN3,INTS2,ABCC1,SORD,SPC25,LARS,SUPT20H,GRPEL2,KIF20B,FOXP4,AS1,ZCCHC7,CTHRC1,BUB1,EIF3H,CEP83,HMGB3,EBLN3P,CETN2,SLC25A36,ZNF195,RCL1,CEP41,CCDC138,NME1,C10orf2,IFT80,PARPBP,UPF3A,XPO4,JADE3,DTL,ATP6V1C1,ZNF200,TRNT1,FUNDC1,TFAM,GLMN,ZNF140,TMEM126B,TIGD1,PAK1IP1,OGT,FAM72A,BBX,ZNF770,MANEA,NXT2,ZNF138,OTUD6B,CACYBP,RNF43,TBL1XR1,PALD1,HMMR,MOB1A,PROX1,PSTK,XPNPEP3,KNTC1,E2F5,ITPRIPL2,TIMM8A,HMGB1,HELLS,BBS10,PRDX4,ZWILCH,LSM5,G2E3,ITGB3BP,NFYB,RBPJ,SKP2,MORC2,LACTB2,RSRC1,RCN1,LOC101928195,TRIB3,UGGT2,INTS6,SRPK2,MRPS25,RANBP2,TUBGCP3,NR2C1,EBPL,SMARCC1,FZD6,TMEM243,CDKN3,SGO2,EARS2,MTFR2,LOC729680,ACSL4,CHD6,ENC1,LOC100134445,MIPEPP3,ARID5B,NEK2,MASTL,SRD5A1,POLR3C,AGK,SLC11A2,TRIP13,ARHGAP18,ZMYM2,DPY19L4,UTP23,DLGAP5,C17orf75,GNAS,DHX40,CDC16,EFCAB7,M6PR,CHML,ACTR3B,C4orf46,SETDB2,SUV39H2,HIKESHI,XRCC4,TRAF5,FBXO45,PTPMT1,RBM28,BICD2,SNHG4,CAPRIN1,SF3B1,ZNF670,PPWD1,TTC17,ZNF322,SLC25A15,DNAAF2,C12orf73,RARS2,FABP6,RP9,HDAC2,FBXO21,RFC5,CENPQ,CCNJ,TCEB3AS1,OSBPL8,C11orf80,CEP152,TDRKH,BRCA2,ASAP1,NUDCD2,ZNF84,RBM22,PODXL,TMPO,ARNTL2,KIF20A,SVIP,NCAPG2,ENOX2,PXYLP1,AZGP1,E2F7,UBXN7,ZNF253,MITD1,STK4,ANXA9,SYBU,PRPS1,VMA21,GTF2IRD1,HIBADH,APPL1,SPATA7,BCCIP,LOC102724951,CPNE3,COL11A1,GINS3,TTC8,RAE1,PTCD2,NEIL3,STK3,TCEB1,STX6,ZNF680,USP48,BLACAT1,SS18L1,ZNF92,MRPL50,BUB1B,DNMT3B,CSGALNACT2,PSPC1,FAM92A1,KBTBD6,CCDC34,RACGAP1,PDZD8,MAGOHB,NFAT5,KLF7,EZH2,HCG18,ADD3,GLT8D1,NUSAP1,EPSTI1,CDK6,ZNF638IT1,P4HA1,USP1,TMPRSS3,TMTC4,UCHL3,MLLT10,GPR160,POLK,TTK,LINCPINT,FNTA,CHEK1,HSPB11,HSPH1,PAN3,HJURP,CNPY2,MGA,PSMG4,HSF2,COX20,TULP3,BRWD3,NEDD1,ZBED8,TMEM165,CCDC113,COPG2,EEF1AKMT1,HSPD1,CELSR3,PTPN11,DPEP1,POLA1,VEZF1,C1GALT1,SUZ12,CKAP5,ABHD10,AURKA,FAIM,PLEKHA8P1,PRMT6,PPFIBP1,NCOA3,ADK,CEP72,BRD7,LOC101930489,PROCR,TRIM27,IARS,USP28,PPP1R3D,ZNF33B,UNG,CDC40,LNPK,ZAK,CDK4,ASPH,DPCD,TRIM4,KIF14,STIL,RBM41,KPNA2,BRIP1,SMC4,TTI1,SMC2,GPAM,ASF1A,CTC338M12.4,CEP55,SNHG8,SSX2IP,MAP3K7,CASK,SETD6,TMEM206,FAM217B,CMTM8,SLC25A32,KIF4A,API5,ASXL1,SNAPC1,OTUD6BAS1,FXR1,PSAT1,CEP192,MKLN1,WDHD1,MTPAP,FSBP,AUNIP,RFXAP,PHOSPHO2KLHL23,COA1,NAMPT,C6orf48,KRIT1,NOB1,USP46,ZMYM5,CDK8,EXOSC5,RFX7,TUBE1,ATRX,NCAPG,CYP39A1,AGO3,LOC730101,GTF2H3,FLVCR1,PDP1,ZNRF3,NPEPL1,C5orf24,CEBPZOS,CCDC77,STAG2,WDR35,MIR17HG,ESF1,KIAA1524,EGFL6,HNRNPDL,CTPS2,FGFR1OP2,RAD18,PABPC4,GRHL1,SNHG15,RPS24,NAA15,TTF2,ERCC8,MBTPS2,ITGA2,CHEK2,FSD1L,SUZ12,PHF10,DEPDC1B,LOC101928433,KIAA1958,SYNCRIP,MTHFD2,ARL13B,MED1,NNTAS1,PDCD2L,SMG1,SNRPD1,RRP1B,NANP,TIMM8B,GGH,APEX1,FAM84B,OXR1,ZNF367,SNX25,CENPE,SMYD3,KNSTRN,NEDD4,MCM6,PPP2R5C,DENR,ZKSCAN8,ZNF32,RFWD3,SRSF1,PHF20L1,CERS6,TGIF2,MED28,RPL29,POLR2D,SUB1,WNT3,SPIN3,DIO2,MBNL2,LGR5,SIM2,BIVM,SKA3,UTP20,CENPK,TOP1MT,SRP72,LOC100287896,RPL31,TNPO3,ZDHHC9,ABI2,NCK1AS1,C11orf95,TTC3,TFDP1,FANCI,PHF20,CXCL8,METTL8,CAMTA1,ADSL,PRPF4B,TPX2,THSD1,DDIAS,PSRC1,SNRPF,MSANTD4,ASCC3,PATZ1,SMC3,CTPS1,KNL1,GZF1,SLC35F6,KIF23,MIR1204,GRIN2D,KBTBD7,PSMA7,ALG10B,RPRD1A,RPL22L1,SDCCAG3,KIAA1586,MTAP,DDX52,PRKDC,TEX30,TSPAN5,TUBD1,TNFSF15,SNHG17,GLS,CXCL3,ENAH,CLCN5,ZNF697,ENPP5,CUL4B,SMS,TUG1,SNORD68,PLS3,ZNF780A,TIPIN,GINS1,LRRC6,DLEU2,SET,SEMA4D,TBC1D16,MIS18A,UBA6,PTTG1,RUVBL1,TMCC1,ZNF280B,WDYHV1,RLN2,KIF15,UCHL5,LINC01184,KPNA5,LRP8,POFUT1,TDGF1,LOC727820,CBX5,TPR,DACH1,POGK,NSMCE2,DGAT2,PCNA,KDELC1,FZD3,FUT8,NBN,PSMG1,NONO,TCAF1,DONSON,NOP2,UBE2V1,GALNT6,UBE2C,C1orf112,RSL1D1,ZC3H7A,NR1D2,MIR181A2HG,HSP90AB1,NDC80,ARHGEF28,ANAPC7,CCNB1,CENPI,SMKR1,EIF5A2,PAPOLA,DCTD,HNRNPD,SLCO4A1,AGGF1,C8orf33,SH3BP4,ATF1,EHBP1,DCAF16,ING1,METTL2B,DEPDC1,SNORA21,RASSF10,KIF11,MORF4L2,PHLDA1,CDC25C,UQCRC2,NIP7,ANP32E,LYRM4,RPL23,MND1,UQCC1,RAB15,ILF2,CENPU,DNAJC10,MTDH,KIAA0895,PTPMT1,KLHL24,PFKM,BCOR,DHX33,LYRM2,SPIN4,PKP4,ZKSCAN1,LEF1,DLEU1,TPD52L1,SUPT3H,MOSPD1,F2R,FOLR1,TMEM68,GLS2,KIF5B,NEK3,PITX1,OIP5,LPCAT2,MCM10,MCM3,ASNS,VEGFA,CBX2,CENPL,MGME1,DIAPH3,EDNRA,POLR3G,TMEM231,MTRF1,PABPC1L,CLCN4,VASH2,NHS,RYK,CNOT1,FAM200A,SNORD14D,BAG5,KRT23,RBL1,MCM4,CANX,TMEM9,TMEM245,XPO5,LZTS3,MKI67,STC2,LDAH,MIR1292,CXCL1,TSR1,PBK,CBX1,RANBP1,SLC16A1AS1,MORC4,ZNF251,EIF4B,MRTO4,COG3,KLHL7,CCNA2,FBL,NUP62CL,PHKA1,CEP76,BBOX1AS1,POLE2,FARP1,STAT1,ZRANB3,CBFA2T2,LOC654342,KRT80,CENPH,FLT1,CACNA1D,NEK5,UHRF1,ARID3A,EIF3B,POMZP3,ATP6V1C2,TRAM1,FBXO41,ZFP90,NORAD,LAMB1,MSH5SAPCD1,RNF183,C2,LOC101930107,MTHFD1,TGIF1,RRM2,E2F8,BACE2,RRN3P1,KPNA3,NPM3,TEAD4,TMEM97,FRMD5,CTSH,CADPS,NOD2,NELFCD,PM20D2,KIAA0101,MED30,FAM83D,APBB2,DLGAP1AS2,SLC22A15,MALAT1,PTPRO,PITX2,KIAA1551,ARL4C,PRR11,DEPTOR,WDR74,PRC1,SLC7A7,CTTNBP2,SLC16A4,SMN2,ACSL6,LOC103091866,PAAF1,KIF2C,EPB41L4AAS1,RNASEH2A,MRPL45,CDC6,RUNX2,ETV4,FAM210B,IL20RA,XRN2,ALDH5A1,GRB10,TMEM17,CFB,HNRNPR,SLC2A12,MINCR,TBC1D8,PGK1,GNG4,LINC00467,SFXN3,ELMO2,TP53RK,CKMT2,SLC6A20,RNF114,DCBLD2,BMP4,AZGP1P1,MCM2,PLAGL2,DYX1C1,JPX,CALU,CCNB2,RHOQ,MMP7,MMP1,PCNX2,ADGRG1,TMEM185B,HMGA2,MEGF6,TMEM106B,MIR3652,GZMB,SACS,CHORDC1,CHI3L1,CDCA5,GUF1,MSX1,UNK,ABCC4,PLCB1,LY6G6F,HENMT1,KIAA1257,LINC00920,LDHB,MPP6,ZNF711,QPCT,SPP1,SEH1L,LOC101930415,LAPTM4B,MMP3,LRRC8E,FAM216A,PLCB4,USP9X,AGFG1,GRHL3,CXCL11,SQLE,ANKRD10IT1,RPL37,MATR3,GPR143,PLA2G16,TAF4B,TMTC2,PRSS23,TESC,SLC35D3,TBX3,MSX2, PAH,SHMT2,S100A2,TACSTD2,MCM8,PSTPIP2,HOXB8,CXCL10,SAMD12,KLK6,PAN3-AS1,CDC25A,RHOBTB3,NKD1,SRPX2,WT1,EPHB1,COCH,NR2F1,LIPG,WDR72,APCDD1,TTC9,TNMD,SLCO1B3,TFPI,DUXAP10,CEL,GSTO2,ZNF185,PTPN13,DUXAP10,LARP6,SLC4A11,FGF18,NETO2,ALDH1L2,ZNF503,ERP27,CYP4X1,GRK3,GDF15,PLAG1,COL9A3,EDAR,SFTA2,CLDN2,FGGY,CAB39L,MME,LOC101930067,PIPOX,HOXB3,PLEKHB1,DSG3,NQO1,REG3A,ONECUT2,BMP7,GAD1,DSC3,MAP7D2,PRSS33,COL27A1,TMEM64,KRT6B,ZIC2,WIF1,TCN1,PROSER2,DHRS2,KLK10,IGF2BP3,UCA1,STMN3,TFAP2A,RNF182,SBSPON, H19,BAMBI,TNFRSF19,CXCL5,PPBP,SLC44A5,LYZ |

**Table S4.** List of upregulated and downregulated shared DEGs (sDEGs) between CRC and T2D

| **Downregulated sDEGs** | **Upregulated sDEGs** |
| --- | --- |
| MMP28,ST6GALNAC6,SCARA5,ACKR1,DHDDS,CLDN23,PHYKPL,B3GALT4,CIDEC,GSN,TRANK1,JAM2,CLDN5,PLPP1,MALL,EMP1,DPT,CCL19,GPER1,DCLK1,AHNAK,MAL,PC,OAF,LPAR1,INF2,ABI3BP,PLCXD1,MGLL,PRPH,TMEM54,TP53I3,FLNB,SYTL4,THBS1,VIPR1,AGPAT2,NBL1,CORO2A,MBOAT1,MSRA,MOB3B,ITIH5,CDC42EP5,MFAP4,MYLK,RTN1,GIMAP8,GRAMD3,CAMK2N1,RBPMS2,FAM107B,FBLN1,FBLN2,SOWAHC,SMIM14,COL14A1,MAOA,ACTG2,ANTXR2,TMEM37,FAM114A1,CCL5,FTH1,COL6A2,TNFSF10,MYL9,LIMA1,CPQ,SFRP2,JAM3,RARRES1,HK2,MEIS3P1,SLC44A1,RFTN1,PCSK5,PCOLCE2,EFEMP1,MGP,CAV1,CRIP1,MAP1B | HBEGF, BZW2, SMYD2, PTK2, XPO4, PROX1, ENC1, VEGFA, COL18A1, CKMT2, FGGY, CD44, MYH3, SMARCD3, TRIM69, PLS3, IP6K3, CIART, LRP12, HCFC1R1, PROX1, IGSF11, SCN3B, SHISA2, CLTCL1, RORC, MYH7B, CLTCL1, THAP4, PCNT, LGALSL, USMG5, CFAP61, SLC16A10 |

**Table S5. Identification of shared DEGs (sDEGs) between T2D and CRC**

| HACD3, PTK2, TCFL5, CD44, SCD, KIF20B, XPO4, PROX1, ENC1, ADD3, TRIM4, TOP1MT, PLS3, VGFA, EDNRA, ARID3A, CKMT2, CALD1, MSX1, SRPX2, FGGY, MMP28, SCARA5, ACKR1, SLC25A34, DHDDS, CLDN23, B3GALT4, CIDEC, GSN, TRANK1, JAM2, CLDN5, PLPP1, MALL, EMP1, DPT, CCL19, GPER1, DCLK1, AHNAK, MAL, PC, CLIC5, MYOM1, PPP2R3A, LPAR1, UGP2, INF2, ABI3BP, CA2, MGLL, PRPH, TMEM54, CASQ2, TP53I3, FLNB, VIPR1, CORO2A, MBOAT1, LDB3, MOB3B, ITIH5, MFAP4, MYLK, RTN1, GIMAP8, FLNC, CAMK2N1, RBPMS2, THBS1, FAM107B, FBLN1, FBLN2, SMM14, COL14A1, MAOA, ACTG2, COL18A1, ANTXR2, TMEM37, CCL5, FTH1, TNFSF10, MYL9, LIMA1, CPQ, SFRP2, JAM3, NR4A3, HK2, PCSK5, PCOLCE2, EFEMP1, MGP, HBEGF, CAV1, MAP1B, CYBRD1, CFH, FGL2, NDN, C3orf70, KCTD12 |
| --- |

**Table S6.** List of shared key genes (sKGs) from PPI network based on different topological measures

| SN | List of shared key genes (sKGs) from PPI network based on different topological measures | | | | | | |
| --- | --- | --- | --- | --- | --- | --- | --- |
| 1 | SKGs | EPC | Closeness | Degree | MNC | Radiality | Stress |
| 2 | PTK2 | 33.31 | 33.51 | 11 | 9 | 5.26 | 1300 |
| 3 | COL18A1 | 35.281 | 28.95 | 8 | 8 | 5.01 | 990 |
| 4 | CD44 | 36.837 | 38.28 | 16 | 13 | 5.40 | 3550 |
| 5 | PLS3 | 34.689 | 45.78 | 18 | 19 | 5.80 | 1790 |
| 6 | CLDN5 | 33.891 | 63.17 | 31 | 30 | 4.28 | 2820 |
| 7 | THBS1 | 36.566 | 36.81 | 13 | 12 | 5.36 | 2416 |
| 8 | CAV1 | 35.968 | 39.50 | 16 | 7 | 5.53 | 5748 |
| 9 | EFEMP1 | 35.388 | 31.75 | 10 | 9 | 4.95 | 1370 |

| **Table S7.** The significant prognostic value of CpG in sKGs | | | | | |
| --- | --- | --- | --- | --- | --- |
| **sKGs** | **Gene Group** | **CpG Island** | **CPG Name** | **HR** | ***P*-Value** |
| CD44 | Body | S_Shore | cg01766065 | 0.561 | 0.021481218 |
| CD44 | Body | S_Shore | cg21076259 | 0.62 | 0.05907524 |
| PTK2 | 5'UTR | Island | cg06119711 | 1.736 | 0.022971156 |
| PTK2 | Body | Open_Sea | cg06944982 | 2.076 | 0.042109976 |
| PTK2 | Body | Open_Sea | cg11559446 | 2.009 | 0.04219248 |
| CAV1 | Body | Island | cg00308439 | 2.253 | 0.011044122 |
| CAV1 | TSS1500 | N_Shore | cg01265597 | 0.585 | 0.031001862 |
| CAV1 | Body | Open_Sea | cg18498156 | 1.833 | 0.057055644 |
| EFEMP1 | Body | Open_Sea | cg05140065 | 1.907 | 0.011002606 |
| EFEMP1 | TSS1500 | S_Shore | cg25412594 | 2.398 | 0.014492893 |
| PLS3 | 5'UTR | Island | cg21539234 | 0.539 | 0.012234 |
| CLDN5 | 1stExon; Body | Island | cg17577122 | 1.615 | 0.054182 |
| CLDN5 | 1stExon;3'UTR | Island | cg21872764 | 2.265 | 0.013643 |

**Table S8.** Docking scores (binding affinities, kcal/mol) between the proposed receptors and top ordered 30 candidate drugs (out of 307)

| **name** | **CD44** | **EFEMP1** | **TP53** | **COL18A1** | **THBS1** | **PLS3** | **CLDN5** | **EGR1** | **CAV1** | **SP1** | **PTK2** | **Average of Drug ordering scores** |
| --- | --- | --- | --- | --- | --- | --- | --- | --- | --- | --- | --- | --- |
| Irinotecan | -9.4 | -7.6 | -8.4 | -7.1 | -7.2 | -7.1 | -7.4 | -7.4 | -7.1 | -7.3 | -7.4 | -7.6 |
| Leucovorincalcium | -9.8 | -8.2 | -8.2 | -7.4 | -7.4 | -7.2 | -7.5 | -7.1 | -7.1 | -7.3 | -5.9 | -7.5 |
| Regorafenib | -8.5 | -8.1 | -8 | -7.7 | -7.1 | -7.2 | -7.6 | -7.4 | -7.1 | -6.6 | -5.5 | -7.3 |
| Fenretinide | -8.3 | -7.8 | -7.3 | -7.1 | -7.2 | -7.1 | -7.8 | -7.1 | -7.1 | -6.3 | -6 | -7.1 |
| Ouabain | -7.7 | -7.5 | -7.4 | -7 | -7.3 | -7.6 | -7.6 | -6.6 | -6.2 | -6.3 | -6 | -7.0 |
| Doxorubicin | -7.7 | -7.8 | -7.1 | -7.1 | -7.6 | -6.4 | -6.6 | -6.8 | -6.5 | -5.8 | -6.2 | -7.0 |
| Gentamicins | -7.9 | -8.4 | -7.2 | -7.8 | -7.1 | -6.1 | -6.5 | -7.3 | -6.8 | -4.6 | -5.6 | -7.0 |
| Miquelianin | -7.2 | -7.1 | -7.3 | -7.7 | -7.1 | -7.7 | -7.1 | -6.1 | -6 | -6.3 | -5.5 | -6.9 |
| EthinylEstradiol | -7.9 | -7.9 | -7.3 | -7.2 | -7.1 | -7.5 | -6.4 | -5.9 | -6.3 | -5.7 | -5.8 | -6.8 |
| Mifepristone | -7.1 | -7.1 | -7.7 | -7.3 | -7.3 | -6.4 | -6.2 | -6.5 | -6.7 | -5.9 | -6.4 | -6.8 |
| Nitrobenzanthrone | -8.2 | -7.7 | -7.2 | -7.1 | -6.2 | -6.5 | -6.2 | -5.8 | -6.5 | -6 | -5.9 | -6.7 |
| AflatoxinB1 | -8 | -7.7 | -7.1 | -7.1 | -6.3 | -6 | -6.5 | -6.8 | -6.1 | -6.2 | -6.1 | -6.7 |
| Dorsomorphin | -8.3 | -7.4 | -7.2 | -7.2 | -6.6 | -6.8 | -6.2 | -6.5 | -5.7 | -6.2 | -5.3 | -6.7 |
| Pioglitazone | -7.8 | -7.1 | -7.2 | -7.2 | -6.3 | -6.8 | -6.5 | -7.1 | -5.7 | -6.1 | -5.2 | -6.7 |
| Raltitrexed | -8.5 | -7.4 | -7.3 | -6.4 | -5.9 | -6.3 | -6.5 | -6.5 | -5.7 | -6.2 | -5.3 | -6.5 |
| Scriptaid | -8.1 | -7.5 | -7.7 | -6.8 | -6.3 | -6.3 | -6.3 | -6.4 | -5.4 | -6 | -5.6 | -6.5 |
| Coumestrol | -7.9 | -7.2 | -7.9 | -6.3 | -6.9 | -6.1 | -6.2 | -6.5 | -5.8 | -5.8 | -5.5 | -6.5 |
| FolicAcid | -8.1 | -7.4 | -7.1 | -6.2 | -7.5 | -6.2 | -5.9 | -6.1 | -6.4 | -6.2 | -5.3 | -6.5 |
| Parecoxib | -7.8 | -7.3 | -7.3 | -6.9 | -6.2 | -5.9 | -6.6 | -6.2 | -5.9 | -5.7 | -5.7 | -6.5 |
| Calcitriol | -7.3 | -7.6 | -6.9 | -6.5 | -6.1 | -6.6 | -6 | -6.1 | -6.6 | -6 | -5 | -6.4 |
| Topotecan | -8.1 | -7.4 | -7.1 | -6.5 | -6.4 | -6.1 | -5.9 | -6.1 | -5.7 | -6.3 | -5.1 | -6.4 |
| Sildenafil | -7.2 | -7.6 | -7.1 | -6.3 | -6.7 | -6.1 | -6.8 | -5.6 | -6.1 | -5.6 | -5.5 | -6.4 |
| Dexamethasone | -7.4 | -7.7 | -7.3 | -6.2 | -6.2 | -6.2 | -6 | -6.3 | -6.3 | -6 | -5.6 | -6.4 |
| Carbamazepine | -8.4 | -7.6 | -6.7 | -6.4 | -6.1 | -6.2 | -6.3 | -5.9 | -6.1 | -5.5 | -5.5 | -6.4 |
| Celecoxib | -8.3 | -2.9 | -7.1 | -6.9 | -6.3 | -6.2 | -7.1 | -6.2 | -6.6 | -6.1 | -5.7 | -6.3 |
| Quercetin | -7.6 | -7.6 | -6.3 | -6.1 | -6.4 | -5.8 | -6 | -6.2 | -6.1 | -5.9 | -5.3 | -6.2 |
| Sunitinib | -7.4 | -7.4 | -6.8 | -6.4 | -6.2 | -6.3 | -6.2 | -6.3 | -5.5 | -5.5 | -5.2 | -6.2 |
| Thalidomide | -8 | -6.8 | -6.8 | -6 | -6.5 | -5.8 | -6.2 | -6.1 | -6 | -5.4 | -5.6 | -6.2 |
| Ciglitazone | -8 | -8.2 | -6.7 | -6.4 | -6.1 | -5.9 | -5.7 | -5.7 | -5.6 | -5.6 | -5.1 | -6.2 |

| **Table S9.** Docking scores (binding affinities, kcal/mol) between the proposed receptors and T2D-control ligand | | | | | | | | | | | |
| --- | --- | --- | --- | --- | --- | --- | --- | --- | --- | --- | --- |
| **name** | **CD44** | **EFEMP1** | **TP53** | **COL18A1** | **THBS1** | **PLS3** | **CLDN5** | **EGR1** | **CAV1** | **SP1** | **PTK2** |
| Glibenclamide | -9.5 | -8.7 | -8.6 | -7.9 | -7.9 | -8.2 | -8.6 | -7.5 | -8.1 | -7.2 | -7.8 |
| Metformin | -9.6 | -8.9 | -7.9 | -8.6 | -8.3 | -8.9 | -8.0 | -7.1 | -7.6 | -7.6 | -8.1 |
| Gliclazide | -8.6 | -9.4 | -8.2 | -7.9 | -8.2 | -7.9 | -8.0 | -8.6 | -7.9 | -6.8 | -7.0 |

| **Table S10.** Docking scores (binding affinities, kcal/mol) between the proposed receptors and CRC-control ligand | | | | | | | | | | | |
| --- | --- | --- | --- | --- | --- | --- | --- | --- | --- | --- | --- |
| **name** | **CD44** | **EFEMP1** | **TP53** | **COL18A1** | **THBS1** | **PLS3** | **CLDN5** | **EGR1** | **CAV1** | **SP1** | **PTK2** |
| Irinotecan Hydrochloride | -9.1 | -8.6 | -9.8 | -7.9 | -8.5 | -8.1 | -8.9 | -7.9 | -8.5 | -7.2 | -7.9 |
| Capecitabine | -8.0 | -9.0 | -8.6 | -8.1 | -8.0 | -7.9 | -7.8 | -7.1 | -7.8 | -8.1 | -6.9 |
| Fruquintinib | -8.9 | -8.4 | -8.2 | -8.0 | -7.9 | -8.6 | -8.4 | -8.3 | -7.9 | -8.1 | -8.3 |

| **Table S11.** Molecular docking scores (binding affinities, kcal/mol) with T2D causing genes | | | | |
| --- | --- | --- | --- | --- |
| Gene/drug | Irinotecan | Leucovorincalcium | Regorafenib | Fenretinide |
| APOE | -8.0 | -8.0 | -8.6 | -8.8 |
| SCARB1 | -8.9 | -8.5 | -8.2 | -8.0 |
| EGF1 | -8.1 | -7.4 | -7.9 | -8.3 |
| COL1A1 | -9.4 | -8.1 | -8.2 | -7.8 |
| CDC42 | -8.2 | -7.8 | -8.1 | -8.0 |
| FYN | -8.6 | -7.9 | -9.0 | -8.2 |

| **Table S12.** Molecular docking scores (binding affinities, kcal/mol) with CRC causing genes | | | | |
| --- | --- | --- | --- | --- |
| Gene/drug | Irinotecan | Leucovorincalcium | Regorafenib | Fenretinide |
| CENPF | -8.9 | -9.3 | -7.9 | -8.2 |
| NCAPG | -9.2 | -8.9 | -9.1 | -8.3 |
| CDK1 | -8.5 | -8.8 | -8.8 | -7.6 |
| CCNA2 | -9.6 | -7.9 | -9.2 | -8.9 |

| **Table S13.** Molecular docking scores (binding affinities, kcal/mol) with equal expressed genes or, unregulated genes that are not associated with T2D or CRC | | | | |
| --- | --- | --- | --- | --- |
| Gene/drug | Irinotecan | Leucovorincalcium | Regorafenib | Fenretinide |
| ADH1B | 5.9 | 6.3 | 5.0 | 5.9 |
| CHGA | 5.2 | 5.4 | 5.3 | 5.8 |
| PPP1R3C | 6.7 | 5.2 | 4.1 | 5.7 |
| RFC3 | 5.8 | 6.1 | 5.2 | 4.2 |
| IP6K3 | 6.2 | 5.8 | 6.0 | 5.9 |

| **Table S14.** Some important docking results with the protein-ligand complexes. First, second and third columns indicate target proteins, drug molecules and their binding affinities, respectively. Fourth and fifth column indicate 2D and 3D view of the drug-target complexes, and 6^th^ column indicates the interacting amino acids in the complexes. | | | | | |
| --- | --- | --- | --- | --- | --- |
| **Potential Target** | **Structure of Lead Compounds** | **Binding Affinity(kCal/mol)** | **Target-Ligand Interaction (2D-view)** | **Target-Ligand Interaction (3D-view)** | **Interacting Amino Acids** |
| CD44 | 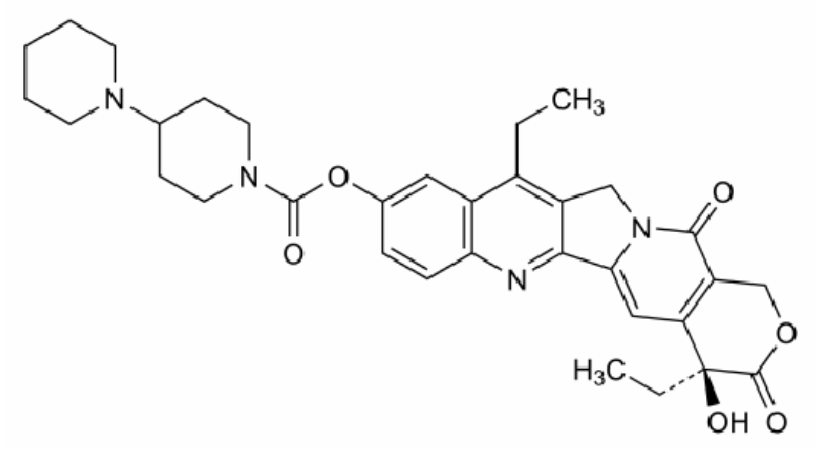  Irinotecan | -9.4 | 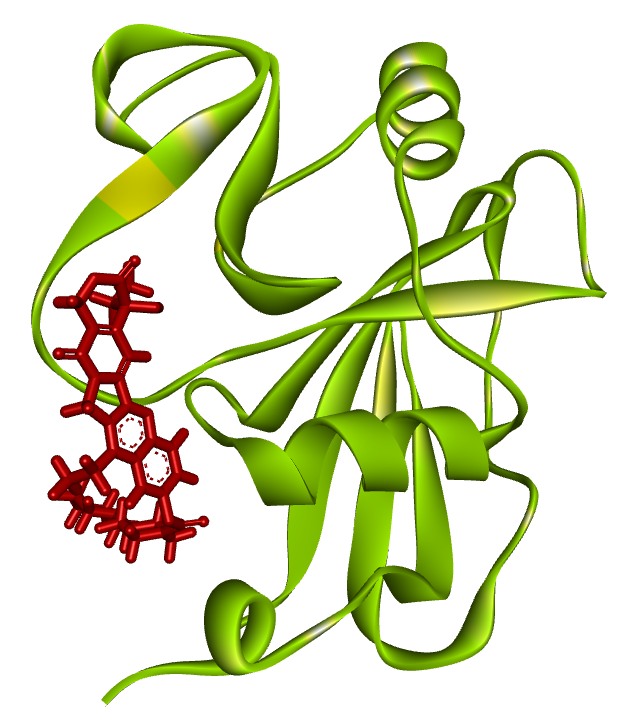 | 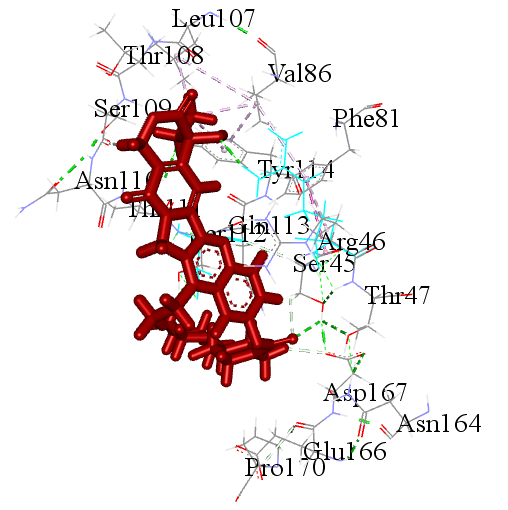 | Leu107,Thr108,Val86, Phe81,Ser109, Tyr114, Asn110,Thr111,Gln113,Arg46, Ser45, Thr47, Asp167,Asn164,Glu166, Pro170 |
| EFEMP1 | 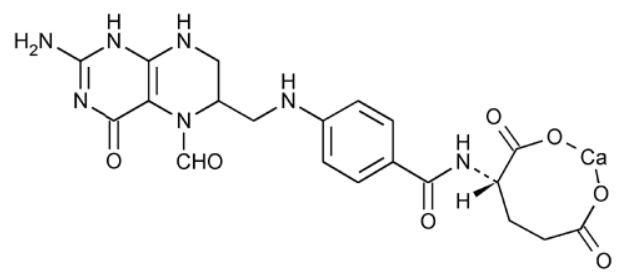  Leucovorin calcium | -8.2 | 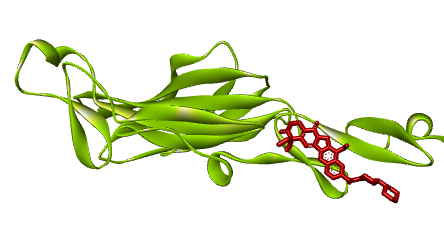 | 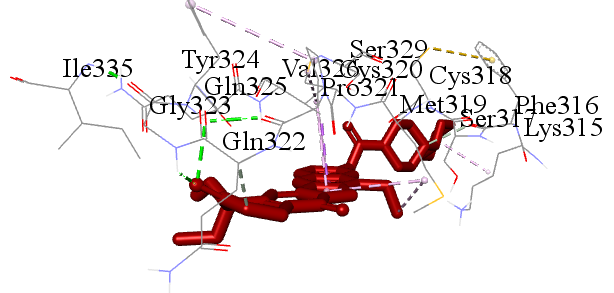 | Ile335,Tyr324,Gln325,Gly323,Gln322,Ser329,Cys318,Val326,Cys320,Pro321,Met319,Phe316,Lys315,Ser317, |
| TP53 | 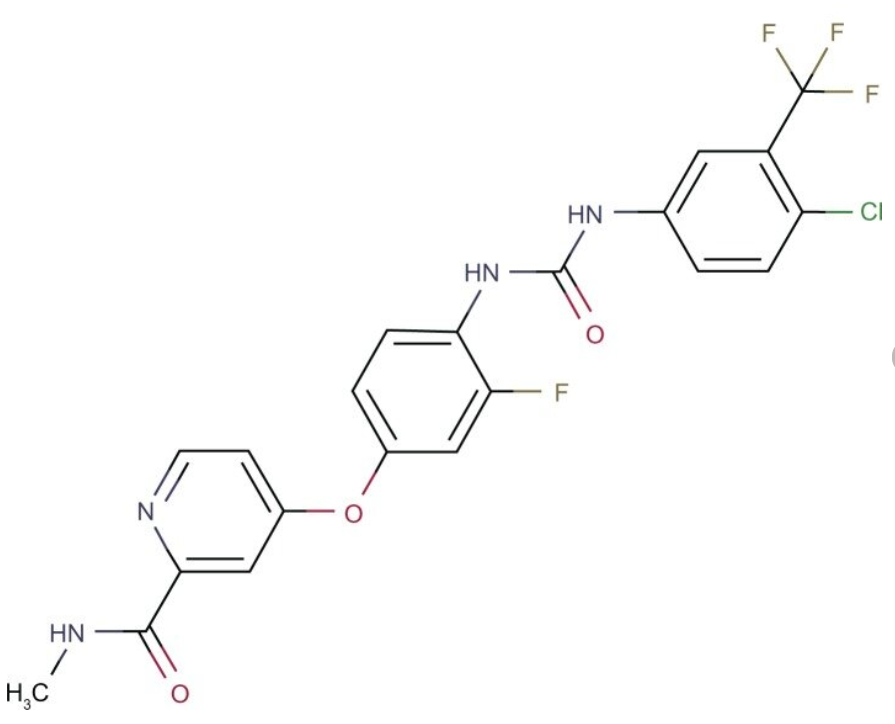  Regorafenib | **-8.0** | 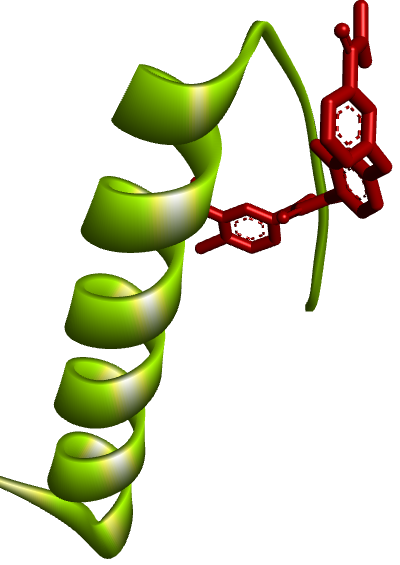 | 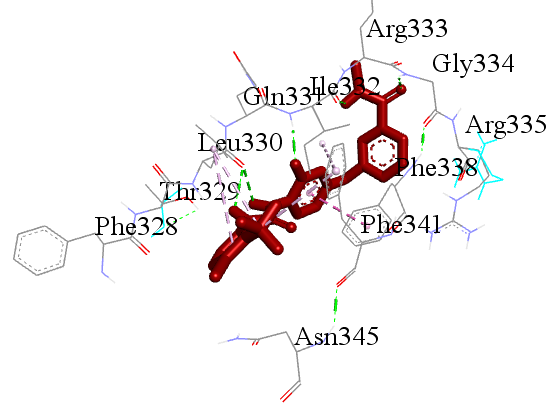 | Arg333,Gly334,Ile332,Gln331,Arg335,Leu330,Thr329,Phe328,Phe341,Asn345,The338, |
| COL18A1 | 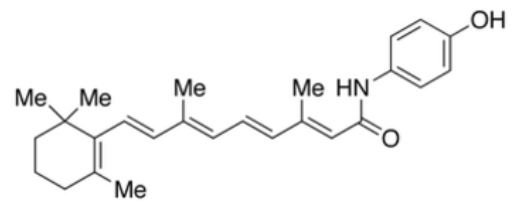  Fenretinide | **-7.1** | 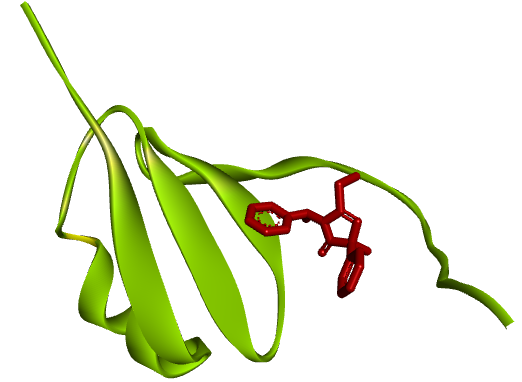 | 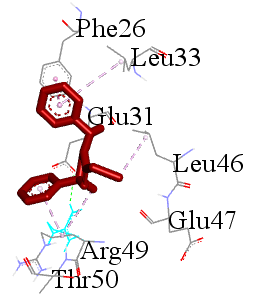 | Phe26,Leu33,Glu31,Leu46,Glu47,Arg49,Thr50 |

| **Table S15.** Before docking the physio-chemical descriptors, Frontier molecular orbitals and their reactivity descriptor analysis of top-ranked 4 compounds | | | | |
| --- | --- | --- | --- | --- |
|  | **Irinotecan** | **Leucovorincalcium** | **Regorafenib** | **Fenretinide** |
| ԑHOMO | -0.20987 | -0.06731 | -0.23652 | -0.19065 |
| ԑLUMO | -0.08206 | -0.05779 | -0.05684 | -0.07400 |
| Energy gap  Δԑ = ԑLUMO – ԑHOMO) | 0.12781 | 0.00952 | 0.17968 | 0.11665 |
| Ionization potential  (I = ˗ԑHOMO) | 0.20987 | 0.06731 | 0.23652 | 0.19065 |
| Electron affinity  (A = ˗ԑLUMO) | 0.08206 | 0.05779 | 0.05684 | 0.07400 |
| Electro-negativity  (χ = (I+A)/2) | 0.145965 | 0.06255 | 0.14668 | 0.132325 |
| Chemical potential  (µ = ˗(I+A)/2) | -0.145965 | -0.06255 | -0.14668 | -0.132325 |
| Chemical hardness  (ղ = (I-A)/2) | 0.063905 | 0.06250 | 0.08984 | 0.058325 |
| Electrophilicity index  (ω = μ^2^/2η) | 0.1669 | 0.4103 | 0.1195 | 0.1509 |
| Softness  (S = 1/ղ) | 15.644 | 15.983 | 11.138 | 6.625 |

| **Table S16.** After docking the physio-chemical descriptors, Frontier molecular orbitals and their reactivity descriptor analysis of top-ranked 4 compounds | | | | |
| --- | --- | --- | --- | --- |
|  | **Irinotecan** | **Leucovorincalcium** | **Regorafenib** | **Fenretinide** |
| ԑHOMO | -0.20986 | -0.10524 | -0.23231 | -0.19820 |
| ԑLUMO | -0.08206 | -0.08001 | -0.04746 | -0.02084 |
| Energy gap  Δԑ = ԑLUMO – ԑHOMO) | 0.12780 | 0.02523 | 0.18485 | 0.17736 |
| Ionization potential  (I = ˗ԑHOMO) | 0.20986 | 0.10524 | 0.23231 | 0.19820 |
| Electron affinity  (A = ˗ԑLUMO) | 0.08206 | 0.08001 | 0.04746 | 0.02084 |
| Electro-negativity  (χ = (I+A)/2) | 0.14623 | 0.092625 | 0.139885 | 0.109520 |
| Chemical potential  (µ = ˗(I+A)/2) | **-**0.14596 | **-**0.092625 | **-**0.139885 | **-**0.109520 |
| Chemical hardness  (ղ = (I-A)/2) | 0.06363 | 0.012615 | 0.092425 | 0.0886 |
| Electrophilicity index  (ω = μ^2^/2η) | 0.167409 | 0.340041 | 0.10584 | 0.06768 |
| Softness  (S = 1/ղ) | 15.710 | 79.270 | 10.815 | 11.2862 |

| **Table S17.** After Molecular Dynamic (MD) simulations the physio-chemical descriptors, Frontier molecular orbitals and their reactivity descriptor analysis of top-ranked 4 compounds | | | | |
| --- | --- | --- | --- | --- |
|  | **Irinotecan** | **Leucovorincalcium** | **Regorafenib** | **Fenretinide** |
| ԑHOMO | -0.12873 | -0.10877 | -0.22961 | -0.19018 |
| ԑLUMO | -0.03909 | -0.07591 | -0.06755 | -0.07712 |
| Energy gap  Δԑ = ԑLUMO – ԑHOMO) | 0.08964 | 0.03286 | 0.162060 | 0.11306 |
| Ionization potential  (I = ˗ԑHOMO) | 0.12873 | 0.10877 | 0.22961 | 0.19018 |
| Electron affinity  (A = ˗ԑLUMO) | 0.03909 | 0.07591 | 0.06755 | 0.07712 |
| Electro-negativity  (χ = (I+A)/2) | 0.08391 | 0.09234 | 0.14858 | 0.13365 |
| Chemical potential  (µ = ˗(I+A)/2) | **-**0.08391 | -0.09234 | **-**0.14858 | **-**0.13365 |
| Chemical hardness  (ղ = (I-A)/2) | 0.04482 | 0.01643 | 0.08103 | 0.05653 |
| Electrophilicity index  (ω = μ^2^/2η) | 0.078545 | 0.259484 | 0.136224 | 0.15792 |
| Softness  (S = 1/ղ) | 22.311 | 60.867 | 12.343 | 17.687 |

**References**

[1] M.S. Lee, C.C. Hsu, M.L. Wahlqvist, H.N. Tsai, Y.H. Chang, Y.C. Huang, Type 2 diabetes increases and metformin reduces total, colorectal, liver and pancreatic cancer incidences in Taiwanese: A representative population prospective cohort study of 800,000 individuals, BMC Cancer. 11 (2011). https://doi.org/10.1186/1471-2407-11-20.

[2] C.R. Garrett, H.M. Hassabo, N.A. Bhadkamkar, S. Wen, V. Baladandayuthapani, B.K. Kee, C. Eng, M.M. Hassan, Survival advantage observed with the use of metformin in patients with type II diabetes and colorectal cancer, Br. J. Cancer. 106 (2012) 1374–1378. https://doi.org/10.1038/bjc.2012.71.

[3] S.L. Bowker, S.R. Majumdar, P. Veugelers, J.A. Johnson, Increased cancer-related mortality for patients with type 2 diabetes who use sulfonylureas or insulin, Diabetes Care. 29 (2006) 254–258. https://doi.org/10.2337/diacare.29.02.06.dc05-1558.

[4] A.J. Krentz, M.B. Patel, C.J. Bailey, New Drugs for Type 2 Diabetes Mellitus, Drugs. 68 (2008) 2131–2162. https://doi.org/10.2165/00003495-200868150-00005.

[5] S. Padhi, A.K. Nayak, A. Behera, Type II diabetes mellitus: a review on recent drug based therapeutics, Biomed. Pharmacother. 131 (2020). https://doi.org/10.1016/j.biopha.2020.110708.

[6] M.P. Mosharaf, M.S. Reza, M.K. Kibria, F.F. Ahmed, M.H. Kabir, S. Hasan, M.N.H. Mollah, Computational identification of host genomic biomarkers highlighting their functions, pathways and regulators that influence SARS-CoV-2 infections and drug repurposing, Sci. Rep. 12 (2022). https://doi.org/10.1038/s41598-022-08073-8.

[7] R.A. Harrigan, M.S. Nathan, P. Beattie, Oral agents for the treatment of type 2 diabetes mellitus: Pharmacology, toxicity, and treatment, Ann. Emerg. Med. 38 (2001) 68–78. https://doi.org/10.1067/mem.2001.114314.

[8] M. Avery, C. Mizuno, A. Chittiboyina, T. Kurtz, H. Pershadsingh, Type 2 Diabetes and Oral Antihyperglycemic Drugs, Curr. Med. Chem. 15 (2008) 61–74. https://doi.org/10.2174/092986708783330656.

[9] R.J. McNellis, V. Beswick-Escanlar, Aspirin use for the primary prevention of cardiovascular disease and colorectal cancer, Am. Fam. Physician. 94 (2016) 661–662.

[10] T.O. Yau, Precision treatment in colorectal cancer: Now and the future, JGH Open. 3 (2019) 361–369. https://doi.org/10.1002/jgh3.12153.

[11] U. Hani, Y.K. Honnavalli, M.Y. Begum, S. Yasmin, R.A.M. Osmani, M.Y. Ansari, Colorectal cancer: A comprehensive review based on the novel drug delivery systems approach and its management, J. Drug Deliv. Sci. Technol. 63 (2021). https://doi.org/10.1016/j.jddst.2021.102532.

[12] F. Geng, Z. Wang, H. Yin, J. Yu, B. Cao, Molecular Targeted Drugs and Treatment of Colorectal Cancer: Recent Progress and Future Perspectives, Cancer Biother. Radiopharm. 32 (2017) 149–160. https://doi.org/10.1089/cbr.2017.2210.

[13] C. Tao, J. Sun, W.J. Zheng, J. Chen, H. Xu, Colorectal cancer drug target prediction using ontology-based inference and network analysis, Database. 2015 (2015). https://doi.org/10.1093/database/bav015.

[14] L.B. Saltz, Value in colorectal cancer treatment: Where it is lacking, and why, Cancer J. (United States). 22 (2016) 232–235. https://doi.org/10.1097/PPO.0000000000000194.

[15] P.A. Shuvo, A. Tahsin, M.M. Rahman, T. Bin Emran, Dostarlimab: The miracle drug for the treatment of colorectal cancer, Ann. Med. Surg. 81 (2022). https://doi.org/10.1016/j.amsu.2022.104493.

[16] Y. Yang, P. Liu, M. Zhou, L. Yin, M. Wang, T. Liu, X. Jiang, H. Gao, Small-molecule drugs of colorectal cancer: Current status and future directions, Biochim. Biophys. Acta - Mol. Basis Dis. 1870 (2024). https://doi.org/10.1016/j.bbadis.2023.166880.

[17] L.P. Rivory, New drugs for colorectal cancer - Mechanisms of action, Aust. Prescr. 25 (2002) 108–110. https://doi.org/10.18773/austprescr.2002.110.

[18] P. Nowak-Sliwinska, L. Scapozza, A.R. i. Altaba, Drug repurposing in oncology: Compounds, pathways, phenotypes and computational approaches for colorectal cancer, Biochim. Biophys. Acta - Rev. Cancer. 1871 (2019) 434–454. https://doi.org/10.1016/j.bbcan.2019.04.005.

[19] J.J.M. Kwakman, C.J.A. Punt, Oral drugs in the treatment of metastatic colorectal cancer, Expert Opin. Pharmacother. 17 (2016) 1351–1361. https://doi.org/10.1080/14656566.2016.1186649.

[20] X. Tan, L. Gong, X. Li, X. Zhang, J. Sun, X. Luo, Q. Wang, J. Chen, L. Xie, S. Han, Promethazine inhibits proliferation and promotes apoptosis in colorectal cancer cells by suppressing the PI3K/AKT pathway, Biomed. Pharmacother. 143 (2021). https://doi.org/10.1016/j.biopha.2021.112174.

[21] M. Komiya, G. Fujii, M. Takahashi, M. Iigo, M. Mutoh, Prevention and intervention trials for colorectal cancer, Jpn. J. Clin. Oncol. 43 (2013) 685–694. https://doi.org/10.1093/jjco/hyt053.

[22] R.D. Church, J.W. Fleshman, H.L. McLeod, Cyclo-oxygenase 2 inhibition in colorectal cancer therapy, Br. J. Surg. 90 (2003) 1055–1067. https://doi.org/10.1002/bjs.4297.

[23] S.L. Freshour, S. Kiwala, K.C. Cotto, A.C. Coffman, J.F. McMichael, J.J. Song, M. Griffith, O.L. Griffith, A.H. Wagner, Integration of the Drug-Gene Interaction Database (DGIdb 4.0) with open crowdsource efforts, Nucleic Acids Res. 49 (2021) D1144–D1151. https://doi.org/10.1093/nar/gkaa1084.

[24] A. V. Moiyadi, E. Sridhar, δ-Aminolevulinic acid-induced fluorescence unmasks biological intratumoral heterogeneity within histologically homogeneous areas of malignant gliomas, Acta Neurochir. (Wien). 157 (2015) 617–619. https://doi.org/10.1007/s00701-014-2321-4.

[25] D.S. Wishart, C. Knox, A.C. Guo, D. Cheng, S. Shrivastava, D. Tzur, B. Gautam, M. Hassanali, DrugBank: A knowledgebase for drugs, drug actions and drug targets, Nucleic Acids Res. 36 (2008). https://doi.org/10.1093/nar/gkm958.
